# Supplementary material for: Antagonistic Inflammatory Phenotypes Dictate Tumor Fate and Response to Immune Checkpoint Blockade
Source: Immunity. 2020 Dec 15;53(6):1215–1229.e8. doi: 10.1016/j.immuni.2020.10.020 (PMC7772804; doi:10.1016/j.immuni.2020.10.020)
Supplement: Document S1. Figures S1–S7 [file mmc1.pdf]

Immunity, Volume *53*

Supplemental Information

Antagonistic Inflammatory Phenotypes
Dictate Tumor Fate and Response
to Immune Checkpoint Blockade

Eduardo Bonavita, Christian P. Bromley, Gustav Jonsson, Victoria S. Pelly, Sudhakar Sahoo, Katherine Walwyn-Brown, Sofia Mensurado, Agrin Moeini, Eimear Flanagan, Charlotte R. Bell, Shih-Chieh Chiang, C.P. Chikkanna-Gowda, Neil Rogers, Bruno Silva-Santos, Sebastien Jaillon, Alberto Mantovani, Caetano Reis e Sousa, Nadia Guerra, Daniel M. Davis, and Santiago Zelenay

Figure S1

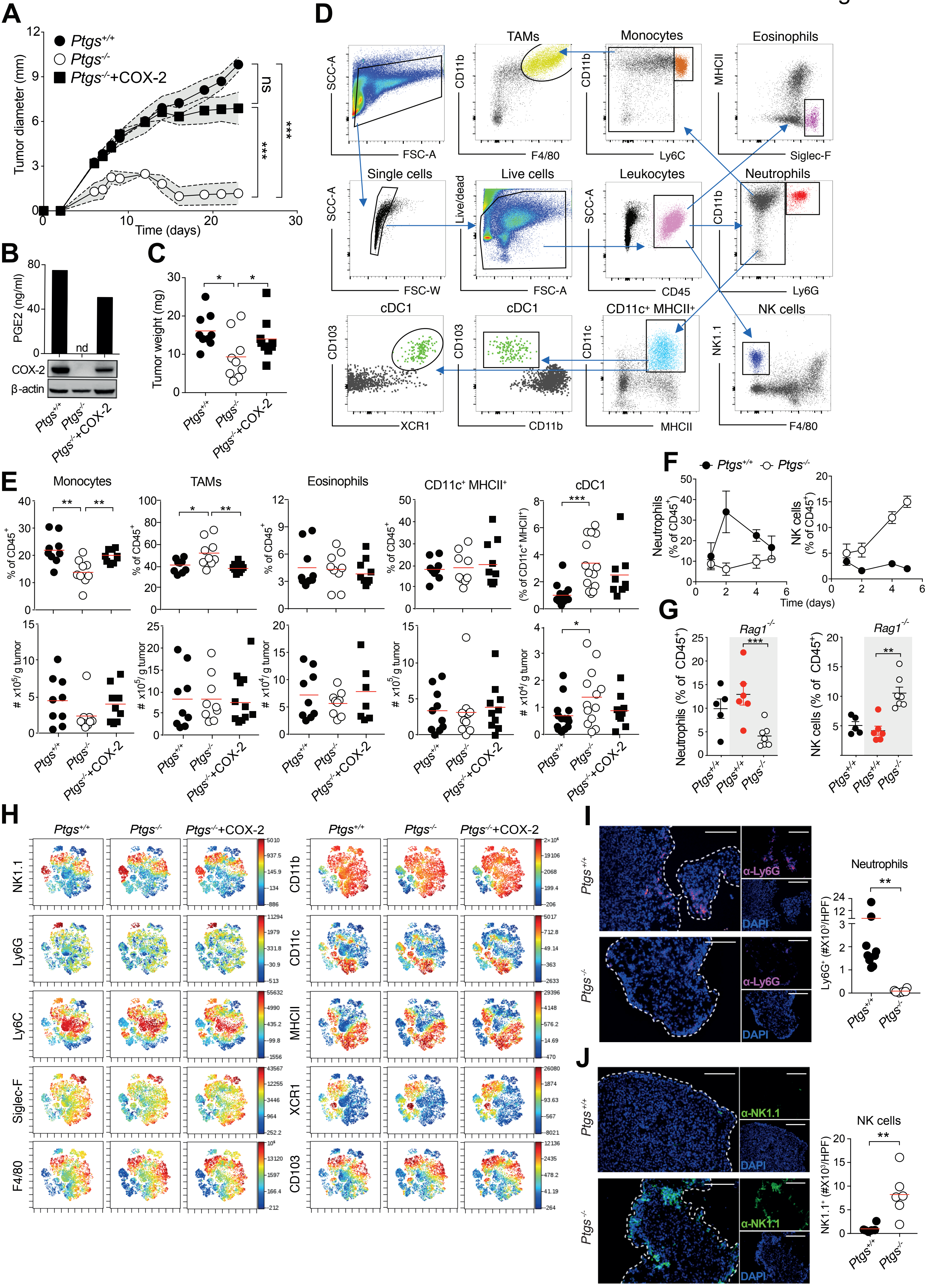
A
Ptgs+/+
Ptgs-/-
Ptgs-/-+COX-2
Tumor diameter (mm)
Time (days)
ns
***
***
B
PGE2 (ng/ml)
nd
COX-2
β-actin
Ptgs+/+
Ptgs-/-
Ptgs-/-+COX-2
C
Tumor weight (mg)
*
*
D
TAMs
Monocytes
Eosinophils
Single cells
Live cells
Leukocytes
Neutrophils
cDC1
CD11c+ MHCII+
NK cells
SCC-A
FSC-A
CD11b
F4/80
Ly6C
MHCII
Siglec-F
FSC-W
Live/dead
CD45
Ly6G
CD103
XCR1
CD11c
NK1.1
E
Monocytes
TAMs
Eosinophils
CD11c+ MHCII+
cDC1
% of CD45+
(% of CD11c+ MHCII+)
x10^5/ g tumor
x10^4/ g tumor
F
Neutrophils (% of CD45+)
NK cells (% of CD45+)
Time (days)
G
Rag1-/-
Neutrophils (% of CD45+)
NK cells (% of CD45+)
H
NK1.1
Ly6G
Ly6C
Siglec-F
F4/80
CD11b
CD11c
MHCII
XCR1
CD103
I
α-Ly6G
DAPI
Neutrophils
Ly6G+ (#X10^3/HPF)
J
α-NK1.1
NK cells
NK1.1+ (#X10^3/HPF)

Figure S1. (Related to Figure 1) Genetic ablation of cancer cell-intrinsic COX alters the intratumoral accumulation of select innate immune cells. (A) Growth profile of tumors following implantation of *Ptgs*$^{+/+}$, *Ptgs*$^{-/-}$ and *Ptgs*$^{-/-}$ + COX-2 melanoma cells into wild-type mice. (B) PGE2 production and COX-2 expression by cells used in A. (C) Weight of *Ptgs*$^{+/+}$, *Ptgs*$^{-/-}$ and *Ptgs*$^{-/-}$ + COX-2 tumors four days after melanoma cell implantation. (D) Representative gating strategy. (E) Immune cell infiltrate analysis of tumors formed by *Ptgs*$^{+/+}$, *Ptgs*$^{-/-}$ and *Ptgs*$^{-/-}$ + COX-2 melanoma cells four days post-implantation. The frequency and the number of intratumoral monocytes, TAMs, eosinophils, CD11c^{+} MHCII^{+} cells and cDC1s are shown. (F) Kinetics of neutrophil and NK cell accumulation in *Ptgs*$^{+/+}$ and *Ptgs*$^{-/-}$ tumors. (G) Frequency of infiltrating leukocytes one-month post-cancer cell implantation in wild-type (injected with *Ptgs*$^{+/+}$ cells) *or Rag1*$^{-/-}$ (injected with either *Ptgs*$^{+/+}$ or *Ptgs*$^{-/-}$ cells). Neutrophils and NK cells are gated on live CD45^{+} cells. (H) t-SNE projections of individual markers used in Figure 1A. Each plot is obtained from six concatenated samples. (I-J) Immunofluorescence analysis of neutrophils (I) and NK cells (J) in tumors formed by *Ptgs*$^{+/+}$ and *Ptgs*$^{-/-}$ melanoma cells four days post implantation (scale bar 100 μm). Data are expressed as mean ± SEM, two-way ANOVA (A), one-way ANOVA (C, E, G), unpaired Student's t test (I-J).

Figure S2

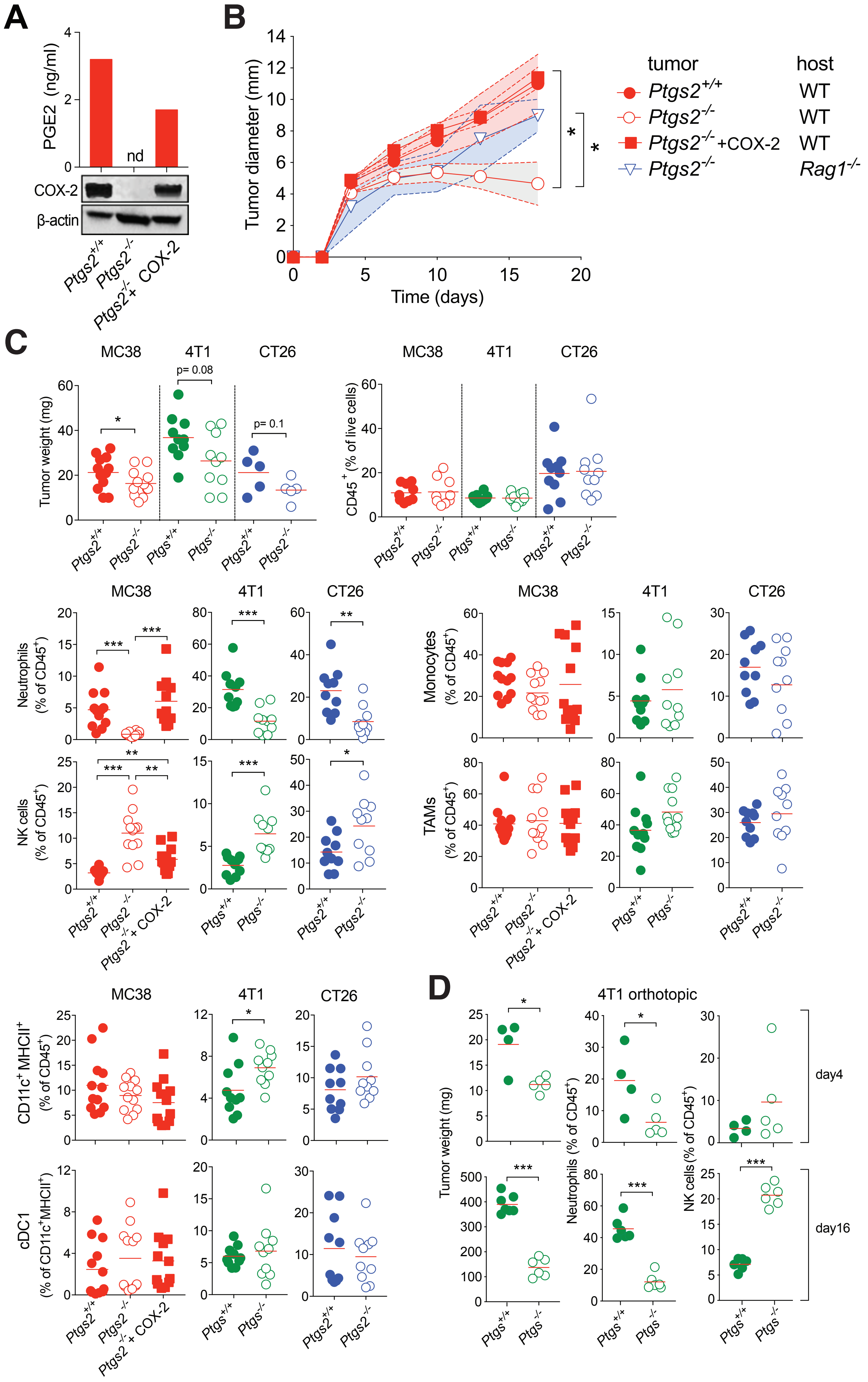

A
B
C
D
PGE2 (ng/ml)
nd
COX-2
β-actin
Tumor diameter (mm)
Time (days)
tumor
host
WT
Rag1-/-
Tumor weight (mg)
CD45+ (% of live cells)
MC38
4T1
CT26
p= 0.08
p= 0.1
Neutrophils (% of CD45+)
NK cells (% of CD45+)
Monocytes (% of CD45+)
TAMs (% of CD45+)
CD11c+ MHCII+ (% of CD45+)
cDC1 (% of CD11c+MHCII+)
4T1 orthotopic
day4
day16

Figure S2. (Related to Figure 1) Neutrophil and NK cell accumulation within the TME is regulated by cancer cell-intrinsic COX activity in various cancer models. (A) PGE2 production and COX-2 expression by *Ptgs2*$^{+/+}$, *Ptgs2*$^{-/-}$ and *Ptgs2*$^{-/-}$ + COX-2 MC38 colorectal cancer cells. (B) Growth profile of *Ptgs2*$^{+/+}$, *Ptgs2*$^{-/-}$ and *Ptgs2*$^{-/-}$ + COX-2 MC38 cancer cells in wild-type and *Rag1*$^{-/-}$ mice. (C) Weight of tumors formed by *Ptgs2*$^{+/+}$ *or Ptgs2*$^{-/-}$ MC38 colorectal, 4T1 (*Ptgs*$^{+/+}$ and *Ptgs* $^{-/-}$) breast and CT26 colorectal cells four days post-implantation. Immune cell infiltrate analysis of tumors formed by *Ptgs2*$^{+/+}$, *Ptgs2*$^{-/-}$ and *Ptgs2*$^{-/-}$ + COX-2 MC38 and CT26 colorectal and *Ptgs*$^{+/+}$ and *Ptgs*$^{-/-}$ 4T1 breast cancer cells four days post-implantation. The frequency of CD45^{+} cells, neutrophils, NK cells, monocytes, TAMs, CD11c^{+} MHCII^{+} cells and cDC1s is shown. (D) Tumor weight, NK cell and neutrophil frequencies in *Ptgs*$^{+/+}$and *Ptgs*$^{-/-}$ orthotopic 4T1 tumors on days 4 and 16 post injection. Data are expressed as mean ± SEM, two-way ANOVA (B), one-way ANOVA or unpaired Student's t test (C-D).

Figure S3

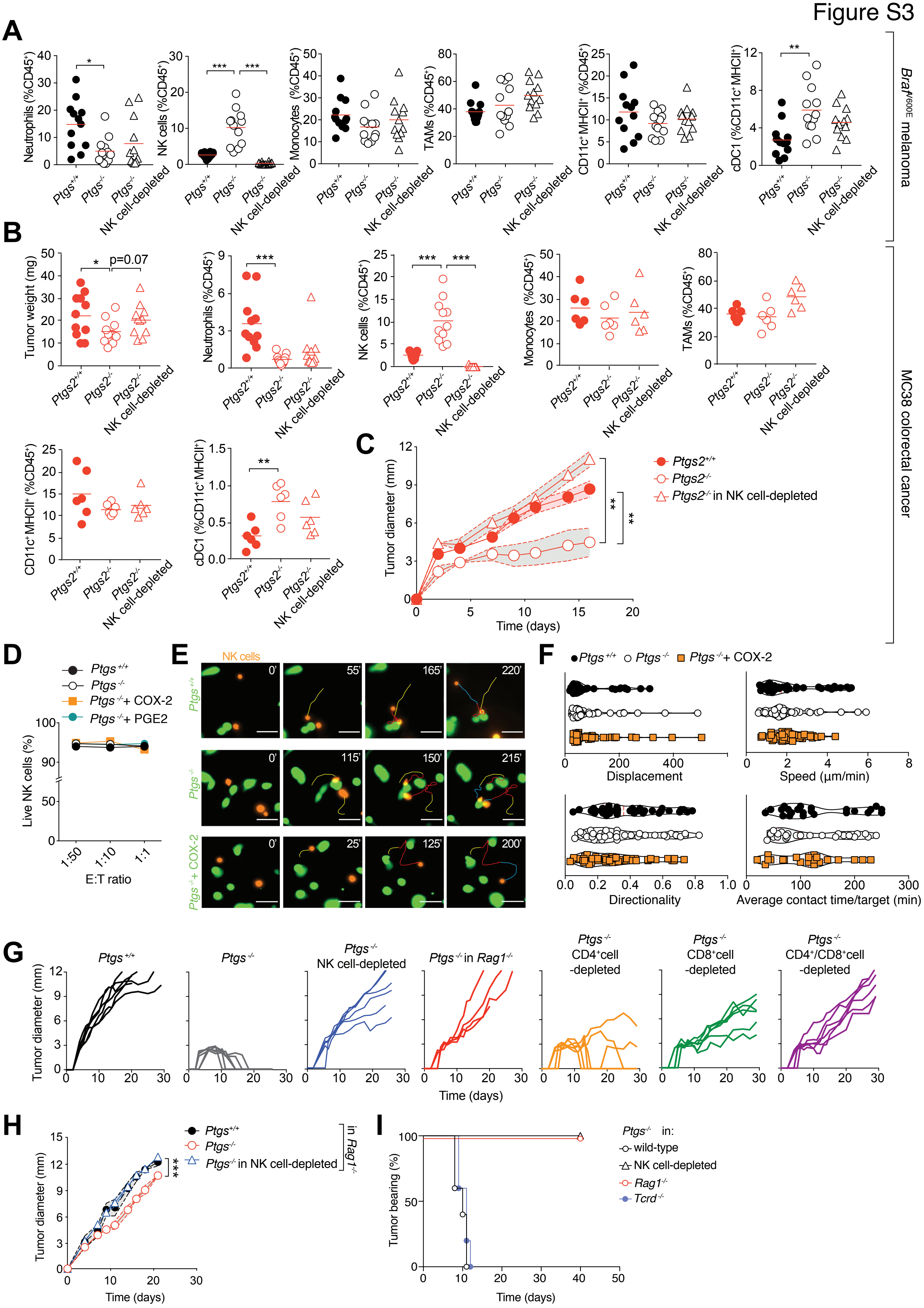

A
Neutrophils (%CD45+)
NK cells (%CD45+)
Monocytes (%CD45+)
TAMs (%CD45+)
CD11c+ MHCII+ (%CD45+)
cDC1 (%CD11c+ MHCII+)
Ptgs+/+
Ptgs-/-
Ptgs-/- NK cell-depleted
Braf V600E melanoma
B
Tumor weight (mg)
p=0.07
Neutrophils (%CD45+)
NK cells (%CD45+)
Monocytes (%CD45+)
TAMs (%CD45+)
CD11c+ MHCII+ (%CD45+)
cDC1 (%CD11c+ MHCII+)
Ptgs2+/+
Ptgs2-/-
Ptgs2-/- NK cell-depleted
C
Tumor diameter (mm)
Time (days)
Ptgs2+/+
Ptgs2-/-
Ptgs2-/- in NK cell-depleted
MC38 colorectal cancer
D
Ptgs+/+
Ptgs-/-
Ptgs-/- + COX-2
Ptgs-/- + PGE2
Live NK cells (%)
E:T ratio
1:50
1:10
1:1
E
NK cells
Ptgs+/+
Ptgs-/-
Ptgs-/- + COX-2
0'
55'
165'
220'
115'
150'
215'
25'
125'
200'
F
Ptgs+/+
Ptgs-/-
Ptgs-/- + COX-2
Displacement
Speed (µm/min)
Directionality
Average contact time/target (min)
G
Ptgs+/+
Ptgs-/-
Ptgs-/- NK cell-depleted
Ptgs-/- in Rag1-/-
Ptgs-/- CD4+cell -depleted
Ptgs-/- CD8+cell -depleted
Ptgs-/- CD4+/CD8+cell -depleted
Tumor diameter (mm)
Time (days)
H
Ptgs+/+
Ptgs-/-
Ptgs-/- in NK cell-depleted
in Rag1-/-
Tumor diameter (mm)
Time (days)
I
Ptgs-/- in:
wild-type
NK cell-depleted
Rag1-/-
Tcrd-/-
Tumor bearing (%)
Time (days)

Figure S3. (Related to Figure 1 and Figure 2) NK cells are essential for both T cell-independent and T cell-dependent tumor control. (A) Neutrophil, NK cell, monocyte, CD11c^{+} MHCII^{+} cell, TAM and cDC1 frequency in *Ptgs*$^{+/+}$ and *Ptgs*$^{-/-}$ tumors four days post-implantation in NK cell-competent or depleted mice. (B) Tumor weight and immune cell infiltrate in *Ptgs2*$^{+/+}$ and *Ptgs2*$^{-/-}$ MC38 tumors four days after cell implantation in NK cell-competent or depleted mice. (C) Growth profile of *Ptgs2*$^{+/+}$ and *Ptgs2*$^{-/-}$ MC38 colorectal cancer cells implanted into NK cell-competent or -depleted mice. Data are expressed as mean ± SEM one-way (A-B) or two-way ANOVA (C). (D) Percentage of viable NK cells after the killing assay performed in coculture with *Ptgs*$^{+/+}$*, Ptgs*$^{-/-}$ (+/- synthetic PGE2) and *Ptgs*$^{-/-}$ + COX-2 melanoma cells. (E) Migration tracking of NK cells in co-cultures with *Ptgs*$^{+/+}$, *Ptgs*$^{-/-}$ or *Ptgs2*$^{-/-}$ + COX-2 melanoma cells (scale bar 50 µm, time expressed in minutes). (F) Migration parameters of NK cells co-cultured with *Ptgs*$^{+/+}$, *Ptgs*$^{-/-}$ or *Ptgs2*$^{-/-}$ + COX-2 targets showing the average contact time between NK cells and target cells, displacement, speed and directionality. (G) Individual growth profiles of *Ptgs*$^{+/+}$ and *Ptgs*$^{-/-}$ melanoma cells implanted into *Rag1*$^{-/-}$ or wild-type mice untreated or depleted of NK, CD4^{+} and/or CD8^{+} cells shown in Figure 2A. (H) Growth profile of *Ptgs*$^{+/+}$ and *Ptgs*$^{-/-}$ tumors in *Rag1*$^{-/-}$ or *Ptgs*$^{-/-}$ tumors in *Rag1*$^{-/-}$ depleted of NK cells, two-way ANOVA. (I) Kaplan-Meier plots showing the fraction of tumor- bearing wild-type (n=5), *Rag1*$^{-/-}$ (n=5), NK cell-depleted (n=11) and *Tcrd*$^{-/-}$ (n=5) mice injected with *Ptgs*$^{-/-}$ melanoma cells.

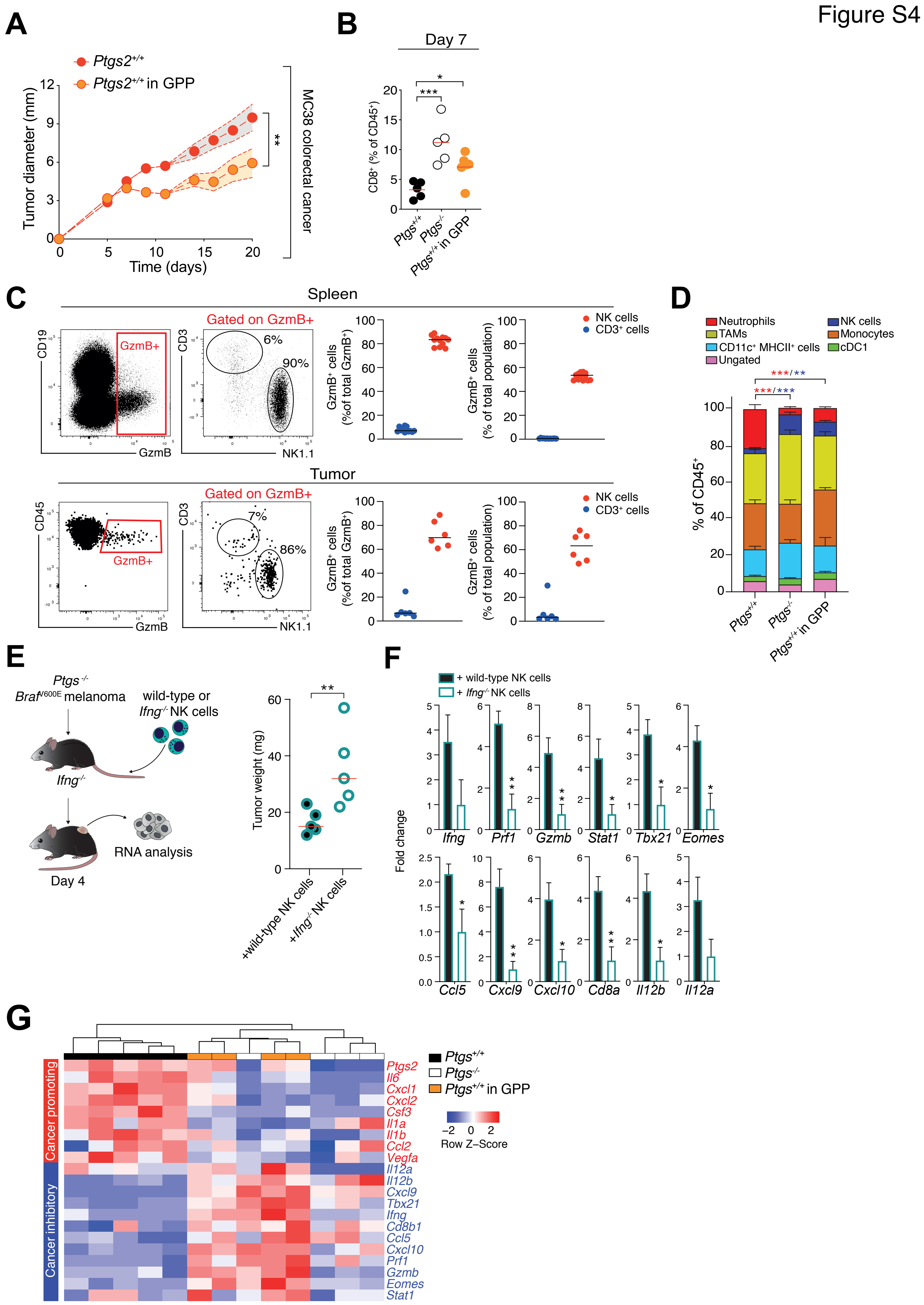
Figure S4
A
Ptgs2+/+
Ptgs2+/+ in GPP
Tumor diameter (mm)
Time (days)
MC38 colorectal cancer
B
Day 7
CD8+ (% of CD45+)
Ptgs+/+
Ptgs-/-
Ptgs+/+ in GPP
C
Spleen
Gated on GzmB+
GzmB+
CD19
GzmB
CD3
NK1.1
6%
90%
GzmB+ cells (%of total GzmB+)
GzmB+ cells (% of total population)
NK cells
CD3+ cells
Tumor
CD45
7%
86%
D
Neutrophils
TAMs
CD11c+ MHCII+ cells
Ungated
NK cells
Monocytes
cDC1
% of CD45+
E
Ptgs-/-
BrafV600E melanoma
wild-type or
Ifng-/- NK cells
Ifng-/-
Day 4
RNA analysis
Tumor weight (mg)
+wild-type NK cells
+Ifng-/- NK cells
F
+ wild-type NK cells
+ Ifng-/- NK cells
Fold change
Ifng
Prf1
Gzmb
Stat1
Tbx21
Eomes
Ccl5
Cxcl9
Cxcl10
Cd8a
Il12b
Il12a
G
Ptgs+/+
Ptgs-/-
Ptgs+/+ in GPP
Row Z-Score
Cancer promoting
Cancer inhibitory
Ptgs2
Il6
Cxcl1
Cxcl2
Csf3
Il1a
Il1b
Ccl2
Vegfa
Il12a
Il12b
Cxcl9
Tbx21
Ifng
Cd8b1
Ccl5
Cxcl10
Prf1
Gzmb
Eomes
Stat1

Figure S4. (Related to Figure 2 and Figure 3) Dual ablation of EP2 and EP4 on NK cells unleashed tumor control and the inflammatory TME switch in COX-competent tumors. (A) Growth profile of *Ptgs2*$^{+/+}$ MC38 colorectal cancer cells in wild-type and GPP mice. (B) Frequency of CD8^{+} T cells gated on live, CD45^{+}, CD3ε^{+} cells in *Ptgs*$^{+/+}$ and *Ptgs*$^{-/-}$ tumors in wild-type or *Ptgs*$^{+/+}$ tumors in GPP mice analyzed seven days post-implantation. (C) Representative plots and frequency of NK1.1^{+} and CD3ε^{+} cells within total GzmB^{+} cells and frequency of GzmB^{+} NK1.1^{+} and GzmB^{+} CD3ε^{+} cells in spleen or within *Ptgs*$^{+/+}$ tumors in naïve or in wild-type mice four days post-implantation, respectively. (D) Percentage of innate immune cell populations in *Ptgs*$^{+/+}$ and *Ptgs*$^{-/-}$ tumors in wild-type or *Ptgs*$^{+/+}$ tumors in GPP mice. (E) Weight of *Ptgs*$^{-/-}$ tumors four days post-implantation in *Ifng*$^{-/-}$ mice adoptively transferred with wild-type or *Ifng*$^{-/-}$ NK cells. (F) Expression levels of CI genes in *Ptgs*$^{-/-}$ tumors from *Ifng*$^{-/-}$ mice infused with either wild-type (n=5) or *Ifng*$^{-/-}$ (n=5) NK cells. Data are normalized to *Gapdh* and expressed as mean ± SEM of the fold change of the average expression in tumors from mice transferred with *Ifng*$^{-/-}$ NK cells. (G) Gene expression of factors associated with CP (red) or CI (blue) inflammation normalized to *Gapdh* in tumors from *Ptgs*$^{+/+}$ and *Ptgs*$^{-/-}$ wild-type or *Ptgs*$^{+/+}$ in GPP mice. Two-way ANOVA (A), one-way ANOVA (B, D), unpaired Student's t test (E, F).

Figure S5

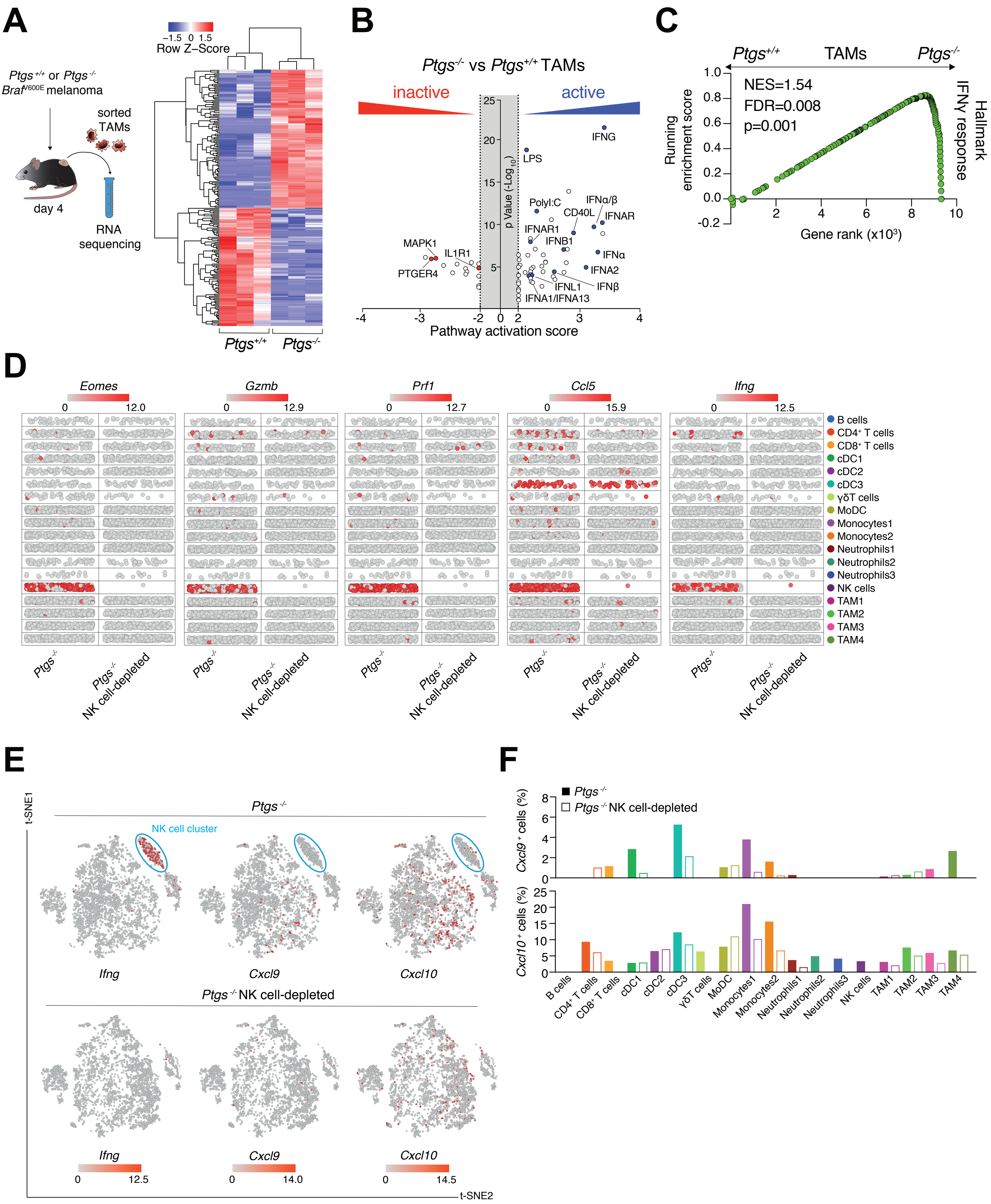

A
Ptgs+/+ or Ptgs-/-
BrafV600E melanoma
sorted TAMs
day 4
RNA sequencing
-1.5 0 1.5
Row Z-Score
Ptgs+/+
Ptgs-/-
B
Ptgs-/- vs Ptgs+/+ TAMs
inactive
active
IFNG
LPS
PolyI:C
IFNα/β
CD40L
IFNAR
IFNAR1
IFNB1
IFNα
IFNA2
IFNβ
IFNL1
IFNA1/IFNA13
MAPK1
IL1R1
PTGER4
p Value (-Log10)
Pathway activation score
C
Ptgs+/+
TAMs
Ptgs-/-
NES=1.54
FDR=0.008
p=0.001
Running enrichment score
Hallmark IFNγ response
Gene rank (x10³)
D
Eomes
Gzmb
Prf1
Ccl5
Ifng
B cells
CD4+ T cells
CD8+ T cells
cDC1
cDC2
cDC3
γδT cells
MoDC
Monocytes1
Monocytes2
Neutrophils1
Neutrophils2
Neutrophils3
NK cells
TAM1
TAM2
TAM3
TAM4
Ptgs-/-
Ptgs-/- NK cell-depleted
E
t-SNE1
t-SNE2
Ptgs-/-
NK cell cluster
Ifng
Cxcl9
Cxcl10
Ptgs-/- NK cell-depleted
F
Ptgs-/-
Ptgs-/- NK cell-depleted
Cxcl9+ cells (%)
Cxcl10+ cells (%)

Figure S5. (Related to Figure 3 and Figure 4) Bulk and single-cell RNA sequencing of tumors reveal widespread NK cell-dependent upregulation of an IFNγ-response. (A) Analysis by RNAseq of TAMs sorted from tumors formed by *Ptgs*$^{+/+}$ and *Ptgs*$^{-/-}$ melanoma cells four days post-implantation, each replicate is a pool of ten tumors. The heatmap shows z-scores for transcript levels of the differentially expressed genes (FDR≤0.05; $Log_2FC \geq 1.5$). (B) Upstream regulators predicted by Ingenuity Pathway Analysis of differentially expressed genes between TAMs isolated from *Ptgs*$^{+/+}$ versus *Ptgs*$^{-/-}$ tumors. (C) GSEA of hallmark IFNγ response gene set in TAMs from *Ptgs*$^{-/-}$ compared to *Ptgs*$^{+/+}$ tumors. FDR calculated using GSEA. (D) Dot-plot showing *Eomes*, *Gzmb*, *Prf1*, *Ccl5* and *Ifng* expression (normalized CPM) in the individual cell clusters from single cell-RNA sequencing of untreated or NK cell-depleted *Ptgs*$^{-/-}$ tumors. (E) t-SNE projections showing comparison of *Ifng*, *Cxcl9* and *Cxcl10* gene expression in the tumor immune infiltrate from *Ptgs*$^{-/-}$ and *Ptgs*$^{-/-}$ NK cell-depleted mice (F). *Cxcl9* and *Cxcl10* gene expression analysis in all cell clusters shown in Figure 4A. Data are expressed as fraction of cells expressing the indicated gene per individual cell cluster.

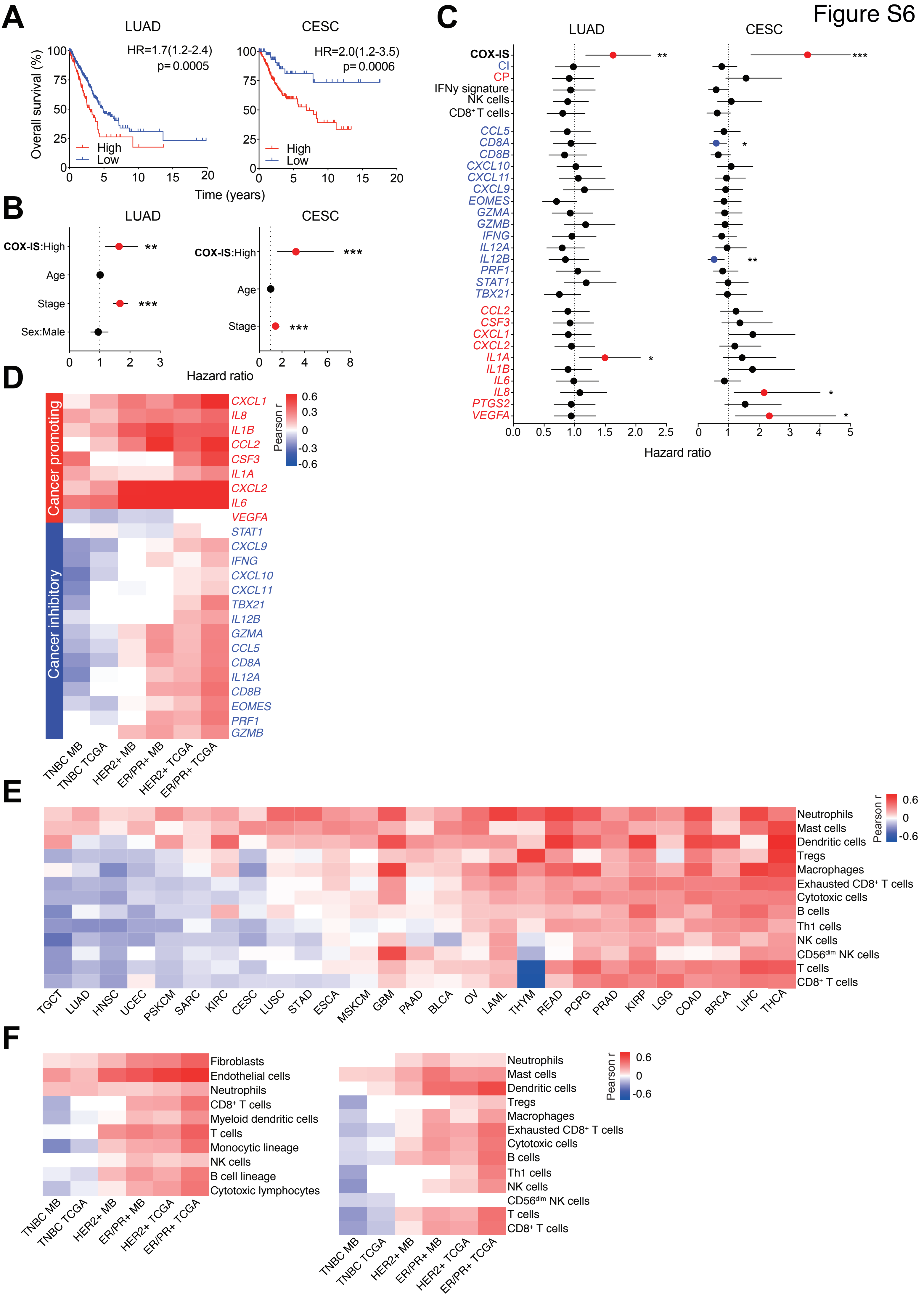
Figure S6
A
LUAD
CESC
HR=1.7(1.2-2.4)
p= 0.0005
HR=2.0(1.2-3.5)
p= 0.0006
Overall survival (%)
High
Low
Time (years)
B
COX-IS:High
Age
Stage
Sex:Male
Hazard ratio
C
COX-IS
CI
CP
IFNy signature
NK cells
CD8⁺ T cells
CCL5
CD8A
CD8B
CXCL10
CXCL11
CXCL9
EOMES
GZMA
GZMB
IFNG
IL12A
IL12B
PRF1
STAT1
TBX21
CCL2
CSF3
CXCL1
CXCL2
IL1A
IL1B
IL6
IL8
PTGS2
VEGFA
Hazard ratio
D
Cancer promoting
Cancer inhibitory
Pearson r
TNBC MB
TNBC TCGA
HER2+ MB
ER/PR+ MB
HER2+ TCGA
ER/PR+ TCGA
E
Neutrophils
Mast cells
Dendritic cells
Tregs
Macrophages
Exhausted CD8⁺ T cells
Cytotoxic cells
B cells
Th1 cells
NK cells
CD56dim NK cells
T cells
CD8⁺ T cells
TGCT LUAD HNSC UCEC PSKCM SARC KIRC CESC LUSC STAD ESCA MSKCM GBM PAAD BLCA OV LAML THYM READ PCPG PRAD KIRP LGG COAD BRCA LIHC THCA
F
Fibroblasts
Endothelial cells
Neutrophils
CD8⁺ T cells
Myeloid dendritic cells
T cells
Monocytic lineage
NK cells
B cell lineage
Cytotoxic lymphocytes

Figure S6. (Related to Figure 5 and Figure 6) COX-2 expression delineates cancer-promoting from cancer-inhibitory inflammation in human breast cancer subtypes. Survival analysis of LUAD (TCGA, n=512) and CESC (TCGA, n=305) patients stratified according to the COX-IS. (A) Kaplan-Meier survival plots parsed as high versus low COX-IS expressors at a 75% stringency. (B) Forest plots showing a multivariate Cox regression analysis for the indicated risk factors. (C) Hazard ratio associated with the indicated gene signatures or the individual gene elements of the COX-IS. Hazard ratio (95% C.I.), Log-rank (Mantel-Cox) test (A-C). (D) Heatmap showing the Pearson correlation coefficient of *PTGS2* expression with the mouse-derived COX-IS genes across the different breast cancer datasets from TCGA: TNBC (n=115), HER2+ (n=195), ER/PR+ (n=666); and METABRIC: TNBC (n=251), HER2+ (n=205), ER/PR+ (n=968). (E, F) Pearson correlation coefficients of *PTGS2* expression with the indicated cell populations as defined by Danaher et al. (E and F, right panel) or by the MCP counter algorithm (F, left panel), for all datasets shown in Figure 5A and S6D.

Figure S7

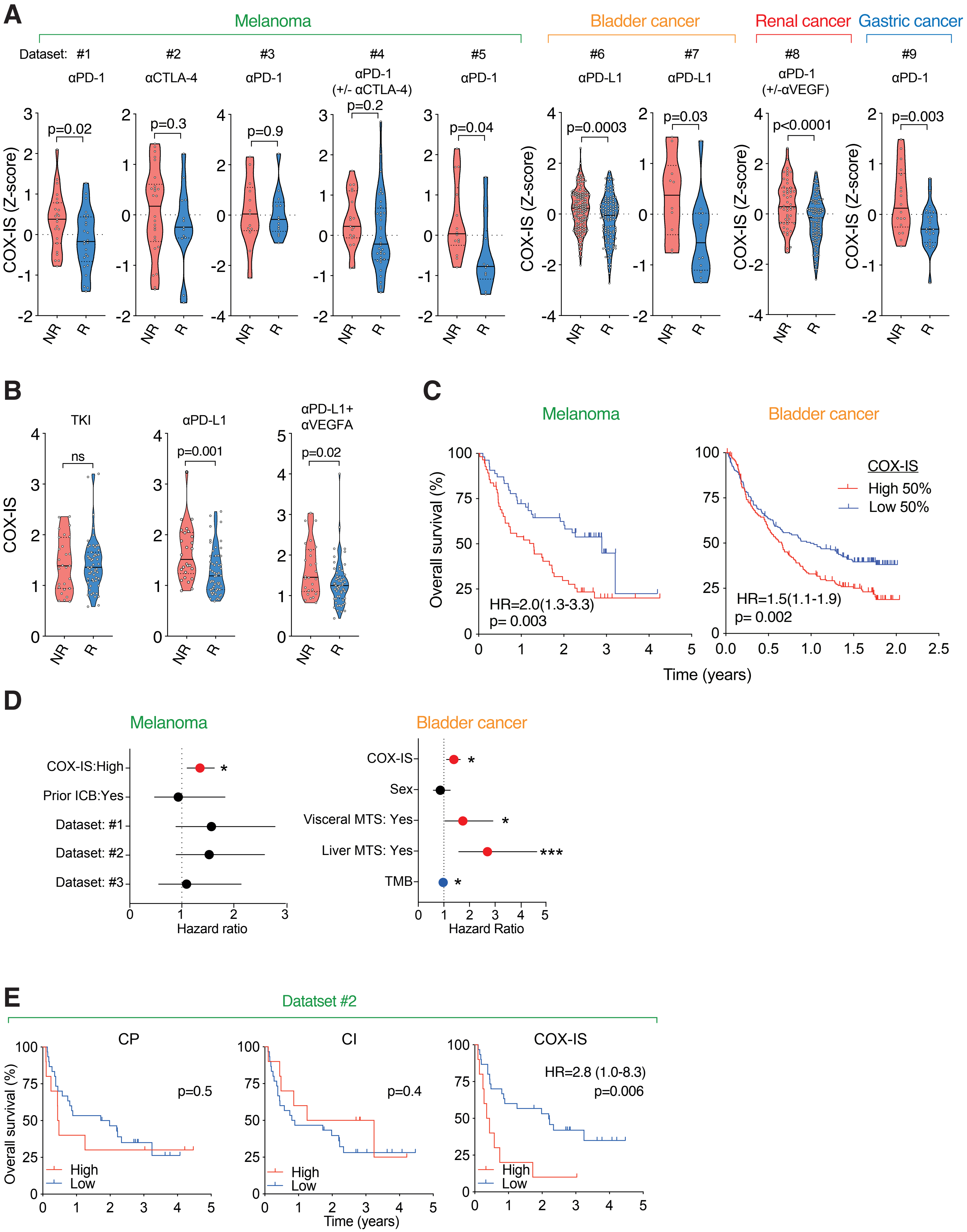

A
Melanoma
Bladder cancer
Renal cancer
Gastric cancer
Dataset: #1 #2 #3 #4 #5 #6 #7 #8 #9
αPD-1
αCTLA-4
αPD-1
αPD-1 (+/- αCTLA-4)
αPD-1
αPD-L1
αPD-L1
αPD-1 (+/-αVEGF)
αPD-1
p=0.02
p=0.3
p=0.9
p=0.2
p=0.04
p=0.0003
p=0.03
p<0.0001
p=0.003
COX-IS (Z-score)
NR
R
B
TKI
αPD-L1
αPD-L1+ αVEGFA
ns
p=0.001
p=0.02
COX-IS
C
Melanoma
Bladder cancer
Overall survival (%)
HR=2.0(1.3-3.3)
p= 0.003
HR=1.5(1.1-1.9)
p= 0.002
COX-IS
High 50%
Low 50%
Time (years)
D
Melanoma
Bladder cancer
COX-IS:High
Prior ICB:Yes
Dataset: #1
Dataset: #2
Dataset: #3
Hazard ratio
COX-IS
Sex
Visceral MTS: Yes
Liver MTS: Yes
TMB
Hazard Ratio
E
Datatset #2
CP
CI
COX-IS
p=0.5
p=0.4
HR=2.8 (1.0-8.3)
p=0.006
High
Low
Overall survival (%)
Time (years)

Figure S7. (Related to Figure 7) The COX-IS associates with outcome from ICB. (A) Analysis of COX-IS (computed from z-score normalized gene expression) at baseline in R and NR groups of patients reported in Figure 7A. (B) Analysis of COX-IS at baseline in R and NR patients from dataset #8 divided accordingly to the treatment received: TKI: sunitinib; αPD-L1: atezolizumab; αVEGF: bevacizumab. (C) Kaplan-Meier survival plots for melanoma (pooled datasets #1, #2, #3 and #4) and bladder cancer (dataset #6) parsed as high versus low on a median cut-off for baseline COX-IS. (D) Multivariate Cox regression analysis for the indicated risk factors in melanoma (pooled datasets #1, #2, #3 and #4) and bladder cancer (dataset #6) patients. (E) Survival analysis of patients from dataset #2 stratified based on the CP, CI signatures or the COX-IS.
