# Supplementary material for: Antagonistic Inflammatory Phenotypes Dictate Tumor Fate and Response to Immune Checkpoint Blockade
Source: Immunity. 2020 Dec 15;53(6):1215–1229.e8. doi: 10.1016/j.immuni.2020.10.020 (PMC7772804; doi:10.1016/j.immuni.2020.10.020)
Supplement: Document S2. Article plus Supplemental Information [file mmc7.pdf]

Article
Immunity

# Antagonistic Inflammatory Phenotypes Dictate Tumor Fate and Response to Immune Checkpoint Blockade

## Graphical Abstract

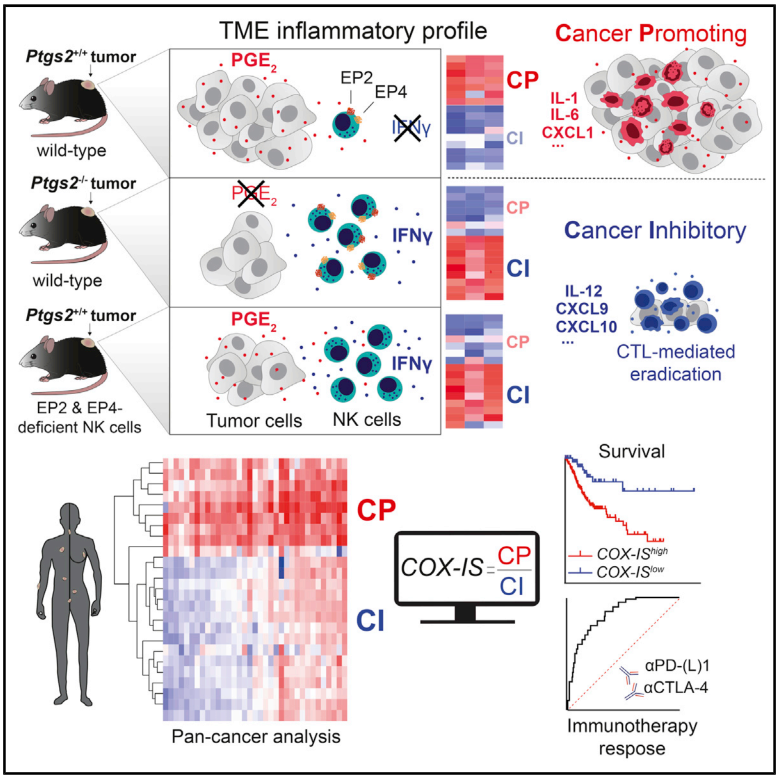


## Highlights

- NK cells drive broad inflammatory remodeling characteristic of T-cell-inflamed tumors
- PGE2 acting on EP2 and EP4 on NK cells prevents the TME switch enabling immune escape
- Opposing inflammatory profiles found in many human cancer types have prognostic value
- A signature capturing pro- and anti-tumor factors predicts response to immunotherapy

## Authors

Eduardo Bonavita, Christian P. Bromley, Gustav Jonsson, ..., Nadia Guerra, Daniel M. Davis, Santiago Zelenay

## Correspondence

santiago.zelenay@cruk.manchester.ac.uk

## In Brief

Inflammation can fuel or suppress cancer. Bonavita et al. identify NK cells as primary orchestrators of T cell inflammation in murine models and devise an inflammatory signature that predicts patient survival and response to immunotherapy in multiple malignancies.

Bonavita et al., 2020, Immunity *53*, 1215–1229
December 15, 2020 © 2020 The Author(s). Published by Elsevier Inc.
https://doi.org/10.1016/j.immuni.2020.10.020

Immunity

CellPress OPEN ACCESS

Article

# Antagonistic Inflammatory Phenotypes Dictate Tumor Fate and Response to Immune Checkpoint Blockade

Eduardo Bonavita,[1] Christian P. Bromley,[1] Gustav Jonsson,[2] Victoria S. Pelly,[1] Sudhakar Sahoo,[1] Katherine Walwyn-Brown,[3] Sofia Mensurado,[4] Agrin Moeini,[1] Eimear Flanagan,[1] Charlotte R. Bell,[1] Shih-Chieh Chiang,[1] C.P. Chikkanna-Gowda,[1] Neil Rogers,[5] Bruno Silva-Santos,[4] Sebastien Jaillon,[6] Alberto Mantovani,[6] Caetano Reis e Sousa,[5] Nadia Guerra,[2] Daniel M. Davis,[3] and Santiago Zelenay[1,7,*]
[1]Cancer Research UK Manchester Institute, The University of Manchester, Alderley Park, UK
[2]Department of Life Sciences, Imperial College London, London, UK
[3]Manchester Collaborative Centre for Inflammation Research, The Lydia Becker Institute of Immunology and Inflammation, The University of Manchester, Manchester, UK
[4]Instituto de Medicina Molecular João Lobo Antunes, Faculdade de Medicina, Universidade de Lisboa, Lisbon, Portugal
[5]The Francis Crick Institute, London, UK
[6]Humanitas University, Department of Biomedical Sciences, Humanitas Clinical and Research Center, IRCCS, Milan, Italy
[7]Lead Contact
*Correspondence: santiago.zelenay@cruk.manchester.ac.uk
https://doi.org/10.1016/j.immuni.2020.10.020

## SUMMARY

Inflammation can support or restrain cancer progression and the response to therapy. Here, we searched for primary regulators of cancer-inhibitory inflammation through deep profiling of inflammatory tumor microenvironments (TMEs) linked to immune-dependent control in mice. We found that early intratumoral accumulation of interferon gamma (IFN-γ)-producing natural killer (NK) cells induced a profound remodeling of the TME and unleashed cytotoxic T cell (CTL)-mediated tumor eradication. Mechanistically, tumor-derived prostaglandin E2 (PGE2) acted selectively on EP2 and EP4 receptors on NK cells, hampered the TME switch, and enabled immune evasion. Analysis of patient datasets across human cancers revealed distinct inflammatory TME phenotypes resembling those associated with cancer immune control versus escape in mice. This allowed us to generate a gene-expression signature that integrated opposing inflammatory factors and predicted patient survival and response to immune checkpoint blockade. Our findings identify features of the tumor inflammatory milieu associated with immune control of cancer and establish a strategy to predict immunotherapy outcomes.

## INTRODUCTION

The concept that cancer induces inflammation and that inflammatory cells within the tumor bed can support cancer progression is well established (Coussens et al., 2013; Hanahan and Weinberg, 2011; Mantovani et al., 2008). Aggressive and invasive tumor behaviors in both preclinical models and patients are commonly associated with tumor-infiltrating macrophages, neutrophils, immature myeloid cells, or regulatory T cells and with molecules produced by these and other leukocytes, as well as stromal cells, or cancer cells themselves. Interleukin-6 (IL-6), IL-8, transforming growth factor β (TGF-β), CXCL1, or VEGF are examples of soluble factors with pleiotropic effects that can foster cancer growth and spread (Coussens et al., 2013; Mantovani et al., 2008).

Inflammation at the tumor site can also have a protective role, partly by contributing to immune recognition and elimination of cancer cells. Cytotoxic T cells (CTLs), in particular, are major anti-tumor effectors in preclinical cancer models, and their intratumoral abundance is associated with improved patient outcome and response to cancer therapy (Binnewies et al., 2018; Fridman et al., 2012). Accordingly, high intratumoral levels of CTL chemoattractants, such as CXCL9 or CXCL10, or cytokines that promote CTL differentiation and effector function, such as IL-12 or type I and II interferons (IFNs), have also been linked with a favorable prognosis (Chow et al., 2019; Dangaj et al., 2019; Spranger et al., 2017; Vesely et al., 2011). In addition to conventional helper $CD4^+$ and cytotoxic $CD8^+$ T cells, other immune subsets, such as natural killer (NK) cells, γδ T cells, or the Batf3-dependent conventional type I dendritic cells (cDC1) have also been associated with improved outcome (Barry et al., 2018; Böttcher et al., 2018; Gentles et al., 2015; Morvan and Lanier, 2016; Salmon et al., 2016; Spranger et al., 2017). This is true both


This is an open access article under the CC BY license (http://creativecommons.org/licenses/by/4.0/).

in spontaneous and in therapy-induced anti-tumor responses, such as those ensuing from the administration of immune checkpoint inhibitors.

Particularly in immune checkpoint blockade (ICB) therapy, preexisting intratumoral T cell immunity ( Herbst et al., 2014; Tumeh et al., 2014) and activation of specific inflammatory pathways (Ayers et al., 2017; Cristescu et al., 2018; Rooney et al., 2015) have been associated with treatment efficacy. Objective responses from ICB are generally greater in the so-called "hot" T cell-inflamed tumors characterized by higher CTL infiltration, tumor mutational burden (TMB), neoantigen load, and clonality or IFN-$\gamma$ signaling (Ayers et al., 2017; Cristescu et al., 2018; McGranahan et al., 2016; Rizvi et al., 2015; Snyder et al., 2014; Van Allen et al., 2015). However, the prognostic and predictive utility of these tumor features is limited and varies across malignancies. Indeed, patients with equally high TMB or CD8 T cell abundance experienced dichotomous responses to ICB (Rizvi et al., 2015; Snyder et al., 2014; Van Allen et al., 2015).

Despite their pivotal role in cancer progression and response to therapy, mechanisms that dictate the balance between cancer-promoting versus cancer-inhibitory inflammation within the tumor microenvironment (TME) are not clear (Shalapour and Karin, 2019). In particular, the underlying signals and pathways that instruct the establishment of T cell-inflamed tumors are not completely understood. Although high TMB or evidence of IFN-$\gamma$ signaling help discriminate hot from "cold" tumors, at least in some cancer types, what precedes and drives T cell infiltration and effector function is poorly defined.

Here, we sought to identify instructive signaling pathways and factors responsible for the establishment of inflammatory TMEs characteristic of T cell-inflamed tumors. We carried out deep cellular and molecular tumor phenotyping of murine cancer models in which prostaglandin E2 (PGE2) release or lack thereof determines progression versus immune-mediated rejection, respectively (Zelenay et al., 2015). These models allowed us to study primary determinants of cancer immunogenicity and T cell control in settings of equivalent TMB but distinct inflammatory profiles coupled to dichotomous tumor fates. We found that the control of tumors deficient in cyclooxygenase enzymes (COXs), and rate limiting for PGE2 synthesis, was independent of major innate immune signaling pathways but required the early intratumoral accumulation of NK cells. As well as directly killing cancer cells, NK cell-derived IFN-$\gamma$ induced a profound molecular remodeling of the TME, ultimately leading to CTL-mediated tumor eradication. Mechanistically, PGE2 acted selectively on NK cells by EP2 and EP4 receptors to inhibit the TME switch and thereby enabling immune escape. To assess the potential translational relevance of our findings, we analyzed large cancer patient datasets, including multiple independent cohorts of patients receiving ICB. We showed that pro- and anti-tumorigenic inflammatory molecular profiles associated with the COX-2/PGE2 axis and NK cell activity, respectively, can be found within many human cancers and have independent prognostic utility. Critically, a murine-derived gene signature that integrated these antagonistic inflammatory phenotypes predicted ICB outcome in multiple human tumor types, even in patient cohorts in which established immune biomarkers failed to do so.

## RESULTS

### Increased NK Cell and Reduced Neutrophil Accumulation Precede Adaptive Immune Control of COX-Deficient Tumors

To search for orchestrators of hot TMEs, we exploited an experimental system in which divergent immune-dependent tumor fates are consistently observed independently of the overall TMB of cancer cells. In this model, tumors are rendered spontaneously immunogenic by cancer cell-intrinsic ablation of the COX-2/PGE2 synthesis pathway. Thus, *Braf*$^{V600E}$-driven melanoma cells made deficient for COX-1 and -2 (*Ptgs*$^{-/-}$ cells) fail to form progressive tumors in immunocompetent mice, but their COX-competent parental counterpart (*Ptgs*$^{+/+}$) subverts adaptive immunity and grows uncontrollably (Zelenay et al., 2015). To confirm that these opposing outcomes were fully attributable to differences in cancer cell COX competence, we restored COX activity in *Ptgs*$^{-/-}$ cells by retroviral transduction. COX-2-complementation re-established PGE2 production by mutant *Ptgs*$^{-/-}$ cells and their ability to form progressive tumors in immunocompetent mice (Figures S1A and S1B).

To investigate the basis for immune-mediated control in this model, we first assessed the involvement of canonical innate immune signaling pathways that classically initiate and coordinate innate and adaptive immunity. Notably, *Ptgs*$^{-/-}$ tumors still regressed in mice deficient in the signal transducers MyD88, Toll-IL-1R resistance domain-containing adapter-inducing IFN-$\beta$ (TRIF), mitochondrial antiviral signaling protein (MAVS), cyclic GMP-AMP synthase (cGAS), or stimulator of IFN genes (STING) with similar kinetics to wild-type hosts (Figure 1A), implying that TLR, RLR, and cGAS pathways are all redundant for immune-mediated control of these tumors. We next characterized the tumor-infiltrating immune cell composition early on to understand what initially facilitates tumor control. COX-deficient tumors were noticeably smaller than parental or COX-2-restored cells 4 days following implantation (Figure S1C), before the onset of adaptive immunity, which is apparent only 7 to 10 days post-implantation (Zelenay et al., 2015). Examination of multiple immune cell types by flow cytometry showed an increase in neutrophils and a marked reduction in NK cell infiltration in COX-sufficient tumors (Figures 1B–1D and S1H). Other leukocyte populations were unchanged or less consistently affected at this time point (Figures 1B, S1D, S1E, and S1H). Kinetics of immune cell composition revealed a divergence in the accumulation of neutrophils and NK cells from as early as 2 days post-implantation (Figure S1F). This phenomenon was also observed in large progressive *Ptgs*$^{+/+}$ and *Ptgs*$^{-/-}$ tumors 1 month post-implantation in *Rag1*$^{-/-}$ mice (Figure S1G). An analysis of tumor sections by immunofluorescence further validated the absence of Ly6G$^{+}$ cells and the presence of NK1.1$^{+}$ cell clusters in COX-deficient tumors (Figures S1I and S1J).

We extended our analysis to other widely used cancer models with varying immunogenic potentials. In colorectal MC38 and CT26 and breast 4T1 cells, ablation of the COX-2/PGE2 pathway significantly impaired tumor growth selectively in immunocompetent hosts (Figures S2A and S2B; Zelenay et al., 2015). As in the *Braf*$^{V600E}$-driven melanoma model, their early leukocyte infiltrate showed decreased neutrophil and increased NK cell accumulation across the different COX-deficient tumor models,

CellPress
OPEN ACCESS

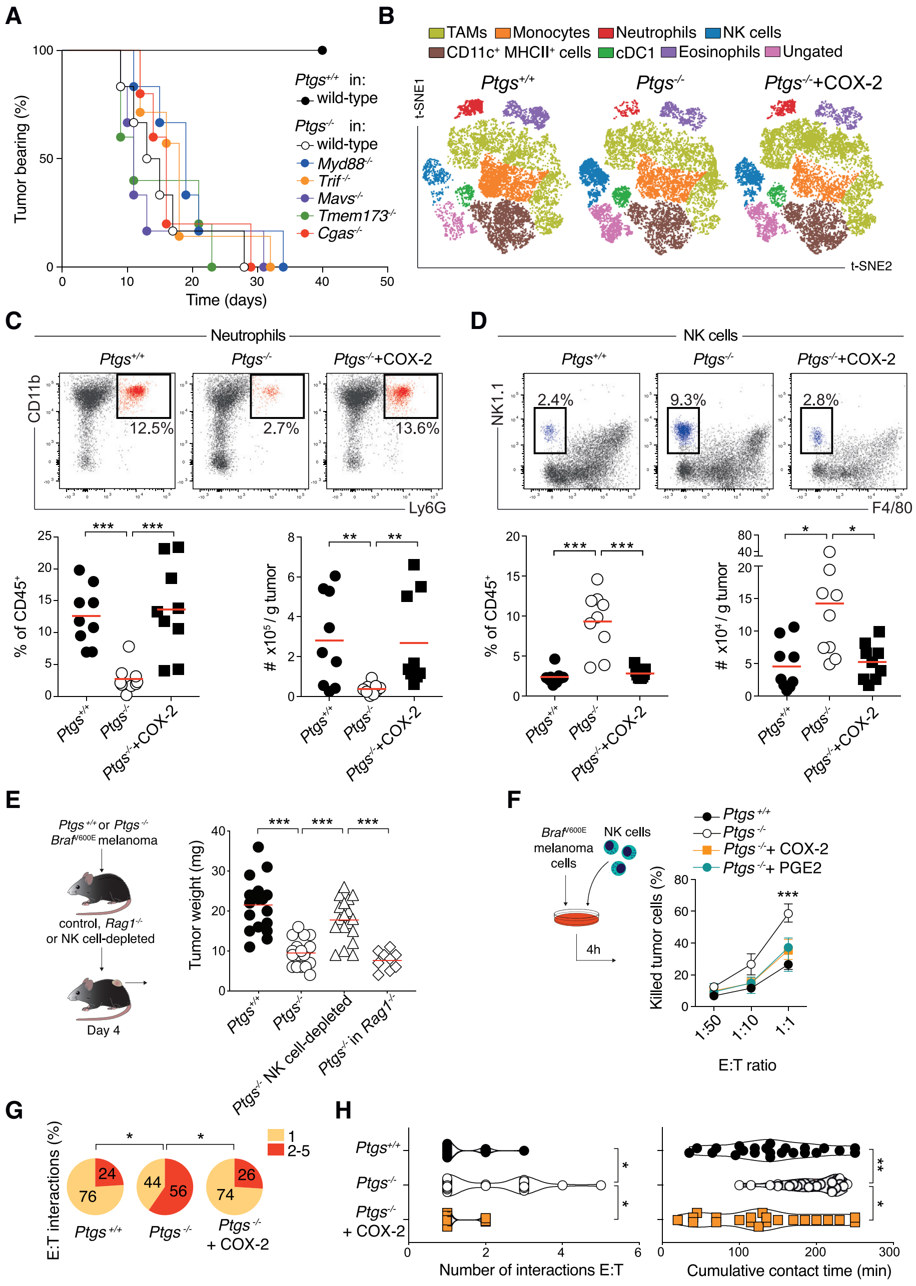


**Figure 1. Ablation of Cancer Cell-Intrinsic COX Alters the Intratumoral Accumulation of Select Innate Immune Cell Subsets**

(A) Kaplan-Meier plots showing the fraction of tumor-bearing wild-type (n = 10), *Tmem173*$^{-/-}$ (n = 5), *Cgas*$^{-/-}$ (n = 5), *Trif*$^{-/-}$ (n = 7), *Mavs*$^{-/-}$ (n = 6), and *Myd88*$^{-/-}$ (n = 6) mice injected with *Ptgs*$^{-/-}$ or *Ptgs*$^{+/+}$ melanoma tumor cells in wild-type (n = 10) hosts.

(B–D) Immune cell infiltrate analysis of *Ptgs*$^{+/+}$, *Ptgs*$^{-/-}$, and *Ptgs*$^{-/-}$+COX-2 tumors 4 days post-implantation. (B) Two-dimensional distributed stochastic neighbor embedding (t-SNE) projections of CD45$^{+}$ cells for each group (n = 6 concatenated samples per group). The frequency and the number of intratumoral neutrophils (C) and NK cells (D) are shown.

(E) Weight of *Ptgs*$^{+/+}$ and *Ptgs*$^{-/-}$ tumors 4 days post-implantation in wild-type, NK cell-depleted, or *Rag1*$^{-/-}$ mice.

(F) *Ptgs*$^{+/+}$, *Ptgs*$^{-/-}$ (+/− synthetic PGE2), and *Ptgs*$^{-/-}$+COX-2 melanoma cells tested for susceptibility to NK cell-mediated killing. E:T refers to ratio of effector:target cells.

(G) Percentage of NK cells contacting either 1 or more (2–5) *Ptgs*$^{+/+}$, *Ptgs*$^{-/-}$, or *Ptgs*$^{-/-}$+COX2 targets.

(H) Violin plots representing the number of interactions and the cumulative contact time of NK cells with *Ptgs*$^{+/+}$, *Ptgs*$^{-/-}$, and *Ptgs*$^{-/-}$+COX-2 targets. Data are expressed as mean ± SEM, one-way ANOVA (C–F and H) or Fisher's exact test. (G).

whereas other innate immune cell subsets, including monocytes, tumor-associated macrophages (TAMs), or DCs were largely unaffected. This opposing pattern of neutrophil and NK cell infiltration was also observed comparing early and established *Ptgs*$^{+/+}$ and *Ptgs*$^{-/-}$ 4T1 tumors implanted orthotopically into the mammary fat-pad (Figure S2D). Altogether, these data indicated that cancer cell-intrinsic COX-2 activity attracts neutrophils and hinders early intratumoral NK cell buildup across several cancer models.

## NK Cells Are Essential for Both T Cell-Independent and T Cell-Dependent Tumor Control

To assess the contribution of either neutrophils and/or NK cells as putative early regulators of the TME composition and tumor fate, we monitored tumor burden and analyzed the immune cell infiltrate following their antibody-mediated depletion. Elimination of neutrophils and/or other GR-1$^{+}$ cells altered neither tumor size nor the prevalence of other leukocyte subsets in COX-competent or -deficient tumors (see Mendeley Data). In contrast, NK cell depletion led to a clear increase in *Ptgs*$^{-/-}$ tumor size and weight, which became comparable to those of COX-competent tumors as early as 4 days after cancer cell implantation (Figure 1E). Of note, despite the significant increase in *Ptgs*$^{-/-}$ tumor size, the overall content of other innate immune cell subsets was not grossly altered at this time point, neither in the melanoma nor MC38 colorectal models (Figures S3A and S3B).

Early control of *Ptgs*$^{-/-}$ tumors was still noticeable in *Rag1*$^{-/-}$ mice lacking adaptive immunity (Figure 1E), arguing for direct NK cell cytotoxic activity against *Ptgs*$^{-/-}$ tumor cells. Indeed, NK cells were more efficient at killing COX-deficient than parental or COX-2-restored melanoma cells *in vitro* or COX-deficient cells in the presence of synthetic PGE2 (Figure 1F). Furthermore, live imaging of NK and cancer cell co-cultures showed that COX-2 activity hindered the interaction of NK cells with tumor cells (Figures 1G and 1H). Migration tracking of NK cells showed that the frequency of contacts with more than one target and the overall cumulative target contact time were significantly higher in co-cultures of NK cells with *Ptgs*$^{-/-}$ tumor cells (Figures 1G and 1H). NK cell viability or migratory behavior were, conversely, not altered by cancer cell COX-2 sufficiency in these experimental settings (Figures S3D, S3E, and S3F). Overall, these data supported a model whereby NK cells directly kill COX-deficient cancer cells, restricting early tumor growth.

Sustained and long-term growth control of *Ptgs*$^{-/-}$ melanoma and MC38 colorectal tumors was also impaired in NK cell-depleted mice in which tumors grew as progressively as their parental counterparts (Figures 2A, S3C, and S3G). Yet, this prolonged growth restriction was equally dependent on adaptive immunity (Figures 2A, S2B, and S3G), indicating that sole NK cell cytotoxic activity is insufficient for tumor eradication. Therefore, to determine the relative and hierarchical involvement of different lymphocyte subsets in tumor control, we compared the growth of *Ptgs*$^{-/-}$ tumors in *Rag1*$^{-/-}$ mice or in wild-type mice depleted of either NK cells, CD4$^{+}$ T cells, CD8$^{+}$ T cells, or both CD4$^{+}$ and CD8$^{+}$ T cells. In agreement with NK cell participation in both innate and adaptive phases of immune control, tumors grew faster in NK cell-depleted mice than in mice lacking only CD8$^{+}$ T cells (Figures 2A and S3G) and even more aggressively in hosts lacking both NK cells and T cells (Figure S3H). Nevertheless, tumors progressed, and full eradications were never observed in NK cell-competent *Rag1*$^{-/-}$ hosts or following depletion of CD8$^{+}$ T cells (Figures 2A, S3G, S3H, and S3I). Last, tumors were still rejected in mice depleted of CD4$^{+}$ T cells or lacking $\gamma\delta$ T cells (Figures 2A, S3G, and S3I). However, combined ablation of both CD4$^{+}$ and CD8$^{+}$ cells led to faster tumor growth than in mice ablated of just CD8$^{+}$ T cells, suggesting a non-redundant contribution of CD4$^{+}$ T cells (Figures 2A and S3G). Taken together, these data indicated that among different lymphocyte subsets, NK cells were uniquely responsible for early control; however, full tumor eradication relied on the action of both NK and conventional T cells.

In agreement with the hypothesis that NK cells were upstream of adaptive immunity, their early depletion inhibited the accumulation of CD8$^{+}$ T cells within *Ptgs*$^{-/-}$ tumors evaluated 7 days after tumor cell implantation (Figure 2B). Intratumoral activation and effector T cell function were also markedly compromised in the absence of NK cells, as revealed by the enumeration of tumor-infiltrating CD44$^{+}$ CD8$^{+}$ T cells and IFN-$\gamma$-producing CD8$^{+}$ or CD4$^{+}$ T cells (Figure 2C). Notably, delaying NK cell ablation to this time point did not impair the eradication of *Ptgs*$^{-/-}$ tumors (Figure 2D), ascribing a primary early role for NK cells in setting the stage for the ensuing T cell-mediated tumor elimination.

## Genetic Ablation of PGE2 Receptors EP2 and EP4 on NK Cells Unleashes T Cell Immunity toward COX-Competent Tumors

To further examine the early instructive role of NK cells in the initiation of cancer immunity and to validate our findings in a setting independent of CRISPR mutant cells, we took advantage of genetically engineered mice lacking EP2 and EP4 receptors (encoded by *Ptger2* and *Ptger4*), which mediate the downstream immunomodulatory signaling of PGE2 (Kalinski, 2012). In *Gzmb*-Cre *Ptger4*$^{floxed/floxed}$ *Ptger2*$^{-/-}$ mice (referred to as GPP mice) (Chen et al., 2015) all cells are deficient for EP2 but only granzyme-B-expressing cells lack both EP2 and EP4. Strikingly, parental COX-competent melanoma cells failed to form progressive tumors when implanted into GPP mice, mirroring the growth profile phenotype of their COX-deficient counterparts in wild-type hosts (Figure 2E). Equally, parental *Ptgs2*$^{+/+}$ MC38 colorectal cells showed evidence of delayed tumor growth in GPP mice (Figure S4A). *Ptgs*$^{+/+}$ melanoma cells grew progressively in either single germline EP2-deficient or conditional EP4-deficient mice, suggesting the dual deletion of both PGE2 receptors on granzyme B$^{+}$ cells was required for spontaneous tumor regression (Figure 2G). In the spleen and early on among tumor-infiltrating immune cells, NK cells constituted the vast majority of granzyme B$^{+}$ cells, with only a very small proportion of CD3$^{+}$ T cells expressing granzyme B, as determined by fluorescence-activated cell sorting (FACS) (Figure S4C). Indeed, consistent with NK cells being the primary and direct cellular target of cancer cell-derived PGE2 *in vivo*, *Ptgs*$^{+/+}$ tumors in GPP mice were smaller early on and showed heightened infiltration by NK cells and CTLs at days 4 and 7, respectively (Figures 2F and S4B). These tumors also had significantly fewer neutrophils, fully recapitulating the phenotype of *Ptgs*$^{-/-}$ tumors in wild-type animals (Figure S4D) and implying an essential suppressive role for both EP2 and EP4 on NK cells in preventing tumor-eradicating immunity.

CellPress
OPEN ACCESS

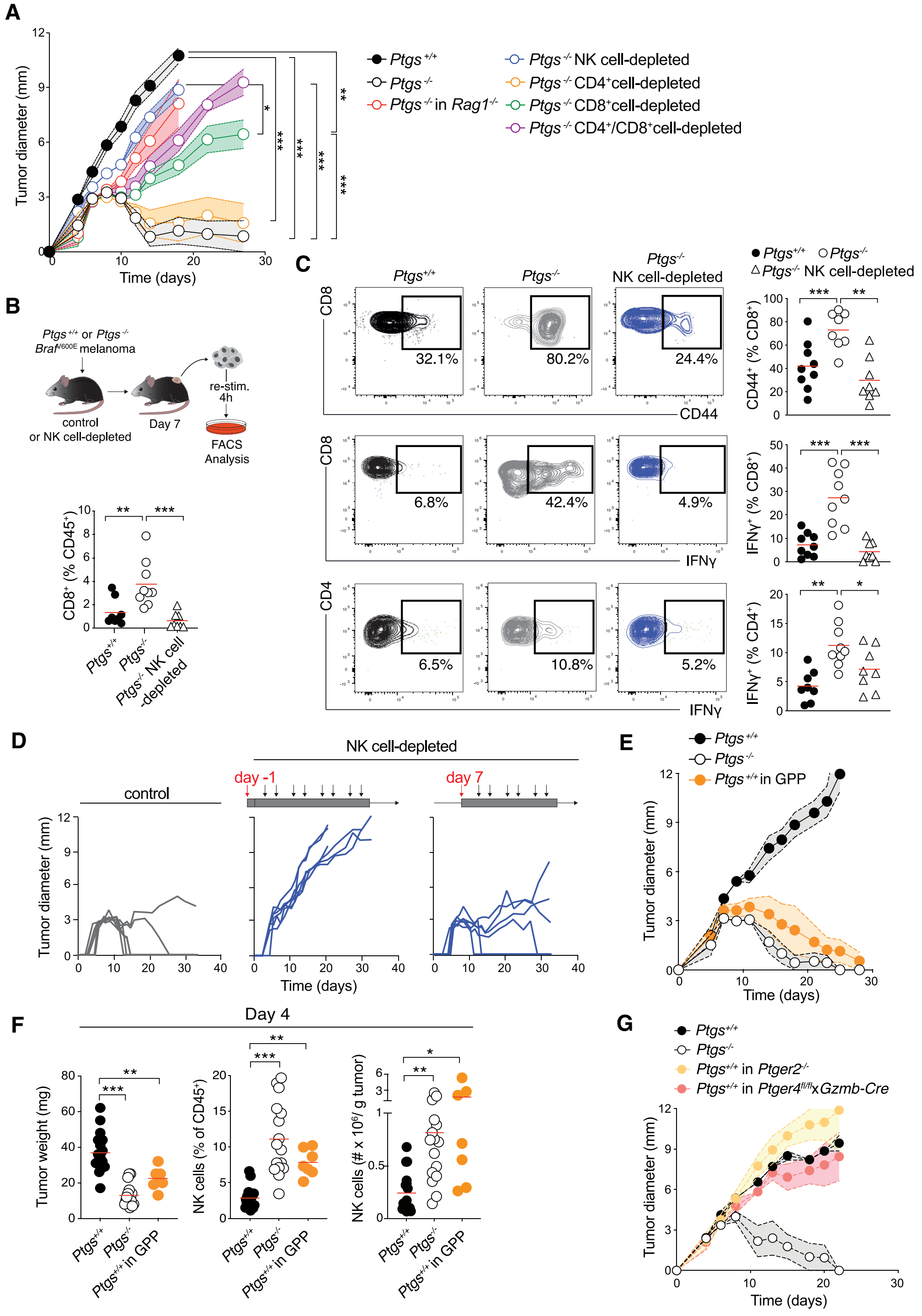


**Figure 2. NK Cells Contribute to Both Innate and Adaptive Immune Control of *Ptgs*$^{-/-}$ Tumors**

(A) Growth profile of *Ptgs*$^{+/+}$ and *Ptgs*$^{-/-}$ melanoma cells implanted into *Rag1*$^{-/-}$ or wild-type mice untreated or depleted of NK, CD4$^{+}$, and/or CD8$^{+}$ cells.

(B and C) Frequency of CD8$^{+}$ T cells (B); representative plots and frequency of CD8$^{+}$ CD44$^{+}$, CD8$^{+}$ IFN$\gamma^{+}$, and CD4$^{+}$ IFN$\gamma^{+}$ T cells (C) gated on live, CD45$^{+}$, CD3$\varepsilon^{+}$ cells in *Ptgs*$^{+/+}$ and *Ptgs*$^{-/-}$ untreated or NK cell-depleted wild-type mice analyzed 7 days post-implantation.

(D) Individual growth profiles of *Ptgs*$^{-/-}$ melanoma cells in wild-type mice depleted of NK cells from the day before or a week after cancer cell implantation.

(E) Growth profile of *Ptgs*$^{+/+}$ and *Ptgs*$^{-/-}$ cells in wild-type mice or *Ptgs*$^{+/+}$ cells in GPP mice.

(F) Tumor weight, NK cell frequency, and number per g of tumor analyzed 4 days after implantation of *Ptgs*$^{+/+}$ and *Ptgs*$^{-/-}$ cells in wild-type or *Ptgs*$^{+/+}$ cells in GPP mice.

(G) Growth profile of *Ptgs*$^{+/+}$ and *Ptgs*$^{-/-}$ melanoma cells in wild-type or *Ptgs*$^{+/+}$ melanoma cells in *Ptger2*$^{-/-}$ or *Gzmb-Cre Ptger4*$^{floxed/floxed}$ mice. Data are expressed as mean ± SEM, one-way (B–C and F) or two-way ANOVA (A).

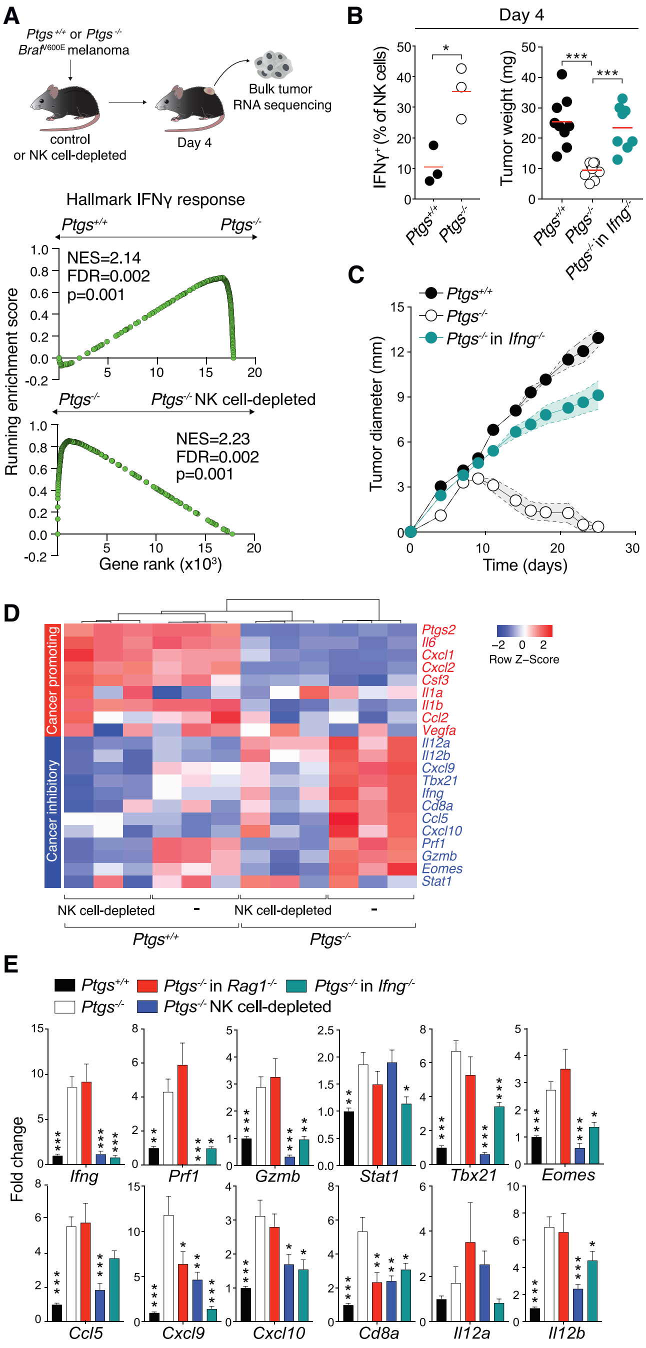


**Figure 3. IFNγ-Producing NK Cells Drive an Early Switch toward Cancer Inhibitory Inflammation Characteristic of T Cell-Inflamed Tumors**

(A) Analysis by RNA sequencing (RNA-seq) of bulk *Ptgs*$^{+/+}$ and *Ptgs*$^{-/-}$ tumors in wild-type mice or *Ptgs*$^{-/-}$ tumors from NK cell-depleted mice 4 days post-implantation. GSEA of a hallmark IFN-γ response gene set in *Ptgs*$^{-/-}$ tumors compared to that of *Ptgs*$^{+/+}$ or *Ptgs*$^{-/-}$ tumors from NK cell-depleted mice. False discovery rate (FDR) was calculated using GSEA.

(B) Percentage of intratumoral IFN-γ$^{+}$ NK cells and tumor weight of *Ptgs*$^{+/+}$and *Ptgs*$^{-/-}$ tumors in wild-type mice or *Ptgs*$^{-/-}$ in *Ifng*$^{-/-}$ mice 4 days post-implantation.

(C) Tumor growth of *Ptgs*$^{+/+}$and *Ptgs*$^{-/-}$ tumors in wild-type mice or *Ptgs*$^{-/-}$ in *Ifng*$^{-/-}$ mice.

(D) Gene expression of factors associated with CP (red) or CI (blue) inflammation normalized to *Gapdh* in NK cell-competent or -depleted mice injected with *Ptgs*$^{+/+}$ or *Ptgs*$^{-/-}$ melanoma cells.

(E) Expression levels of CI genes in *Ptgs*$^{+/+}$ tumors in wild-type mice (n = 16) or in *Ptgs*$^{-/-}$ tumors in NK cell-competent (n = 16) or -depleted (n = 9) wild-type, *Rag1*$^{-/-}$ (n = 9), and *Ifng*$^{-/-}$ (n = 8) mice. Data are normalized to *Gapdh* and expressed as mean ± SEM of the fold change of the average expression in *Ptgs*$^{+/+}$ tumors. One-way ANOVA followed by multiple comparisons (Dunnet) against levels in *Ptgs*$^{-/-}$ tumors from wild-type mice.

## NK Cells Drive an Early TME Molecular Reprogramming Characteristic of T Cell-Inflamed Tumors through IFN-γ Production

To investigate the mechanistic basis for the contribution of NK cells to early changes in the TME, we profiled the transcriptome of TAMs, the most abundant immune cell subset within both COX-competent and -deficient tumors by RNA sequencing (Figures 1B, S1E, and S5A). Pathway analysis revealed downregulation of processes associated with prostaglandin and IL-1 signaling and upregulation of type I and type II IFN pathways in TAMs isolated from *Ptgs*$^{-/-}$ tumors (Figure S5B). Gene set enrichment analysis (GSEA) equally uncovered a marked enrichment in "Hallmark IFN-γ response" (Figure S5C). This switch in the molecular profile of TAMs toward IFN-γ signaling was also evident by bulk tumor RNA sequencing and, crucially, depended on NK cell presence (Figure 3A). These data suggested that early accumulation of NK cells is essential for the polarization of COX-deficient tumors toward cancer-inhibitory (CI) inflammation and that IFN-γ may play a role in this process. Indeed, *Ptgs*$^{-/-}$ tumors were enriched in IFN-γ-producing NK cells and, when implanted into IFN-γ-deficient mice, had comparable weight to their parental counterparts early on and grew progressively over time (Figures 3B and 3C). Moreover, wild-type NK cells were superior to *Ifng*$^{-/-}$ NK cells at controlling tumor growth following their adoptive transfer to *Ptgs*$^{-/-}$ tumor-bearing *Ifng*$^{-/-}$ mice (Figure S4F).

The potential involvement of NK cells in the early intratumoral inflammatory switch was further confirmed by monitoring the expression of hallmark pro- and anti-tumorigenic factors in mice bearing *Ptgs*$^{+/+}$ or *Ptgs*$^{-/-}$ tumors depleted of NK cells. Transcript levels of soluble factors often linked to cancer-promoting (CP) inflammation, such as IL-6, CXCL1, CXCL2, IL-1β, or G-CSF, were markedly higher in COX-competent compared to COX-deficient tumors, and their expression was unchanged in the absence of NK cells (Figure 3D). In contrast, expression of CI factors, such as IFN-γ, CXCL9, CXCL10, T-bet, or granzyme B, was significantly reduced following NK cell depletion (Figures 3D and 3E). This effect was particularly evident in *Ptgs*$^{-/-}$ tumors in which the expression of these genes was highest.

Upregulation of CI inflammatory factors depended on NK cells but was largely unaltered in *Rag1*$^{-/-}$ hosts, lacking αβ and γδ T cells, NK T cells and B cells (Figure 3E). Notably, IFN-γ deficiency phenocopied the effects of NK cell depletion, as levels of CI transcripts were significantly reduced in *Ptgs*$^{-/-}$ tumors implanted in *Ifng*$^{-/-}$ mice (Figure 3E). Moreover, adoptive transfer of wild-type IFN-γ-competent NK cells into these mice reverted the defect, raising the expression of CI mediators by 4-fold on average compared to control *Ifng*$^{-/-}$ mice receiving

CellPress
OPEN ACCESS

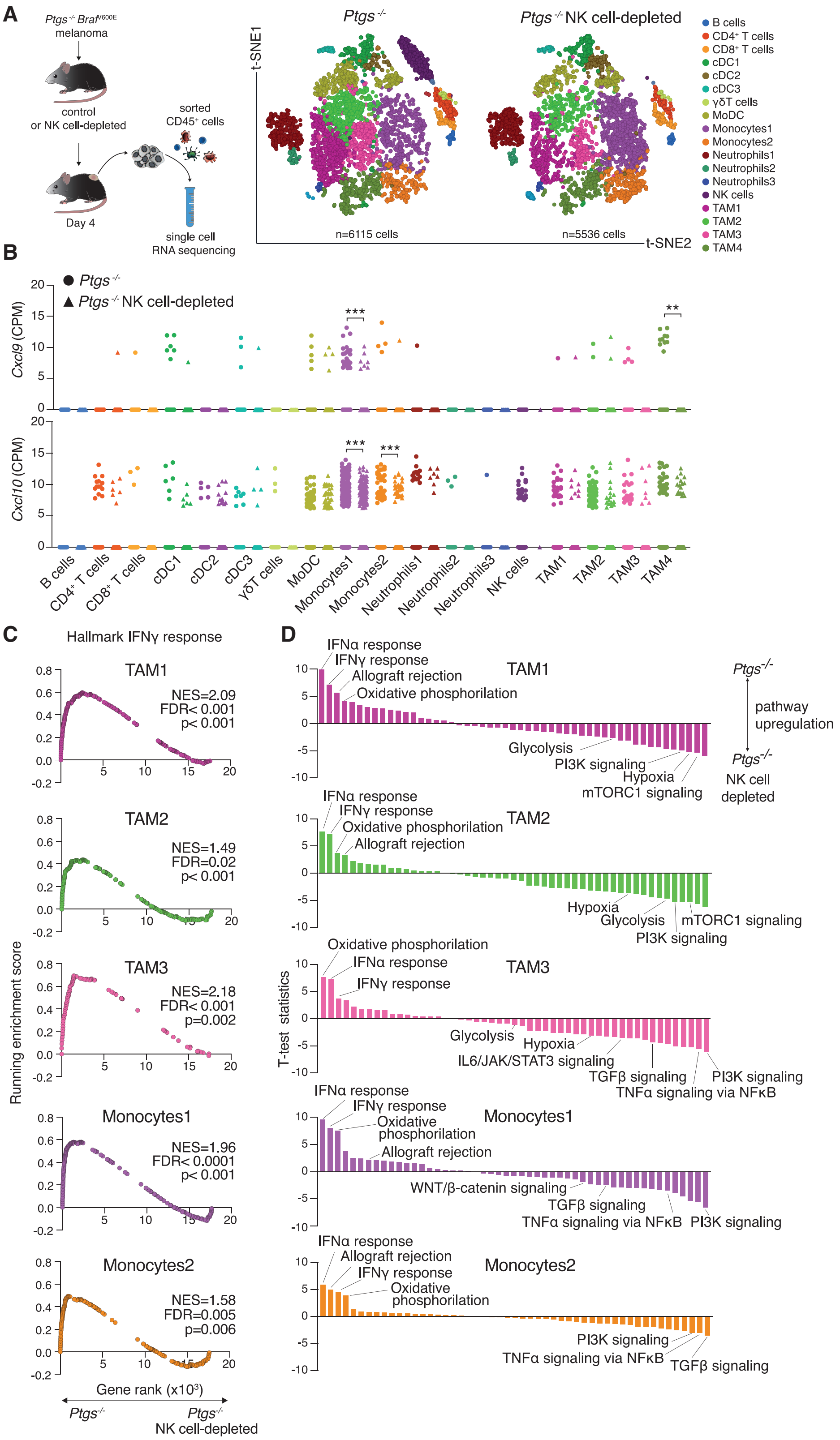


**Figure 4. Single-Cell RNA-Seq Uncovers a Broad NK Cell-Driven Myeloid Cell Reprogramming from Pro-tumorigenic to Anti-tumorigenic Pathways**

Single-cell RNA-seq of 11,651 CD45+ cells isolated from pooled tumors from wild-type or NK cell-depleted *Ptgs*$^{-/-}$ tumor-bearing mice.

(legend continued on next page)

IFN-γ-deficient NK cells (Figure S4F). Finally, the molecular intratumoral landscape of *Ptgs*$^{+/+}$ tumors in GPP mice emulated that of COX-deficient tumors in wild-type mice (Figure S4G), in keeping with their regressive growth profile (Figure 2E). Collectively, these data demonstrated a key role for PGE2 acting on EP2 and EP4 in modulating the intratumoral inflammatory response and identified IFN-γ-secreting NK cells as drivers of a profound TME switch conducive to adaptive immunity.

Next, to comprehensively examine the impact of NK cells on the transcriptional profile of multiple tumor-infiltrating immune cells, we resorted to single-cell RNA sequencing. Analysis of CD45$^{+}$ cells isolated from early COX-deficient tumors implanted in control or NK cell-depleted hosts revealed the presence of 18 different immune cell clusters (Figure 4A; Table S1). Apart from NK cells themselves, the abundance of other immune cell populations remained mostly unaltered by NK cell depletion, which is in agreement with our flow cytometry analysis at this same time point. From the CI inflammatory mediators found to be upregulated in *Ptgs*$^{-/-}$ tumors (Figures 3D and 3E), transcripts encoding for Eomesodermin, Perforin-1, and CCL5 were largely restricted to the NK cell cluster (Figure S5D). This was also the case for granzyme B and IFN-γ, identifying NK cells as the major early source of these effector molecules and as the most likely cellular target responsible for the early phenotype in GPP mice (Figures S5D and S5E). Transcripts for other CI factors critical for CTL recruitment, such as CXCL9 or CXCL10 (Chow et al., 2019; Dangaj et al., 2019; Spranger et al., 2017), were conversely largely confined to myeloid cell populations (Figures 4B, S5E, and S5F). Yet, in agreement with our bulk tumor analysis, their levels were significantly reduced following depletion of NK cells.

Differential gene expression analysis and GSEA of monocytes and TAMs further demonstrated the orchestrating function of NK cells in TME polarization toward an inflammatory profile conducive to effector T cell infiltration. A paired comparison of multiple monocyte and TAM clusters showed pronounced and consistent enrichment in IFN-γ, allograft rejection and oxidative phosphorylation signaling in NK cell-proficient mice (Figures 4C and 4D; Table S2). Conversely, upregulation of "Hypoxia," "TNF-α-signaling via NF-κB" (nuclear factor κB), "Glycolysis," "PI3K signaling" (phosphatidylinositol 3-kinase signaling), "TGF-β signaling," gene sets, and other pro-tumorigenic inflammatory pathways (DeNardo and Ruffell, 2019; Mantovani et al., 2017) was common in NK cell-depleted tumors (Figure 4D; Table S3). Together, these data support a model whereby early NK cell IFN-γ production drives extensive myeloid cell polarization and a wide-ranging TME makeover characteristic of T cell-inflamed tumors.

## Opposing Tumor Inflammatory Profiles Associated with COX-2 Expression and NK Cell Prevalence in Human Cancer

To investigate whether molecular characteristics of NK cell-driven inflammatory TMEs were conserved across human cancers and associated with the COX-2/PGE2 pathway, we interrogated transcriptomic datasets of multiple patient-derived tumor types by using data from The Cancer Genome Atlas (TCGA; https://cancergenome.nih.gov). We first examined the association of COX-2 itself with the inflammatory factors whose expression was regulated by the COX-2/PGE2/EP2-4 axis and NK cell activity in the mouse models and represent canonical mediators often linked with CP and CI inflammation in human cancer (Figures 3D and S4H). This analysis showed unambiguous positive correlations between transcript levels of *PTGS2*, encoding for COX-2, and the levels of CP factors induced by cancer cell-intrinsic COX-2 in murine tumors pan-cancer (Figure 5A). In contrast, CI mediators whose intratumoral expression was elevated by NK cell activity in COX-deficient tumors showed an inverse correlation with *PTGS2* in many malignancies (Figure 5A). Further stratification of cancer types revealed pronounced subtype-specific anti-correlations of *PTGS2* with CI genes, as shown for triple-negative breast cancer (TNBC) in both TCGA and Molecular Taxonomy of Breast Cancer International Consortium (METABRIC) (Curtis et al., 2012) datasets (Figure S6D).

The positive and negative association of COX-2 with CP and CI factors, respectively, were highly statistically significant in tumors, such as lung adenocarcinoma (LUAD) and head and neck squamous cell carcinoma (HNSC) (Figure 5B). In these tumor types, uniform and contrasting expression patterns of CI genes were found by comparing tumors with high or low NK cell infiltration that were inferred using a signature of three selective and defining NK cell markers (Danaher et al., 2018; Figure 5C; Table S5).

Additional bioinformatics analysis pan-cancer showed that COX-2 expression was positively associated with the intratumoral abundance of fibroblasts, endothelial cells, and neutrophils. In contrast, COX-2 transcripts were inversely correlated with various immune subsets in many malignancies, including NK cells and CTLs (Figures 5D, S6E, and S6F). Collectively, these data are in line with our findings in mice, uncovering antagonistic inflammatory profiles linked to intratumoral NK cell levels and COX-2/PGE2 pathway activity in human cancer.

## A Mouse-Derived Inflammatory Gene Signature Exhibits Independent Prognostic Utility across Multiple Malignancies

To assess the prognostic value of these opposing inflammatory tumor profiles, we performed univariate and multivariate survival analysis by using Cox proportional hazards regression models. We focused on cancer types in which we found evidence of divergent inflammatory profiles associated with COX-2 expression levels, such as LUAD, HNSC, TNBC, metastatic skin cutaneous melanoma (MSKCM), cervical squamous cell carcinoma and endocervical adenocarcinoma (CESC), kidney renal clear cell carcinoma (KIRC), or ovarian cancer (OV). To integrate both CP and CI mediators whose expression was controlled by tumor cell-derived PGE2 in mice, we calculated a ratio between the combined average expression of human homologs of these

(A) t-SNE plots showing the clustering and distribution. Each point represents a single cell colored according to cluster designation.
(B) *Cxcl9* and *Cxcl10* gene expression analysis in all cell clusters shown in (A). Data are expressed as normalized counts-per-million (CPM), unpaired Student's t test.
(C) Enrichment analysis for hallmark IFN-γ response gene set in various monocyte and TAM clusters.
(D) Single-sample GSEA of all hallmark gene sets in the same myeloid populations as in (C).

CellPress
OPEN ACCESS

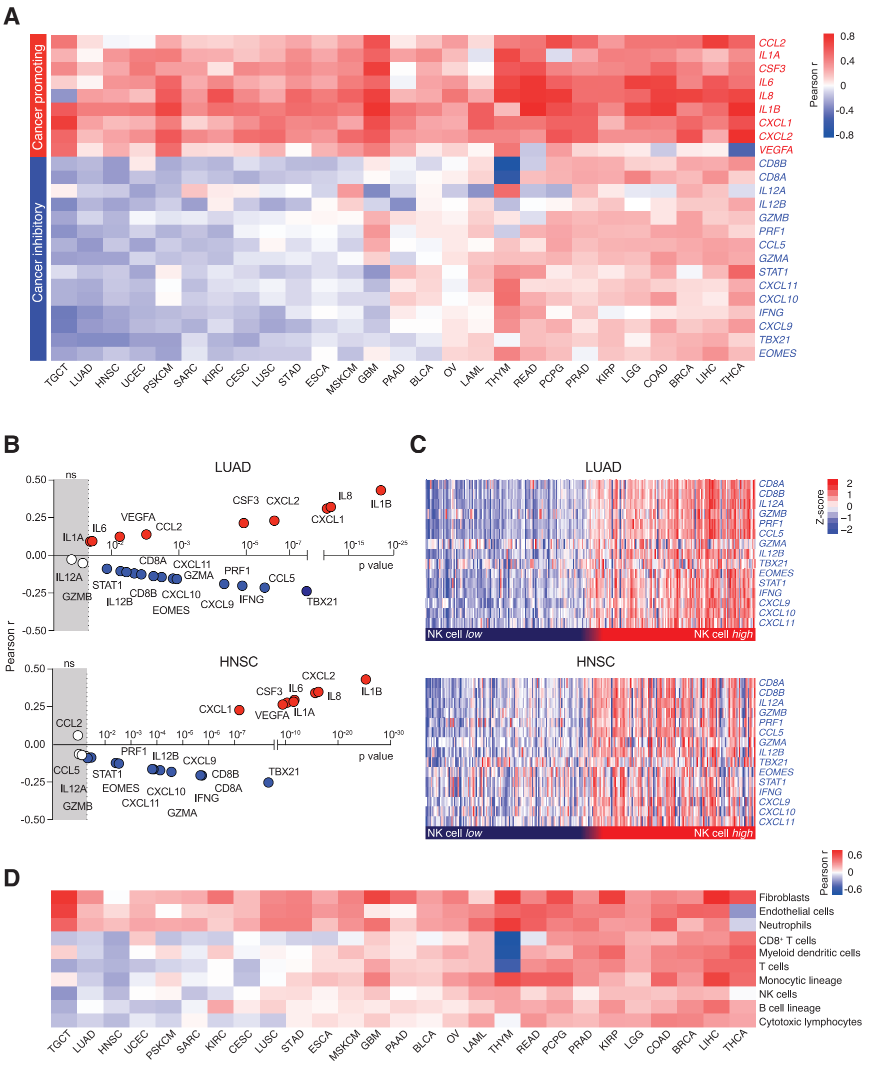


**Figure 5. COX-2 Expression Delineates Cancer-Promoting from Cancer-Inhibitory Inflammation in Human Cancers**

(A) Heatmap showing the Pearson correlation coefficient of *PTGS2* expression with the mouse-derived COX-IS genes across various human datasets from TCGA: testicular germ cell tumors (TGCT; n = 155), lung adenocarcinoma (LUAD; n = 512), head and neck squamous cell carcinoma (HNSC; n = 517), uterine corpus endometrial carcinoma (UCEC; n = 530), primary skin cutaneous melanoma (PSKCM; n = 115), sarcoma (SARC; n = 259), kidney renal clear cell carcinoma (KIRC;, n = 516), cervical and endocervical cancers (CESCs; n = 305), lung squamous cell carcinoma (LUSC; n = 487), stomach adenocarcinoma (STAD; n = 412), esophageal carcinoma (ESCA; n = 183), metastatic skin cutaneous melanoma (MSKCM; n = 357), pancreatic adenocarcinoma (PAAD; n = 178), glioblastoma multiforme (GBM; n = 166), bladder urothelial carcinoma (BLCA; n = 408), ovarian cancer (OV; n = 305), acute myeloid leukemia (LAML; n = 173), thymoma (THYM; n = 120), rectum adenocarcinoma (READ; n = 156), prostate adenocarcinoma (PRAD; n = 495), pheochromocytoma and paraganglioma (PCPG; n = 183), kidney renal papillary cell carcinoma (KIRP; n = 286), brain lower grade glioma (LGG; n = 528), colon adenocarcinoma (COAD; n = 445), breast invasive carcinoma (BRCA; n = 976), liver hepatocellular carcinoma (LIHC; n = 370), and thyroid carcinoma (THCA; n = 507).

(B) Correlation analysis of *PTGS2* against COX-IS genes in LUAD and HNSC datasets. The Pearson coefficient and the corresponding p value are shown.

(C) CI gene expression in NK cell high (top 25%) and NK cell low (bottom 25%) tumors stratified based on an NK cell-specific gene signature (see also Table S4) in LUAD and HNSC datasets.

(D) Pearson correlation coefficient of *PTGS2* expression with the indicated cell populations defined using the Microenvironment cell population (MCP) counter algorithm in all datasets shown in (A).

genes per patient. The stratification of patients according to this COX-2-associated inflammatory signature (referred to as COX-IS) showed that patients with higher COX-IS had worse outcomes in all malignancies tested (Figures 6A and S5A). Notably, COX-IS-based patient stratification was independently prognostic across all seven tumor types when adjusted for age, gender, tumor stage, and other disease-specific features (Figures 6B and S5B). Individual COX-IS elements or combined CP genes showed poor or inconsistent association with survival (Figures 6C and S5C). Signatures of NK cell, CD8$^+$ T cell, IFN-$\gamma$ signaling, or CI genes showed, as expected, comparable prognostic utility. However, none was as robust as the COX-IS, demonstrating the power of integrating pro-tumorigenic inflammatory mediators with known measures of T cell-inflamed tumors to predict overall patient survival.

## The COX-IS Predicts Responses from Immune Checkpoint Therapy in Multiple Cancers

Finally, to evaluate the value of the COX-IS in predicting the outcome from ICB, we examined several datasets of cancer patients treated with anti-CTLA-4, anti-PD-(L)1 or combinations of these therapies. We analyzed nine independent cohorts of patients with melanoma, bladder, gastric, and clear cell renal cancer for which we had access to both the transcriptional profile before ICB treatment and matched response data. For each cohort, we calculated the COX-IS in baseline samples from either responder or non-responder patients, as defined in the original studies. The COX-IS was in every case lower in patients that benefited from ICB than in those that did not, regardless of the cancer type or the immune checkpoint drug used (Figures 7A and S7A). Moreover, the association of the COX-IS with outcome was found independently of the platform used for molecular profiling (RNA sequencing or Nanostring counting) and when analyzing heavily pre-treated or treatment-naive patient cohorts.

To benchmark the COX-IS, we first compared its performance against immune gene signatures related to T cell-inflamed tumors. The COX-IS was more powerful and reliable at distinguishing responders from non-responders than CD8$^+$ T cell, NK cell, or IFN-$\gamma$ signatures or the T cell-inflamed signature (TIS; Ayers

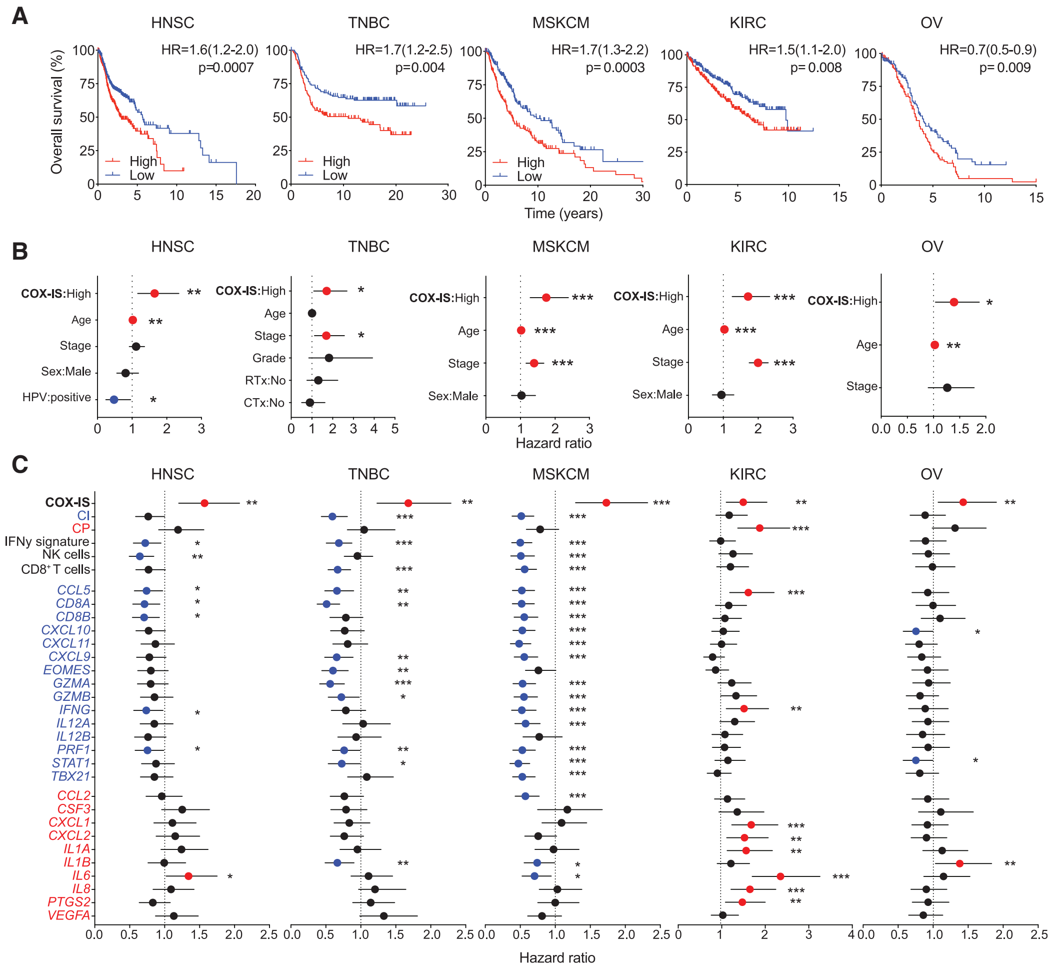


**Figure 6. The COX-IS Is an Independent Prognostic Factor across Various Cancer Types**

(A–C) Survival analysis of HNSC (TCGA, n = 517), TNBC (METABRIC, n = 251), MSKCM (TCGA, n = 357), KIRC (TCGA, n = 516), and OV (TCGA, n = 305) patients stratified according to the COX-IS. (A) Kaplan-Meier survival plots parsed as high versus low on a median cutoff for COX-IS. (B) Forest plots showing a multivariate Cox regression analysis for the indicated risk factors in HNSC, MSKCM, TNBC, KIRC, and OV. (C) Hazard ratio associated with the indicated gene signatures or the individual gene elements of the COX-IS. Hazard ratio (95% confidence interval [CI]), log-rank (Mantel-Cox) test (A–C).

et al., 2017) across the different patient cohorts (Figure 7B). The selective NK cell signature was slightly superior to other CI related signatures, consistent with this innate cell subset playing a key role in dictating the inflammatory profile at the tumor site.

We next performed a deeper bioinformatics analysis on the two largest cohorts available that had a significant number of patients with unambiguous objective responses post-ICB, namely progressive disease (PD) and complete response (CR). In the bladder cancer dataset, 167 and 25 patients were classified as PD or CR, respectively (Mariathasan et al., 2018), and 26 and 9 in the renal cell carcinoma (RCC) cohort (McDermott et al., 2018) following monotherapy with anti-PD-L1. We compared the power of the COX-IS with T cell-inflamed tumor-related gene signatures in discriminating PD versus CR patients as single variables by computing the area-under-the-curve from receiver operating characteristic (ROC) plots. In both datasets, the COX-IS outperformed the other gene signatures (Figure 7C), highlighting its ability to distinguish patients with distinct responses to ICB. In the RCC cohort, the COX-IS was the only single variable gene signature that reached statistical significance in the anti-PD-L1 monotherapy arm. Moreover, an analysis of this entire cohort from the IMmotion150 trial (McDermott et al., 2018) showed that the COX-IS associated with response in the trial arms that received anti-PD-L1 either alone or in combination with anti-VEGF (Figure S7B). However, no association with outcome was found in the arm in which patients were treated with the tyrosine kinase inhibitor sunitinib, showing that the COX-IS predictive value does not merely derive from its overall prognostic utility.

The latter datasets also allowed further benchmarking and testing of the value of combining the COX-IS approach with established biomarkers of ICB response, such as TMB or PD-L1 protein expression. Using the entire datasets, we performed multivariate binomial logistic regression to assess the predictive power of different models. Again, as a single variable, the COX-IS was superior to TIS, IFN-$\gamma$, or CD8$^{+}$ T cell signatures in both bladder cancer and RCC datasets (Figure 7D). In the bladder cancer cohort, both PD-L1 expression and especially TMB were associated with therapeutic response (Figure 7E). Nonetheless,

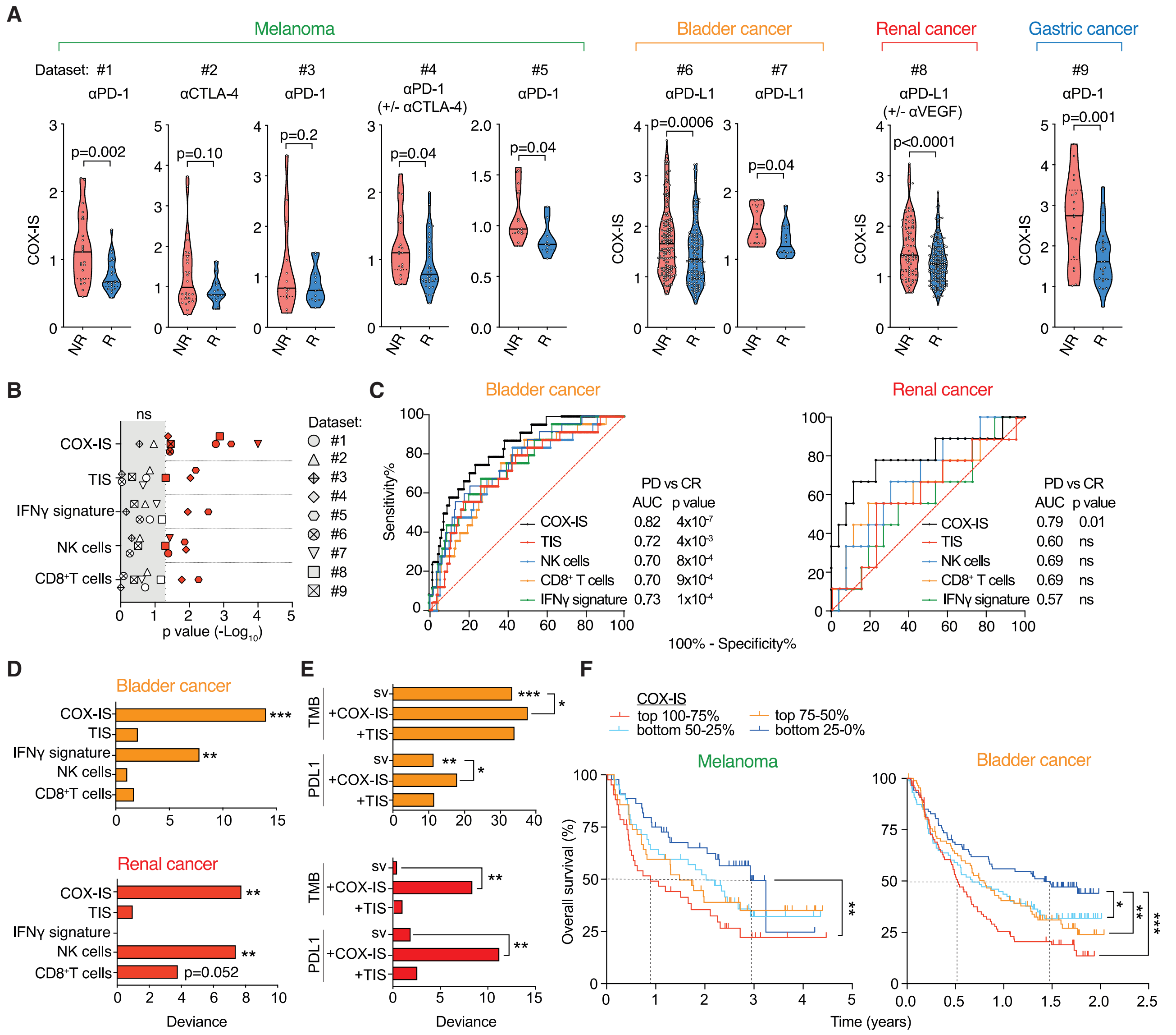


**Figure 7. The COX-IS Predicts Response to ICB in Different Tumor Types**

(A) Analysis of COX-IS at baseline in responder (R) and non-responder (NR) groups in melanoma (dataset 1: Riaz et al., 2017; 2: Van Allen et al., 2015; 3: Hugo et al., 2016; 4: Gide et al., 2019; 5: Chen et al., 2016), bladder (dataset 6: Mariathasan et al., 2018; 7: Snyder et al., 2017), renal (dataset 8: McDermott et al., 2018), and gastric (dataset 9: Kim et al., 2018) cancer patients as defined in the original studies (see STAR Methods).

(B) Analysis of COX-IS; TIS; and IFN-γ, NK cell, and CD8+ T cell signatures (see Table S4) at baseline in R and NR patients shown in (A). The p value ($-\log_{10}$) for each comparison is plotted.

(C) ROC analysis for COX-IS; TIS; and IFN-γ, NK cell, and CD8+ T cell signatures in PD versus CR patient from datasets 6 and 8. The area under the ROC curve was used to quantify response prediction.

(D and E) Explained variance (deviance) in patient response for generalized linear models fit using single variables (sv) (D) or their combinations with TMB or PD-L1 expression (E) in dataset 6 and 8. Chi-square test was used to compare nested models.

(F) Survival of melanoma (pooled datasets 1, 2, 3, and 4) and bladder cancer (dataset 6) patients stratified in quantiles according to their COX-IS. Log-rank (Mantel-Cox) test.

combining either TMB or PD-L1 with the COX-IS, but not with the TIS, achieved a statistically significant improvement in predictive power compared to either model alone. In the RCC cohort, in contrast, neither TMB nor PD-L1 showed a predictive value as a single variable, whereas the COX-IS did (Figure 7E).

Last, we investigated the value of the COX-IS in predicting survival from ICB stratifying patients by using a median cutoff or four quantiles according to their COX-IS score at baseline. Patients with a lower COX-IS benefited significantly more in a combined analysis of four independent melanoma cohorts, with a 2-year difference in the median survival between the lowest and top quantiles (Figures 7F and S7C). Importantly, this association with survival remained significant when adjusting for prior ICB treatment or for the dataset analyzed (Figure S7D). In the

melanoma cohort 2 (Van Allen et al., 2015), which is well known for the association of TMB with response, no trend was observed when patients were stratified based on CP or CI signatures separately, whereas the COX-IS strongly predicted patient survival (Figure S7E). In the bladder cancer cohort subjected to PD-L1 blockade, patients with a higher baseline COX-IS similarly had a worse outcome (Figures 7F and S7C), with the top group showing a median survival of only 6 months compared to more than 1.5 years in the bottom quarter. Once more, the predictive power of the COX-IS was independent of TMB or of the occurrence of visceral or liver metastasis, with both features being particularly informative in this cohort (Figure S7D). These results extended the predictive value of the COX-IS to survival post-ICB therapy and established the approach of combining CP and CI mediators as a potent indicator of outcome from both anti-CTLA-4 and/or PD-(L)1 therapy.

## DISCUSSION

Tumor-associated inflammation is an established cancer hallmark linked to several features of malignant tumors (Hanahan and Weinberg, 2011). Accordingly, inflammatory mediators and signaling pathways commonly found in clinically apparent tumors constitute adverse prognostic factors and promote therapy resistance (Mantovani et al., 2008; Shalapour and Karin, 2019). The recent success of ICB therapy has greatly reinvigorated the study of tumor immunology and inflammation and, in particular, the identification of immune correlates of outcome to ICB, such as tumor infiltration by T cells (Blank et al., 2016; Fridman et al., 2017; Salmon et al., 2019; Topalian et al., 2016). The principles and rules that control the establishment of these so-called hot T cell-inflamed tumors remain poorly understood (Havel et al., 2019; Topalian et al., 2016). Here, we set out to delineate leading orchestrators of hot TMEs by using mouse models with equal TMB but dichotomous immune-dependent progressive or regressive tumor fates coupled to distinctive antagonistic inflammatory profiles. These model systems allowed us to tease apart the instructive pathways of T cell-mediated tumor control independently of the antigenic determinants of the cancer cells themselves. Unexpectedly, the eradication of COX-deficient tumors still occurred in mice lacking the major signal transduction pathways that normally underlie and bridge innate and adaptive immune responses. Instead, our analysis singled out NK cells as primary instructors of a TME characterized by cancer-restraining inflammation and required for CTL-mediated tumor eradication.

NK cells have been frequently implicated in the control of hematological malignancies (Guillerey et al., 2016), and our findings add to a growing list of recent studies highlighting a role for this innate lymphocyte subset in immune surveillance of solid neoplasms (André et al., 2018; Barrow et al., 2018; Barry et al., 2018; Böttcher et al., 2018; Lavin et al., 2017; Molgora et al., 2017; Nicolai et al., 2020). The functions of NK cells in cancer are pleiotropic, ranging from direct sensing and killing of transformed cells to stimulating T cell-mediated immunity (Morvan and Lanier, 2016; Vesely et al., 2011). Our mouse models uncovered both types of cancer suppressive activities; however, complete tumor regression fully relied on NK cells linking innate to adaptive tumor immunity. Thus, our findings are consistent with a temporal sequence of events whereby NK cells directly contribute to cancer cell killing while simultaneously eliciting a broad inflammatory TME switch that precedes and stimulates T cell-mediated cancer immunity.

Deep transcriptional profiling exposed a stark upregulation of classic mediators and pathways associated with hot tumors that coincided with NK cell accumulation. Single-cell RNA sequencing identified NK cells as the direct and unique source of key CI factors and as the instigators of CI pathways expressed more widely by DCs, monocytes, or TAMs (Chow et al., 2019; Dangaj et al., 2019; DeNardo and Ruffell, 2019; Mantovani et al., 2017; Spranger et al., 2017; Wculek et al., 2020). Accordingly, pronounced polarization toward IFN-$\gamma$ signaling and other CI pathways by various prevalent myeloid cell populations relied on NK cells. In their absence, those same populations showed evidence of pro-tumorigenic molecular programs. Of note, recent studies established how IFN-$\gamma$ produced locally by tumor-reactive T cells can act on bystander distant tumor cells and promote broad remodeling of the TME (Hoekstra et al., 2020; Thibaut et al., 2020). In our experimental settings, we found that IFN-$\gamma$ production from NK cells, but not from NK T, $\gamma\delta$, or $\alpha\beta$ T cells, was necessary and sufficient for sculpting the early inflammatory TME toward CI inflammation.

The profound NK and T cell-dependent suppression of tumor growth was impaired by natural or restored expression of COX-2 in cancer cells. The COX-2/PGE2 pathway is associated with malignant cancer growth (Wang and Dubois, 2010) and promotes immune evasion (Böttcher et al., 2018; Zelenay and Reis e Sousa, 2016; Zelenay et al., 2015). Given the expression of the PGE2 receptors EP2 and EP4 in multiple cell types (Furuyashiki and Narumiya, 2011; Kalinski, 2012), the cellular targets of PGE2 in cancer are potentially several. It is indeed likely that the loss of EP4 on CTLs contributed to tumor control in GPP mice, which is similar to what happens during chronic viral infections (Chen et al., 2015). However, our analysis of the early cellular and molecular profile of the TME pinpointed NK cells as the primary target of PGE2/EP2-4 signaling, uncovering a specific cellular and molecular therapeutic target.

Bioinformatics analysis of numerous cancer patient datasets demonstrated the translational relevance of our findings and pointed to the COX-2/PGE2 and NK cell/IFN-$\gamma$ pathways as putative determinants of the TME in many human malignancies. Evidence for antagonistic expression of the very same CP and CI mediators that were controlled by these pathways in the mouse models was found in many malignancies. Furthermore, *PTGS2* transcript levels were significantly associated with higher neutrophil numbers and lower cytotoxic cell abundance, mirroring the findings in the mouse models. This observation, in turn, implied that COX-2 levels do not simply reflect differential overall leukocyte infiltration but rather qualitative changes in tumor immune infiltrate composition.

Our analysis established the independent prognostic relevance of integrating pro- and anti-tumorigenic inflammatory mediators in one single indicator as a means to predict overall patient survival. Crucially, this approach associated with outcome from ICB independently of the immune checkpoint inhibitor drug used or the cancer type analyzed. Patients with low COX-IS were consistently enriched within the responder group and had better outcomes and was the case across several independent patient cohorts (Chen et al., 2016; Gide et al., 2019; Hugo et

al., 2016; Kim et al., 2018; Mariathasan et al., 2018; McDermott et al., 2018; Riaz et al., 2017; Snyder et al., 2017; Van Allen et al., 2015) regardless of the method used to determine gene expression levels. Notably, the COX-IS outperformed previously published gene signatures related to T cell-inflamed tumors in terms of both prognostic and predictive power. Furthermore, it demonstrated independent predictive power even in patient cohorts for which TMB or PD-L1 expression did not associate with outcome.

These findings are even more striking considering the COX-IS was devised from the analysis of a handful of established CP and CI inflammatory mediators in mouse cancer models. As such, the COX-IS has neither been refined nor optimized for human analysis, circumventing the issues associated with overfitting and exposing the notable parallels between the TME in mice and humans. It is likely that its predictive power can be enhanced and broadened by future bioinformatics analysis, and its actual potential as a companion diagnostic will require further testing in a prospective study. We speculate that the superior power of the COX-IS over signatures of CI inflammation originates from combining in one single index, surrogate markers of two intimately linked hallmarks of cancer, tumor-promoting inflammation and evasion of immunity (Hanahan and Weinberg, 2011). The advantage of multigene gene signatures over single markers is well recognized (Ayers et al., 2017; Chen et al., 2016; Cristescu et al., 2018; Rooney et al., 2015) and is of particular value in complex systems, such as the TME for which, arguably, no single inflammatory mediator can be attributed as having exclusive tumor promoting or suppressive properties.

In conclusion, our parallel analysis of mouse cancer models and human patient datasets revealed an instructive function for NK cells in initiating an anti-tumorigenic inflammatory response distinctive of hot T cell-inflamed tumors. Moreover, the COX-2/PGE2/EP2-4 axis emerges as a major conserved determinant by which numerous cancer types modulate their surrounding environment and avoid immune-mediated elimination. These findings have direct clinical implications and propose that monitoring the COX-IS could be a powerful indicator of ICB outcome and possibly other immune therapies.

## Limitations of Study

The signals and pathways that initiate immune responses against malignant cells remain poorly understood. Based on various experimental models and readouts, we concluded that NK cells are necessary and sufficient to trigger CTL-dependent anti-tumor immunity. Notably, the eradication of tumors was not compromised in hosts deficient in the major signaling pathways that drive immunity against microbes. However, our experimental systems could not discriminate whether these pathways were truly dispensable or rather redundant. Dissecting these scenarios would require the use of mice double or triple deficient in these central immune signaling nodes. Similarly, we showed that rendering NK cells insensitive to the effects of PGE2 was sufficient to elicit robust anti-tumor immunity in GPP mice. Yet, this experimental system did not allow us to formally rule out a potential role for another EP4 and granzyme-B-expressing cell population or of an EP2-expressing cell subset, besides NK cells, that might contribute to tumor control in GPP mice. The use of alternative Cre-driver lines more selective to NK cells as well as a *Ptger2* conditional, instead of a germline mutant, allele would be of interest to further explore the cellular targets of PGE2 and their contribution to tumor inflammation and progression. Finally, we tested the human relevance of our murine findings by mining the tumor transcriptional profile of patients with advanced cancer. Future work that encompasses the analysis of early cancer lesions is warranted to further assess the role of tumor-infiltrating NK cells as primary instructors of hot T cell-inflamed tumors in humans.

## STAR★METHODS

Detailed methods are provided in the online version of this paper and include the following:

- KEY RESOURCES TABLE
- RESOURCE AVAILABILITY
  - Lead Contact
  - Materials Availability
  - Data and Code Availability
- EXPERIMENTAL MODEL AND SUBJECT DETAILS
  - Mice
  - Cancer Cell Lines
  - Mouse Procedures
- METHOD DETAILS
  - Quantitative RT-PCR
  - FACS analysis
  - Immunofluorescence Analysis
  - NK cell *in vitro* killing and live tracking
  - Sorting of TAMs
  - Adoptive NK cell transfer
  - 3′-mRNA sequencing and analysis
  - Single cell mRNA sequencing and analysis
  - Bioinformatic Analysis of Patient Datasets
- QUANTIFICATION AND STATISTICAL ANALYSIS

## SUPPLEMENTAL INFORMATION

Supplemental Information can be found online at https://doi.org/10.1016/j.immuni.2020.10.020.

## ACKNOWLEDGMENTS

This work was supported by a Cancer Research UK Institute Award (A19258) to S.Z. We thank colleagues at CRUK Manchester Institute core facilities, in particular, Biological Resource Unit, Transgenic Breeding, Molecular Biology, Histology, and Flow Cytometry. E.B. was supported by an EMBO long-term fellowship (ALTF-69-2016) and an EMBO advanced fellowship (aALTF-638-2018). C.P.B. was funded by the National Institute for Health Research Manchester Biomedical Research Centre. G.J. was supported by a scholarship from The Society of Swedish Engineers in Great Britain. K.W.-B. was supported by a doctoral studentship from the Medical Research Council. C.R.e.S. was supported by The Francis Crick Institute, which receives core funding from Cancer Research UK (FC001136), the UK Medical Research Council (FC001136), and the Wellcome Trust (FC001136), by an ERC Advanced Investigator grant (AdG 268670), by a Wellcome Investigator Award (WT106973MA), and by a prize from the Louis-Jeantet Foundation. N.G. was supported by an Imperial Confidence in Concept Scheme (RSRO_P71752). D.M.D. was supported by a Wellcome Trust Investigator Award (110091/Z/15/Z). We acknowledge Richard Meyer (Vanderbilt University Medical Center), David Baltimore (California Institute of Technology), and Susan M Kaech (Yale University School of Medicine) for generously providing and permitting use of GPP mice. We also thank all investigators and organizations, such as Genentech/gRED for making publicly available or generously sharing patient datasets.

## AUTHOR CONTRIBUTIONS

E.B. designed and conducted experiments, analyzed data, and wrote the manuscript with assistance from G.J., V.S.P., K.W.-B., E.F., C.R.B., S.M., S.-C.C., and C.P.C.-G. C.P.B. performed bioinformatics analysis with help from E.B., S.S., and A. Moeini. N.R., B.S.-S., S.J., A. Mantovani, C.R.e.S., N.G., and D.M.D. provided oversight and reagents. S.Z. conceived and oversaw the study, analyzed data, and wrote the manuscript.

## DECLARATION OF INTERESTS

The COX-IS is the subject of a patent application (WO2019243567A1) in which E.B., C.P.B., and S.Z. are listed as inventors.

Received: October 29, 2019
Revised: August 31, 2020
Accepted: October 23, 2020
Published: November 20, 2020

## REFERENCES

André, P., Denis, C., Soulas, C., Bourbon-Caillet, C., Lopez, J., Arnoux, T., Bléry, M., Bonnafous, C., Gauthier, L., Morel, A., et al. (2018). Anti-NKG2A mAb is a checkpoint inhibitor that promotes anti-tumor immunity by unleashing both T and NK cells. Cell *175*, 1731–1743.e13.

Ayers, M., Lunceford, J., Nebozhyn, M., Murphy, E., Loboda, A., Kaufman, D.R., Albright, A., Cheng, J.D., Kang, S.P., Shankaran, V., et al. (2017). IFN-γ-related mRNA profile predicts clinical response to PD-1 blockade. J. Clin. Invest. *127*, 2930–2940.

Barrow, A.D., Edeling, M.A., Trifonov, V., Luo, J., Goyal, P., Bohl, B., Bando, J.K., Kim, A.H., Walker, J., Andahazy, M., et al. (2018). Natural killer cells control tumor growth by sensing a growth factor. Cell *172*, 534–548.e519.

Barry, K.C., Hsu, J., Broz, M.L., Cueto, F.J., Binnewies, M., Combes, A.J., Nelson, A.E., Loo, K., Kumar, R., Rosenblum, M.D., et al. (2018). A natural killer-dendritic cell axis defines checkpoint therapy-responsive tumor microenvironments. Nat. Med. *24*, 1178–1191.

Becht, E., Giraldo, N.A., Lacroix, L., Buttard, B., Elarouci, N., Petitprez, F., Selves, J., Laurent-Puig, P., Sautès-Fridman, C., Fridman, W.H., and de Reyniès, A. (2016). Estimating the population abundance of tissue-infiltrating immune and stromal cell populations using gene expression. Genome Biol. *17*, 218.

Binnewies, M., Roberts, E.W., Kersten, K., Chan, V., Fearon, D.F., Merad, M., Coussens, L.M., Gabrilovich, D.I., Ostrand-Rosenberg, S., Hedrick, C.C., et al. (2018). Understanding the tumor immune microenvironment (TIME) for effective therapy. Nat. Med. *24*, 541–550.

Blank, C.U., Haanen, J.B., Ribas, A., and Schumacher, T.N. (2016). Cancer Immunology. The ''cancer immunogram''. Science *352*, 658–660.

Böttcher, J.P., Bonavita, E., Chakravarty, P., Blees, H., Cabeza-Cabrerizo, M., Sammicheli, S., Rogers, N.C., Sahai, E., Zelenay, S., and Reis E Sousa, C. (2018). NK cells stimulate recruitment of cDC1 into the tumor microenvironment promoting cancer immune control. Cell *172*, 1022–1037.e14.

Chen, J.H., Perry, C.J., Tsui, Y.-C., Staron, M.M., Parish, I.A., Dominguez, C.X., Rosenberg, D.W., and Kaech, S.M. (2015). Prostaglandin E2 and programmed cell death 1 signaling coordinately impair CTL function and survival during chronic viral infection. Nat. Med. *21*, 327–334.

Chen, P.-L., Roh, W., Reuben, A., Cooper, Z.A., Spencer, C.N., Prieto, P.A., Miller, J.P., Bassett, R.L., Gopalakrishnan, V., Wani, K., et al. (2016). Analysis of immune signatures in longitudinal tumor samples yields insight into biomarkers of response and mechanisms of resistance to immune checkpoint blockade. Cancer Discov. *6*, 827–837.

Chow, M.T., Ozga, A.J., Servis, R.L., Frederick, D.T., Lo, J.A., Fisher, D.E., Freeman, G.J., Boland, G.M., and Luster, A.D. (2019). Intratumoral activity of the CXCR3 chemokine system is required for the efficacy of anti-PD-1 therapy. Immunity *50*, 1498–1512.e5.

Coussens, L.M., Zitvogel, L., and Palucka, A.K. (2013). Neutralizing tumor-promoting chronic inflammation: a magic bullet? Science *339*, 286–291.

Cristescu, R., Mogg, R., Ayers, M., Albright, A., Murphy, E., Yearley, J., Sher, X., Liu, X.Q., Lu, H., Nebozhyn, M., et al. (2018). Pan-tumor genomic biomarkers for PD-1 checkpoint blockade-based immunotherapy. Science *362*, eaar3593.

Curtis, C., Shah, S.P., Chin, S.-F., Turashvili, G., Rueda, O.M., Dunning, M.J., Speed, D., Lynch, A.G., Samarajiwa, S., Yuan, Y., et al. (2012). The genomic and transcriptomic architecture of 2,000 breast tumours reveals novel subgroups. Nature *486*, 346–352.

Danaher, P., Warren, S., Dennis, L., D'Amico, L., White, A., Disis, M.L., Geller, M.A., Odunsi, K., Beechem, J., and Fling, S.P. (2017). Gene expression markers of tumor infiltrating leukocytes. J. Immunother. Cancer *5*, 18.

Danaher, P., Warren, S., Lu, R., Samayoa, J., Sullivan, A., Pekker, I., Wallden, B., Marincola, F.M., and Cesano, A. (2018). Pan-cancer adaptive immune resistance as defined by the tumor inflammation signature (TIS): results from The Cancer Genome Atlas (TCGA). J. Immunother. Cancer *6*, 63.

Dangaj, D., Bruand, M., Grimm, A.J., Ronet, C., Barras, D., Duttagupta, P.A., Lanitis, E., Duraiswamy, J., Tanyi, J.L., Benencia, F., et al. (2019). Cooperation between constitutive and inducible chemokines enables T cell engraftment and immune attack in solid tumors. Cancer Cell *35*, 885–900.e10.

DeNardo, D.G., and Ruffell, B. (2019). Macrophages as regulators of tumour immunity and immunotherapy. Nat. Rev. Immunol. *19*, 369–382.

Dhomen, N., Reis-Filho, J.S., da Rocha Dias, S., Hayward, R., Savage, K., Delmas, V., Larue, L., Pritchard, C., and Marais, R. (2009). Oncogenic Braf induces melanocyte senescence and melanoma in mice. Cancer Cell *15*, 294–303.

Dobin, A., Davis, C.A., Schlesinger, F., Drenkow, J., Zaleski, C., Jha, S., Batut, P., Chaisson, M., and Gingeras, T.R. (2013). STAR: ultrafast universal RNA-seq aligner. Bioinformatics *29*, 15–21.

Fridman, W.H., Zitvogel, L., Sautès-Fridman, C., and Kroemer, G. (2017). The immune contexture in cancer prognosis and treatment. Nat. Rev. Clin. Oncol. *14*, 717–734.

Fridman, W.-H., Pagès, F., Sautès-Fridman, C., and Galon, J. (2012). The immune contexture in human tumours: impact on clinical outcome. Nat. Rev. Cancer *12*, 298–306.

Furuyashiki, T., and Narumiya, S. (2011). Stress responses: the contribution of prostaglandin E(2) and its receptors. Nat. Rev. Endocrinol. *7*, 163–175.

Gentles, A.J., Newman, A.M., Liu, C.L., Bratman, S.V., Feng, W., Kim, D., Nair, V.S., Xu, Y., Khuong, A., Hoang, C.D., et al. (2015). The prognostic landscape of genes and infiltrating immune cells across human cancers. Nat. Med. *21*, 938–945.

Gide, T.N., Quek, C., Menzies, A.M., Tasker, A.T., Shang, P., Holst, J., Madore, J., Lim, S.Y., Velickovic, R., Wongchenko, M., et al. (2019). Distinct immune cell populations define response to anti-PD-1 monotherapy and anti-PD-1/anti-CTLA-4 combined therapy. Cancer Cell *35*, 238–255.e6.

Guillerey, C., Huntington, N.D., and Smyth, M.J. (2016). Targeting natural killer cells in cancer immunotherapy. Nat. Immunol. *17*, 1025–1036.

Hanahan, D., and Weinberg, R.A. (2011). Hallmarks of cancer: the next generation. Cell *144*, 646–674.

Havel, J.J., Chowell, D., and Chan, T.A. (2019). The evolving landscape of biomarkers for checkpoint inhibitor immunotherapy. Nat. Rev. Cancer *19*, 133–150.

Herbst, R.S., Soria, J.-C., Kowanetz, M., Fine, G.D., Hamid, O., Gordon, M.S., Sosman, J.A., McDermott, D.F., Powderly, J.D., Gettinger, S.N., et al. (2014). Predictive correlates of response to the anti-PD-L1 antibody MPDL3280A in cancer patients. Nature *515*, 563–567.

Hoekstra, M.E., Bornes, L., Dijkgraaf, F.E., Philips, D., Pardieck, I.N., Toebes, M., Thommen, D.S., van Rheenen, J., and Schumacher, T.N.M. (2020). Long-distance modulation of bystander tumor cells by $CD8^{+}$ T cell-secreted IFNγ. Nat. Cancer *1*, 291–301.

Hugo, W., Zaretsky, J.M., Sun, L., Song, C., Moreno, B.H., Hu-Lieskovan, S., Berent-Maoz, B., Pang, J., Chmielowski, B., Cherry, G., et al. (2016). Genomic and transcriptomic features of response to anti-PD-1 therapy in metastatic melanoma. Cell *165*, 35–44.

CellPress
OPEN ACCESS

Kalinski, P. (2012). Regulation of immune responses by prostaglandin E2. J. Immunol. *188*, 21–28.

Kim, S.T., Cristescu, R., Bass, A.J., Kim, K.-M., Odegaard, J.I., Kim, K., Liu, X.Q., Sher, X., Jung, H., Lee, M., et al. (2018). Comprehensive molecular characterization of clinical responses to PD-1 inhibition in metastatic gastric cancer. Nat. Med. *24*, 1449–1458.

Krämer, A., Green, J., Pollard, J., Jr., and Tugendreich, S. (2014). Causal analysis approaches in Ingenuity Pathway Analysis. Bioinformatics *30*, 523–530.

Lavin, Y., Kobayashi, S., Leader, A., Amir, E.-a.D., Elefant, N., Bigenwald, C., Remark, R., Sweeney, R., Becker, C.D., Levine, J.H., et al. (2017). Innate immune landscape in early lung adenocarcinoma by paired single-cell analyses. Cell *169*, 750–765.e17.

Liao, Y., Smyth, G.K., and Shi, W. (2014). featureCounts: an efficient general purpose program for assigning sequence reads to genomic features. Bioinformatics *30*, 923–930.

Liberzon, A., Birger, C., Thorvaldsdóttir, H., Ghandi, M., Mesirov, J.P., and Tamayo, P. (2015). The Molecular Signatures Database (MSigDB) hallmark gene set collection. Cell Syst. *1*, 417–425.

Mantovani, A., Allavena, P., Sica, A., and Balkwill, F. (2008). Cancer-related inflammation. Nature *454*, 436–444.

Mantovani, A., Marchesi, F., Malesci, A., Laghi, L., and Allavena, P. (2017). Tumour-associated macrophages as treatment targets in oncology. Nat. Rev. Clin. Oncol. *14*, 399–416.

Mariathasan, S., Turley, S.J., Nickles, D., Castiglioni, A., Yuen, K., Wang, Y., Kadel, E.E., III, Koeppen, H., Astarita, J.L., Cubas, R., et al. (2018). TGFβ attenuates tumour response to PD-L1 blockade by contributing to exclusion of T cells. Nature *554*, 544–548.

McDermott, D.F., Huseni, M.A., Atkins, M.B., Motzer, R.J., Rini, B.I., Escudier, B., Fong, L., Joseph, R.W., Pal, S.K., Reeves, J.A., et al. (2018). Clinical activity and molecular correlates of response to atezolizumab alone or in combination with bevacizumab versus sunitinib in renal cell carcinoma. Nat. Med. *24*, 749–757.

McGranahan, N., Furness, A.J.S., Rosenthal, R., Ramskov, S., Lyngaa, R., Saini, S.K., Jamal-Hanjani, M., Wilson, G.A., Birkbak, N.J., Hiley, C.T., et al. (2016). Clonal neoantigens elicit T cell immunoreactivity and sensitivity to immune checkpoint blockade. Science *351*, 1463–1469.

Molgora, M., Bonavita, E., Ponzetta, A., Riva, F., Barbagallo, M., Jaillon, S., Popović, B., Bernardini, G., Magrini, E., Gianni, F., et al. (2017). IL-1R8 is a checkpoint in NK cells regulating anti-tumour and anti-viral activity. Nature *551*, 110–114.

Morvan, M.G., and Lanier, L.L. (2016). NK cells and cancer: you can teach innate cells new tricks. Nat. Rev. Cancer *16*, 7–19.

Nicolai, C.J., Wolf, N., Chang, I.-C., Kirn, G., Marcus, A., Ndubaku, C.O., McWhirter, S.M., and Raulet, D.H. (2020). NK cells mediate clearance of CD8+ T cell-resistant tumors in response to STING agonists. Sci. Immunol. *5*, eaaz2738.

Reich, M., Liefeld, T., Gould, J., Lerner, J., Tamayo, P., and Mesirov, J.P. (2006). GenePattern 2.0. Nat. Genet. *38*, 500–501.

Riaz, N., Havel, J.J., Makarov, V., Desrichard, A., Urba, W.J., Sims, J.S., Hodi, F.S., Martín-Algarra, S., Mandal, R., Sharfman, W.H., et al. (2017). Tumor and microenvironment evolution during immunotherapy with Nivolumab. Cell *171*, 934–949.e16.

Rizvi, N.A., Hellmann, M.D., Snyder, A., Kvistborg, P., Makarov, V., Havel, J.J., Lee, W., Yuan, J., Wong, P., Ho, T.S., et al. (2015). Mutational landscape determines sensitivity to PD-1 blockade in non–small cell lung cancer. Science *348*, 124.

Robinson, M.D., McCarthy, D.J., and Smyth, G.K. (2010). edgeR: a Bioconductor package for differential expression analysis of digital gene expression data. Bioinformatics *26*, 139–140.

Roh, W., Chen, P.-L., Reuben, A., Spencer, C.N., Prieto, P.A., Miller, J.P., Gopalakrishnan, V., Wang, F., Cooper, Z.A., Reddy, S.M., et al. (2017). Integrated molecular analysis of tumor biopsies on sequential CTLA-4 and PD-1 blockade reveals markers of response and resistance. Sci. Transl. Med. *9*, eaah3560.

Rooney, M.S., Shukla, S.A., Wu, C.J., Getz, G., and Hacohen, N. (2015). Molecular and genetic properties of tumors associated with local immune cytolytic activity. Cell *160*, 48–61.

Salmon, H., Idoyaga, J., Rahman, A., Leboeuf, M., Remark, R., Jordan, S., Casanova-Acebes, M., Khudoynazarova, M., Agudo, J., Tung, N., et al. (2016). Expansion and activation of CD103(+) dendritic cell progenitors at the tumor site enhances tumor responses to therapeutic PD-L1 and BRAF inhibition. Immunity *44*, 924–938.

Salmon, H., Remark, R., Gnjatic, S., and Merad, M. (2019). Host tissue determinants of tumour immunity. Nat. Rev. Cancer *19*, 215–227.

Shalapour, S., and Karin, M. (2019). Pas de deux: control of anti-tumor immunity by cancer-associated inflammation. Immunity *51*, 15–26.

Snyder, A., Makarov, V., Merghoub, T., Yuan, J., Zaretsky, J.M., Desrichard, A., Walsh, L.A., Postow, M.A., Wong, P., Ho, T.S., et al. (2014). Genetic basis for clinical response to CTLA-4 blockade in melanoma. N. Engl. J. Med. *371*, 2189–2199.

Snyder, A., Nathanson, T., Funt, S.A., Ahuja, A., Buros Novik, J., Hellmann, M.D., Chang, E., Aksoy, B.A., Al-Ahmadie, H., Yusko, E., et al. (2017). Contribution of systemic and somatic factors to clinical response and resistance to PD-L1 blockade in urothelial cancer: an exploratory multi-omic analysis. PLoS Med. *14*, e1002309.

Spranger, S., Dai, D., Horton, B., and Gajewski, T.F. (2017). Tumor-residing Batf3 dendritic cells are required for effector T cell trafficking and adoptive T cell therapy. Cancer Cell *31*, 711–723.e4.

Thibaut, R., Bost, P., Milo, I., Cazaux, M., Lemaître, F., Garcia, Z., Amit, I., Breart, B., Cornuot, C., Schwikowski, B., and Bousso, P. (2020). Bystander IFN-γ activity promotes widespread and sustained cytokine signaling altering the tumor microenvironment. Nat. Cancer *1*, 302–314.

Thorsson, V., Gibbs, D.L., Brown, S.D., Wolf, D., Bortone, D.S., Ou Yang, T.-H., Porta-Pardo, E., Gao, G.F., Plaisier, C.L., Eddy, J.A., et al. (2018). The immune landscape of cancer. Immunity *48*, 812–830.e14.

Topalian, S.L., Taube, J.M., Anders, R.A., and Pardoll, D.M. (2016). Mechanism-driven biomarkers to guide immune checkpoint blockade in cancer therapy. Nat. Rev. Cancer *16*, 275–287.

Tumeh, P.C., Harview, C.L., Yearley, J.H., Shintaku, I.P., Taylor, E.J.M., Robert, L., Chmielowski, B., Spasic, M., Henry, G., Ciobanu, V., et al. (2014). PD-1 blockade induces responses by inhibiting adaptive immune resistance. Nature *515*, 568–571.

Van Allen, E.M., Miao, D., Schilling, B., Shukla, S.A., Blank, C., Zimmer, L., Sucker, A., Hillen, U., Foppen, M.H.G., Goldinger, S.M., et al. (2015). Genomic correlates of response to CTLA-4 blockade in metastatic melanoma. Science *350*, 207–211.

Vesely, M.D., Kershaw, M.H., Schreiber, R.D., and Smyth, M.J. (2011). Natural innate and adaptive immunity to cancer. Annu. Rev. Immunol. *29*, 235–271.

Wang, D., and Dubois, R.N. (2010). Eicosanoids and cancer. Nat. Rev. Cancer *10*, 181–193.

Wculek, S.K., Cueto, F.J., Mujal, A.M., Melero, I., Krummel, M.F., and Sancho, D. (2020). Dendritic cells in cancer immunology and immunotherapy. Nat. Rev. Immunol. *20*, 7–24.

Workman, P., Aboagye, E.O., Balkwill, F., Balmain, A., Bruder, G., Chaplin, D.J., Double, J.A., Everitt, J., Farningham, D.A.H., Glennie, M.J., et al. (2010). Guidelines for the welfare and use of animals in cancer research. Br. J. Cancer *102*, 1555–1577.

Zelenay, S., and Reis e Sousa, C. (2016). Reducing prostaglandin E2 production to raise cancer immunogenicity. OncoImmunology *5*, e1123370.

Zelenay, S., van der Veen, A.G., Böttcher, J.P., Snelgrove, K.J., Rogers, N., Acton, S.E., Chakravarty, P., Girotti, M.R., Marais, R., Quezada, S.A., et al. (2015). Cyclooxygenase-dependent tumor growth through evasion of immunity. Cell *162*, 1257–1270.

## STAR★METHODS

### KEY RESOURCES TABLE

| REAGENT or RESOURCE | SOURCE | IDENTIFIER |
|---|---|---|
| Antibodies | | |
| Rabbit monoclonal anti-Cox1 (clone D2G6) | Cell Signaling Technology | Cat# 9896S; RRID: AB_10860249 |
| Rabbit monoclonal anti- Cox2 (clone D5H5) | Cell Signaling Technology | Cat# 12282S; RRID: AB_2571729 |
| Control rat IgG2a, κ Isotype | Biolegend | Cat# 400501; RRID: AB_326523 |
| Control mouse IgG2b, κ Isotype | Biolegend | Cat# 401201; RRID: AB_2744505) |
| anti-Gr1 (clone RB6-8C5) | Biolegend | Cat# 108414; RRID: AB_313379 |
| anti-NK1.1 (clone PK136) | BioXCell | Cat# BE0036; RRID: AB_1107737 |
| Ultra-LEAF Purified anti-Asialo-GM1 Antibody | Biolegend | Cat# 146002; RRID: AB_2562206 |
| anti-CD4 (clone GK1.5) | BioXCell | Cat# BE0003-1; RRID: AB_1107636 |
| anti-CD8alpha (clone YTS169.4) | BioXCell | Cat# BE0117; RRID: AB_10950145 |
| anti-CD49b-APC (clone DX5) | BioLegend | Cat# 103516; RRID: AB_2566101) |
| anti-Ly6G- PE/Dazzle 594 (clone 1A8) | BioLegend | Cat# 127648; RRID: AB_2566319 |
| anti CD4-Alexa700 (RM4-5) | BioLegend | Cat# 100536; RRID: AB_493701 |
| anti-CD8a-PE (clone 53-6.7) | BioLegend | Cat# 100708; RRID: AB_312747 |
| CD45-BV605 (Clone 30-F11) | eBioscience | Cat# 103155; RRID: AB_2650656 |
| CD11b-BV785 (Clone M1/70) | BioLegend | Cat# 101243; RRID: AB_2561373 |
| Ly6C-FITC (Clone AL- 21) | BD Bioscience | Cat# 553104; RRID: AB_394628 |
| F4/80-PE-Cy7 (Clone CI: A3-1) | BioLegend | Cat# 123114; RRID: AB_893478 |
| anti-MHCII I-A/I-E-Alexa700 (Clone M5/114.15.2) | BioLegend | Cat# 107622; RRID: AB_493727 |
| anti-MHCII I-A/I-E- APC- eFluor780 (Clone M5/ 114.15.2) | Thermo Fisher Scientific | Cat# 47-5321-82; RRID: AB_1548783 |
| anti-CD11c-PerCP/Cy5.5 (Clone N418) | BioLegend | Cat# 117328; RRID: AB_2129641 |
| anti- CD103-APC (Clone 2E7) | BioLegend | Cat# 121414; RRID: AB_1227502 |
| anti- CD103-PE (Clone 2E7) | BioLegend | Cat# 121406; RRID: AB_1133989 |
| NK1.1-APC (Clone PK136) | BioLegend | Cat# 108710; RRID: AB_313397 |
| NK1.1- PE (Clone PK136) | BioLegend | Cat# 108708; RRID: AB_313395 |
| CD49b-APC (Clone DX5) | BioLegend | Cat# 108910; RRID: AB_313417 |
| XCR1- Alexa647 (Clone ZET) | BioLegend | Cat# 148214; RRID: AB_2564369 |
| XCR1-BV421 (Clone ZET) | BioLegend | Cat# 148216; RRID: AB_2565230 |
| Siglec-H-BV711 (Clone E50-2440) | BD Bioscience | Cat# 740764; RRID: AB_2740427 |
| IFNγ-eFluor450 (Clone XMG1.2) | Thermo Fisher Scientific | Cat# 48-7311-82; RRID: AB_1834366 |
| CD44-APC-eFluor780 (Clone IM7) | Thermo Fisher Scientific | Cat# 47-0441-82; RRID: AB_1272244 |
| CD3ε-Percp-Cy5.5 (Clone 145-2C11) | BioLegend | Cat# 100328; RRID: AB_893318 |
| CD3ε-APC (Clone 145-2C11) | BioLegend | Cat# 100312; RRID: AB_312677 |
| CD8a-PE (Clone 53-6.7) | BioLegend | Cat# 100708; RRID: AB_312747 |
| CD8a-PE-Cy7 (Clone 53-6.7) | BioLegend | Cat# 100722; RRID: AB_312761 |
| CD4-FITC (Clone RM4-5) | BioLegend | Cat# 100510; RRID: AB_312713 |
| CD4-APC-eFluor780 (Clone GK1.5) | Thermo Fisher Scientific | Cat# 47-0041-82; RRID: AB_11218896 |
| anti-CD16/32 (clone 2.4G2) | BioLegend | Cat# 101302; RRID: AB_312801 |
| affinity purified anti-Ly6G (Clone 1A8) | BD Bioscience | Cat# 551459; RRID: AB_394206 |
| affinity purified anti-NK1.1- biotynilated (Clone PK136) | BioLegend | Cat# 108704; RRID: AB_313391 |
| CD19-eFluor450 (Clone eBio1D3) | Thermo Fisher Scientific | Cat# 48-0193-82; RRID: AB_2734905 |

(*Continued on next page*)

CellPress
OPEN ACCESS

*Continued*

| REAGENT or RESOURCE | SOURCE | IDENTIFIER |
|---|---|---|
| Chemicals, Peptides, and Recombinant Proteins | | |
| Collagenase IV | Worthington Biochemical | Cat# LS004188 |
| DNase I | Roche | Cat#11284932001 |
| Aqua LIVE/Dead-405 nm staining | Thermo Fisher Scientific | Cat# L34957 |
| Brefeldin A Solution (1,000X) | Biolegend | Cat# 420601 |
| Monensin Solution (1,000X) | Biolegend | Cat# 420701 |
| 10 $\mu$m latex beads | Beckman Coulter | Cat# 6602796 |
| OCT embedding medium | Thermo Fisher Scientific | Cat# 12678646 |
| Recombinant FC IL-2 | Thermo Fisher Scientific | Cat# 34-8021-85 |
| CellTrace Calcein Green | Thermo Fisher Scientific | Cat# C34852 |
| Calcein Red- Orange | Thermo Fisher Scientific | Cat# C34851 |
| RLT lysis buffer | QIAGEN | Cat# 79216 |
| PureZOL RNA isolation Reagent | BioRad | Cat# 7326890 |
| Critical Commercial Assays | | |
| RNeasy RNA isolation Kit | QIAGEN | Cat# 74106 |
| Direct-zol RNA MiniPrep Kit | Zymo Research | Cat# R2052 |
| High Capacity cDNA archive Kit | Applied Biosystems | Cat# 4368813 |
| MojoSort Mouse NK Cell Isolation Kit | Biolegned | Cat# 480050 |
| Lexogen QuantSeq 3′-mRNaseq Library Prep Kit FWD | Lexogen | Cat# 015 |
| Chromium Single Cell 3′ Reagent Kit v3 | 10X Genomics | PN- 1000075, PN-120262 |
| Chromium Chip B | 10X Genomics | PN-1000073 |
| Kapa Library Quantification Kit for Illumina sequencing platforms | Kapa Biosystems | Cat# KK4873 |
| Prostaglandin $E_2$ Express ELISA Kit | Cyman Chemical | Cat# 500141 |
| Deposited Data | | |
| Raw and processed data (bulk tumor RNaseq) | This paper | GEO: GSE139044 |
| Raw and processed data (sorted TAM RNaseq) | This paper | GEO: GSE139045 |
| Raw and processed data (single cell RNaseq) | This paper | GEO: GSE139046 |
| Table of cluster biomarkers (scRNaseq) | This paper | Table S1 |
| DEG analysis in Myeloid population (scRNaseq) | This paper | Table S2 |
| Single sample GSEA (scRNaseq) | This paper | Table S3 |
| Ingenuity Pathway Analysis (sorted TAM RNaseq) | This paper | Table S4 |
| Signature gene list (human dataset analysis) | This paper | Table S5 |
| Immune infiltrate in neutrophil-depleted tumor bearing mice (FACS analysis) | This paper | https://doi.org/10.17632/gngxk9mjkm.1 |
| Experimental Models: Cell Lines | | |
| Melanoma C57BL/6 Braf +/LSL-V600E;Tyr::CreERT2 +/o;p16 INK4a−/− cell line | Dhomen et al., 2009 | *Ptgs*$^{+/+}$ |
| Melanoma C57BL/6 Braf +/LSL-V600E;Tyr::CreERT2 +/o;p16 INK4a−/− cell line COX1/2-deficient | Zelenay et al., 2015 | *Ptgs*$^{-/-}$ |
| CT26 | Cancer Research UK Manchester Institute | N/A |
| MC38 | Cancer Research UK Manchester Institute | N/A |
| 4T1 | Cancer Research UK Manchester Institute | N/A |
| CT26 COX2-deficient | Zelenay et al., 2015 | N/A |
| MC38 COX2-deficient | This paper | N/A |
| 4T1 COX1/2-deficient | Zelenay et al., 2015 | N/A |

(*Continued on next page*)

*Continued*

| REAGENT or RESOURCE | SOURCE | IDENTIFIER |
|---|---|---|
| Experimental Models: Organisms/Strains | | |
| Mouse: C57BL/6JOlaHsd | ENVIGO | Stock No: 057 |
| Mouse: BALB/cOlaHsd | ENVIGO | Stock No: 162 |
| Mouse: *Rag1*$^{-/-}$ | Cancer Research UK Manchester Institute | N/A |
| Mouse: *Ifng*$^{-/-}$ | Cancer Research UK Manchester Institute | N/A |
| Mouse: *Tmem173*$^{-/-}$ | Cancer Research UK Manchester Institute | N/A |
| Mouse: *Cgas*$^{-/-}$ | Cancer Research UK Manchester Institute | N/A |
| Mouse: *Trif*$^{-/-}$ | Cancer Research UK Manchester Institute | N/A |
| Mouse: *Mavs*$^{-/-}$ | Cancer Research UK Manchester Institute | N/A |
| Mouse: *Myd88*$^{-/-}$ | Cancer Research UK Manchester Institute | N/A |
| Mouse: Gzmb-Cre *Ptger4*$^{floxed/floxed}$ /*Ptger2*$^{-/-}$ | Cancer Research UK Manchester Institute | N/A |
| Mouse: Gzmb-Cre *Ptger4*$^{floxed/floxed}$ | Cancer Research UK Manchester Institute | N/A |
| Mouse: *Ptger2*$^{-/-}$ | Cancer Research UK Manchester Institute | N/A |
| Mouse: *Tcrd*$^{-/-}$ | Instituto de Medicina Molecular, Lisbon | N/A |
| Oligonucleotides | | |
| *Ccl2*-Taqman probe | Thermo Fisher Scientific | Mm00441242_m1 |
| *Ccl5*-Taqman probe | Thermo Fisher Scientific | Mm01302427_m1 |
| *Cd8a*-Taqman probe | Thermo Fisher Scientific | Mm01182107_g1 |
| *Ptgs2*-Taqman probe | Thermo Fisher Scientific | Mm00478374_m1 |
| *Cxcl1* -Taqman probe | Thermo Fisher Scientific | Mm04207460_m1 |
| *Cxcl10*-Taqman probe | Thermo Fisher Scientific | Mm00445235_m1 |
| *Cxcl2*-Taqman probe | Thermo Fisher Scientific | Mm00436450_m1 |
| *Cxcl9*-Taqman probe | Thermo Fisher Scientific | Mm00434946_m1 |
| *Eomes*-Taqman probe | Thermo Fisher Scientific | Mm01351984_m1 |
| *Csf3*-Taqman probe | Thermo Fisher Scientific | Mm00438334_m1 |
| *Gapdh*-Taqman probe | Thermo Fisher Scientific | Mm99999915_g1 |
| *Gzmb* -Taqman probe | Thermo Fisher Scientific | Mm00442837_m1 |
| *Hprt*-Taqman probe | Thermo Fisher Scientific | Mm03024075_m1 |
| *Ifng*-Taqman probe | Thermo Fisher Scientific | Mm01168134_m1 |
| *Il12a*-Taqman probe | Thermo Fisher Scientific | Mm00434169_m1 |
| *Il12b*-Taqman probe | Thermo Fisher Scientific | Mm01288989_m1 |
| *Il1a*-Taqman probe | Thermo Fisher Scientific | Mm00439620_m1 |
| *Il1b*-Taqman probe | Thermo Fisher Scientific | Mm00434228_m1 |
| *Il6*-Taqman probe | Thermo Fisher Scientific | Mm00446190_m1 |
| *Prf1*-Taqman probe | Thermo Fisher Scientific | Mm00812512_m1 |
| *Stat1*-Taqman probe | Thermo Fisher Scientific | Mm01257286_m1 |
| *Tbx21*-Taqman probe | Thermo Fisher Scientific | Mm00450960_m1 |
| *Vegfa*-Taqman probe | Thermo Fisher Scientific | Mm00437306_m1 |
| Software and Algorithms | | |
| ImageJ/Fiji software | https://imagej.nih.gov/ij/ | version 2.0.0-rc14/1.49 |
| Ingenuity Pathways Analysis software (IPA) | QIAGEN | 2018 update |
| Cell Ranger Pipeline | 10X Genomics | version 3.1 |
| GraphPad Prism | GraphPad Software | version 8.2.0 |
| FlowJo Software | TreeStar FlowJo LLC. | version 10.4.2 |
| Cytobank Software | CytoBank, Inc. | N/A |
| Partek Flow software (Single cell Toolkit) | Partek | build version 8.0.19.0610 |
| R software | R project | N/A |
| Other | | |
| Mouse Diet: RM3 expanded and irradiated | SDS | Code: 801185 |

CellPress
OPEN ACCESS

## RESOURCE AVAILABILITY

### Lead Contact

Information and requests for resources and reagents should be directed to the Lead Contact (santiago.zelenay@cruk.manchester.ac.uk).

### Materials Availability

Requests for cancer cell lines generated in this study can be addressed to the Lead Contact. No new animal strains were generated for this study.

### Data and Code Availability

The RNA sequencing data have been deposited in the NCBI's Gene Expression Omnibus database and can be accessed through the GEO reference Series GSE139047. The accession number for bulk tumor transcriptomes is GSE139044. The accession number for the transcriptome of sorted TAMs is GSE139045. The accession number for the single cell RNA-sequencing data is GSE139046. Immune infiltrate FACS data in neutrophil-depleted tumor-bearing mice have been deposited in Mendeley Data https://doi.org/10.17632/gngxk9mjkm.1.

## EXPERIMENTAL MODEL AND SUBJECT DETAILS

### Mice

Wild-type mice were on a C57BL/6J or Balb/C genetic background (ENVIGO). *Rag1*$^{-/-}$, *Ifng*$^{-/-}$, *Tmem173*$^{-/-}$, *Cgas*$^{-/-}$, *Trif*$^{-/-}$, *Mavs*$^{-/-}$, *Myd88*$^{-/-}$, *Gzmb*-Cre *Ptger4*$^{floxed/floxed}$ *Ptger2*$^{-/-}$, *Gzmb*-Cre *Ptger4*$^{floxed/floxed}$ *and Ptger2*$^{-/-}$ mice in a C57BL/6 background were housed and bred at Cancer Research UK Manchester Institute in specific pathogen-free conditions in individually ventilated cages. *Tcrd*$^{-/-}$ mice were housed and bred at the Instituto de Medicina Molecular, Lisbon. Both male and female mice were used in procedures and they were randomly assigned to experimental groups. All procedures involving animals were performed under the PDCC31AAF license, in accordance with ARRIVE guidelines and National Home Office regulations under the Animals (Scientific Procedures) Act 1986. Procedures were approved by the Animal Welfare and Ethical Review Bodies (AWERB) of the CRUK Manchester Institute and tumor volumes did not exceed the guidelines set by the Committee of the National Cancer Research Institute (Workman et al., 2010) as stipulated by the AWERB.

### Cancer Cell Lines

Cells were cultured under standard conditions and confirmed to be mycoplasma free. The *Braf*$^{V600E}$-driven 5555 melanoma cell line was established from the C57BL/6 *Braf* $^{+/LSL\text{-}V600E}$*Tyr*::*CreERT2* $^{+/o}$*p16* $^{INK4a-/-}$ model (Dhomen et al., 2009). CT26, 4T1, and MC38 cells are commercially available. *Ptgs1/Ptgs2*$^{-/-}$ (*Braf*$^{V600E}$-driven melanoma and 4T1) and *Ptgs2*$^{-/-}$ (MC38 and CT26) cells were generated by CRISPR/Cas9-mediated genome engineering as previously described (Zelenay et al., 2015). To restore COX-2 expression in *Ptgs2*$^{-/-}$ MC38 colorectal and *Ptgs1/Ptgs2*$^{-/-}$ melanoma cells, the complete open reading frame of murine *Ptgs2* was cloned from parental *Braf*$^{V600E}$-driven melanoma cell line into the retroviral vector pFB. The resulting construct was introduced in COX-deficient cells by standard retroviral transduction. Knockout of *Ptgs1, Ptgs2* and regain of COX-2 expression was verified by immunoblotting using anti COX-1 and COX-2 specific antibodies (Cell Signaling) and by monitoring the concentration of PGE2 in cell supernatants by ELISA (Cayman chemical).

### Mouse Procedures

Tumor cells were harvested by trypsinization, washed three times with PBS, filtered on a 70 µm cell strainer and injected subcutaneously into the flank of recipient mice. Growth profile experiments were performed injecting 1x10$^5$ cells in 100 µL of PBS. Tumor tissues analyzed at day 4 or 7 were harvested from mice injected with 2x10$^6$ cells in 100 µL of PBS. Importantly, we have confirmed that injection of 1x10$^6$ or 2x10$^6$ cells *Ptgs*$^{-/-}$ cells leads to the generation of tumors that are fully rejected into wild-type mice. Tumor cells were > 95% viable at the time of injection as determined by Trypan blue exclusion. Tumor size was quantified as the mean of the longest diameter and its perpendicular and expressed as tumor diameter. In depletion experiments, mice were injected one day before or from day 7 post-tumor cell implantation with 200 µg of specific Ab i.p. (control rat or mouse IgG, anti-Gr1 clone RB6-8C5, anti-NK1.1 clone PK136, anti-ASIALO GM-1, anti-CD4 clone GK1.5 and anti-CD8alpha clone YTS 169.4, all from BioXCell or Biolegend) and then every three days with 200 µg of the indicated antibody for the entire duration of the experiment. Depletion of neutrophils, NK cells, CD4$^+$ and CD8$^+$ T cells was confirmed by FACS using anti-CD49b-APC (clone DX5), anti-Ly6G-PE-CF594 (clone 1A8), anti CD4-Alexa700 (RM4-5) and anti-CD8a-PE (clone 53-6.7) respectively.

## METHOD DETAILS

### Quantitative RT-PCR

Tumors were collected and homogenized using TissueLyser II (QIAGEN) and total RNA extracted using RLT lysis buffer (QIAGEN) or PureZOL Reagent (BioRad) following the manufacturer's recommendations. RNA was further purified using RNeasy RNA isolation kit

(QIAGEN) or Direct-zol RNA Mini Prep Kit (Zymo Research). cDNA was synthesized using 1-3 μg of total RNA by reverse transcription using High Capacity cDNA archive kit (Applied Biosystems) and quantitative real-time PCR was performed using TaqMan probes (Applied Biosystems) using a QS5 fast real-time PCRsystem (Applied Biosystems) or the Biomark® HD system (FLUIDIGM). Data were analyzed with the $\Delta^2$CT method (Applied Biosystems, Real-Time PCR Applications Guide).

### FACS analysis

For analysis of tumor-infiltrating leukocytes, tumors were collected, cut into small pieces and digested with Collagenase IV (200 U/ml, Worthington Biochemical) and DNase I (0.2 mg/ml, Roche) for 35 minutes at 37°C, washed with FACS buffer (PBS containing 2% FCS, 2 mM EDTA and 0.01% sodium azide), filtered on a 70 μm cell strainer and pelleted. The composition of tumor infiltrate was determined by flow cytometry using a combination of the following antibodies: CD45-BV605 (Clone 30-F11), CD11b-BV785 (Clone M1/70), Ly6G-PE-CF594 (Clone 1A8), Ly6C-FITC (Clone AL-21), F4/80-PE-Cy7 (Clone CI: A3-1), anti-MHCII I-A/I-E-Alexa700 or APC-eFluor780 (Clone M5/114.15.2), anti-CD11c-PerCP/Cy5.5 (Clone N418), anti-CD103 APC or PE (Clone 2E7) NK1.1-APC or PE (Clone PK136); CD49b-APC (Clone DX5), XCR1-BV421 or Alexa647 (Clone ZET) Siglec-H-BV711 (Clone E50-2440), IFNγ-eFluor450 (Clone XMG1.2), CD44-APC-eFluor780 (Clone IM7), CD3e Percp-Cy5.5 or APC (Clone 145-2C11), CD8a-PE or -PE-Cy7 (Clone 53-6.7), CD4-FITC (Clone RM4-5) or CD4-APC-eFluor780 (Clone GK1.5) from eBioscience, BioLegend or BD Bioscience. Fc receptors were saturated with an anti-CD16/32 (clone 2.4G2, eBioscience) 5 minutes before the staining. Cell viability was determined by Aqua LIVE/Dead-405 nm staining (Invitrogen). Intracellular epitopes were detected after *ex vivo* restimulation (4h PMA/ionomycin) using Intracellular Fixation & Permeabilization Buffer set (eBioscience) following manufacturer instructions. Monensin (eBiolegend) and Brefeldin A (eBiolegend) solutions were added 2h before the staining. Live cell counts were calculated from the acquisition of a fixed number (5000) of 10 μm latex beads (Coulter) mixed with a known volume of unstained cell suspension. Cells were analyzed on a Fortessa X-20 (BD Bioscience) or on a Novocyte (ACEA).

### Immunofluorescence Analysis

Tumor tissues were mounted in OCT embedding medium (Thermo Scientific) and stored at −80°C. 30 μm consecutive sections were cut, mounted on Superfrost plus slides (Thermo Scientific) and fixed in 4% paraformaldehyde for 15 min, rehydrated in PBS and blocked in 5% normal goat or donkey (Sigma-Aldrich) serum, 2% BSA in PBS for 2h at room temperature (RT). Tumor sections were incubated with the following primary antibodies for 2h at RT or overnight at 4°C: affinity purified anti-Ly6G (Clone 1A8; BD Bioscience) and affinity purified anti-NK1.1-biotynilated (Clone PK136). Sections were then incubated for 1h at RT with the following species-specific cross-adsorbed detection antibodies: Alexa647-conjugated donkey anti-rat and FITC-conjugated streptavidin from Jackson ImmunoResearch Laboratories and Invitrogen-Molecular Probes, respectively. For DNA detection, DAPI (300 nM; Invitrogen-Molecular Probes) was used. After each step, sections were washed with PBS containing 0.01% (v/v) Tween 20 (VWR Chemicals) and finally mounted with antifade mounting medium FluorPreserve Reagent (Calbiochem) and analyzed with an Aperio VERSA 200 scanner (Leica). Negative controls were obtained by omission of the primary antibody. Cell number per high power field (HPF) was calculated using Fiji software version 2.0.0-rc14/1.49.

### NK cell *in vitro* killing and live tracking

NK cells used in *in vitro* assays were isolated from spleens of naive mice. Total splenocytes were stained using a biotinylated-antibody cocktail (anti-CD3e, anti-CD19, anti-CD4, anti-CD8a, anti-CD14, anti-Ly6G, anti-TER-119, anti-F4/80) for 15 min. After washing, cells were resuspended in 400 μL of MojoSortTM buffer (PBS, 2.5% w/v BSA, 10 mM EDTA), and 100 μL MojoSortTM streptavidin nanobeads for 15 min on ice. Afterward, cells were placed in a magnet and the supernatant isolated, followed by centrifugation and resuspension in complete RPMI. Isolated NK cells were plated in complete RPMI containing 1000 U/ml IL-2 at $10^6$ cells/ml for four days. Ten thousand *Ptgs*$^{+/+}$, *Ptgs*$^{-/-}$ or *Ptgs*$^{-/-}$ + COX-2 melanoma cells were plated in 384 well plates. Following adhesion for 2h, cells were stained in the wells with 0.5 μM CellTrace Calcein Green (ThermoFisher Scientific) for 20 min. NK cells were stained separately with 0.32 μM CellTrace Calcein Red-Orange (ThermoFisher Scientific) for 20 min. Cells were washed twice and 2500 NK cells were added to wells containing target cells. The plate was imaged using a Zeiss Axio Observer with a 20X air objective in a temperature-controlled chamber (37°C, 5% CO2). Images were captured using two LEDs: Cyan 470/24 (excitation: 461-487, dichroic: 461-487, emission: 499-530) and Green 550/15 (excitation: 543-566, dichroic: 543-566, emission: 580-611), with a 5 min interval for 6 hours. NK cell migration and target contact were analyzed using Fiji. Migration tracking was performed manually and migration parameters (speed, directionality, and displacement) were calculated using in-house developed MATLAB scripts. Contact analysis was performed by measuring the number of frames in which NK cells were in contact with their targets. A successful contact was defined as an NK cell actively interacting with a target for at least 3 consecutive frames. The researcher was blinded regarding the genotype of melanoma cells during analysis.

### Sorting of TAMs

Tumors were processed for FACS analysis as described above and stained with Aqua LIVE/DEAD-405 nm (Invitrogen), CD45-BV605 (Clone 30-F11), CD11b-BV785 (Clone M1/70), Ly6G-PE-CF594 (Clone 1A8), Ly6C-FITC (Clone AL-21), F4/80-PE-Cy7 (Clone CI: A3-1) and NK1.1-APC (Clone PK136) antibodies. FACS buffer used in these experiments was EDTA and sodium azide free. Live CD45$^+$ CD11b$^+$ F4/80$^+$ NK1.1$^-$ Ly6G$^-$ Ly6C$^{-/dim}$ cells from three *Ptgs*$^{+/+}$ and three *Ptgs*$^{-/-}$ tumor samples were sorted on a BD FACSAria III with > 98% purity. Each sample was a pool of 10 tumors.

### Adoptive NK cell transfer

One million splenic NK cells sorted from wild-type or *Ifng*$^{-/-}$ mice were injected intravenously into *Ifng*$^{-/-}$ mice 4 hours before and on day 3 post tumor cell injection. Briefly, NK cells were enriched using the Mojo NK cell isolation kit (Biolegend) according to manufacturer instructions. The resulting negative fraction was stained using Aqua LIVE/DEAD-405 nm (Invitrogen), CD19-eFluor450 (Clone eBio1D3), CD3e Percp-Cy5.5 (Clone 145-2C11), and NK1.1-APC (Clone PK136) antibodies and sorted on a BD FACSAria III with > 99% purity. Each preparation was a pool of five spleens.

### 3′-mRNA sequencing and analysis

RNA was prepared from sorted TAMs or bulk tumors as described above. Lexogen QuantSeq 3′-mRNaseq Library Prep Kit (FWD, Cat. No. 015) for Illumina was used for the construction of sequencing libraries from 500ng (bulk tumors) or 800pg (sorted TAMs) of total RNA, and then sequenced in Illumina NextSeq500 at the CRUK Manchester Institute Molecular Biology Core facility. The fastq read files for the mouse QuantSeq sequencing were trimmed with BBDUK from the BBMAP tools software, to remove the first 12 bases of each read and to remove contaminates as suggested by the lexogen documentation. Trailing polyG and polyA tails were removed with *fqtrim* and *cutadapt*. After trimming the reads were aligned to the mouse GRCm38 genome reference downloaded from ENSEMBL with STAR (Dobin et al., 2013). The aligned reads were then allocated to genetic features in the GRCm38 v86 annotation using *featureCounts* from the *Subread* software (Liao et al., 2014). The Feature Counts count matrix was then read into the R bioconductor package edgeR (Robinson et al., 2010), and $\log_2$(CPM+1) values were used to generate heatmaps. The differential expression analysis was performed using the edgeR package with TMM normalization and the generalized linear model differential expression test. Differentially expressed genes were defined based on a false discovery rate (FDR) $\leq$ 0.05 and a $\text{Log}_2$ fold change (FC) ± 1.5. The resulting gene list was analyzed with Ingenuity Pathways Analysis software (IPA) (Krämer et al., 2014). Results of the IPA analysis are shown in Figure S5B and in Table S4.

### Single cell mRNA sequencing and analysis

Tumors were processed for FACS analysis as described above and stained with Aqua LIVE/DEAD-405 nm (Invitrogen) and CD45-BV605 (Clone 30-F11) antibodies. FACS buffer used in these experiments was EDTA and sodium azide free. Live CD45+ cells from ten pooled tumors per each group were sorted on a BD FACSAria III with a purity > 98%. Cells were counted using a hemocytometer after Trypan Blue exclusion. Sixteen thousand cells were loaded into a channel of a Chromium Chip B (10X Genomics, PN-1000073) and GEMs were generated on the Chromium Controller (10X Genomics, GCG-SR-1). 3.5% spike-in parental *Braf*$^{V600E}$-driven melanoma cells were added to each sample for assessing sample-to-sample variability. Indexed sequencing libraries were prepared using the Chromium Single Cell 3′ Reagent Kit v3 (10X Genomics, PN-1000075, PN-120262) according to manufacturer's instructions, with 11 cycles of cDNA amplification and 14 cycles of sample index PCR.

Libraries were quantified by qPCR using a Kapa Library Quantification Kit for Illumina sequencing platforms (Kapa Biosystems Inc. Cat No: KK4873). Paired-end sequencing was carried out by clustering 1.4 pM of the equimolar pooled libraries on a NextSeq 500 sequencer (Illumina inc.). Raw sequencing data were converted into fastq files using the Cell Ranger Pipeline version 3.1 (10X Genomics). The resulting filtered feature bc matrix in h5 format was loaded on Partek Flow software for analysis. Briefly, cells were filtered for total reads (min:500-max:15,000), detected genes (min:200-max:4,000) and mitochondrial read contents (min:0%-max:10%). Resulting features were normalized using $\text{Log}_2$ CPM+1. Genes with expression equal to zero in 100% of the cells were filtered out. A principal component analysis was run in order to identify common sources of variation between the two datasets. Finally, the resulting data were subjected to graph-based clustering analysis using the first 15 principal components. The identified clusters were visualized using t-distributed Stochastic Neighbor Embedding of the principal components (t-SNE) and different immune cell populations were classified based on group specific biomarkers (Table S1) as shown in Figure 3E. Gene set enrichment analysis (GSEA) examining enriched Hallmark gene sets (Liberzon et al., 2015) in the same cell cluster from *Ptgs*$^{-/-}$ or *Ptgs*$^{-/-}$-NK cell depleted tumors was performed using GenePattern platform (Reich et al., 2006). To run single-sample GSEA (ssGSEA), gene expression dataset files (.gct file), immune marker gene set file (.gmt file) and class parameter (.cls file) were uploaded on GenePattern environment and the ssGSEA score for each hallmark gene set was calculated using default parameters (Figure 3G; Table S3).

### Bioinformatic Analysis of Patient Datasets

TCGA gene expression data (mRNA level 3 RSEM normalized) was downloaded from the Broad Institute Firehose portal between September 2017 and February 2018. Datasets with less than 100 samples were not included in the analysis. Tumor tissue and normal tissue samples (sample ID ending in 11 or 12) were separated. METABRIC data was downloaded from cBioPortal. Patients with gene expression data were classified into three groups (HER2 positive, ER/PR status positive/HER2 negative, ER/PR/HER2 negative).

To obtain the COX-IS, signature scores were computed by mean expression ($\text{Log}_2$ RSEM) of signature genes, as well as by mean Z-score normalized gene expression values. The 'cancer-promoting' and 'cancer-inhibitory' inflammatory genes whose expression was regulated by COX-2 activity in the mouse models (Figure 3C; Zelenay et al., 2015) were computed as follows: *VEGFA, CCL2, IL8, CXCL1, CXCL2, CSF3, IL6, IL1B* and *IL1A* were positively correlated (*pos,* also referred as CP) and expressed as:

$$pos = \sum_{i=1}^{n_p} G_i^{pos}(e),$$

CCL5, CXCL9, CXCL10, CXCL11, IL12A, IL12B, IFNG, CD8A, CD8B, GZMA, GZMB, EOMES, PRF1, STAT1 and TBX21 were negatively correlated (neg, also referred ad CI) and defined as:

$$neg = \sum_{i=1}^{n_n} G_i^{neg}(e),$$

where $n_p$ and $n_n$ are the number of genes in *pos* and *neg* groups respectively.

Finally, COX-IS was calculated as:

$$\mathrm{COX-IS} = \frac{\frac{1}{n_p}\sum_{i=1}^{n_p} G_i^{pos}(e)}{\frac{1}{n_n}\sum_{i=1}^{n_n} G_i^{neg}(e)}$$

We computed pairwise Pearson correlation coefficients for genes of the COX-IS in each patient cohort, and plotted coefficients against *PTGS2* in each cohort. Tumor types were ordered left to right by the increasing mean correlation of *PTGS2* with genes of the CI signature. For HNSC and LUAD datasets, we plotted the Pearson correlation coefficient against the $-\mathrm{Log}_{10}$ p value from the same analysis. The expression of CI genes in NK cell-high versus -low patients was analyzed. Patients in the top 25% and bottom 25% of NK cell infiltration (as defined by Danaher et al., 2017) were classified as NK cell high and low respectively. Pearson correlation coefficients were also computed for the *PTGS2* gene against MCPcounter (version 1.1.0) (Becht et al., 2016) and (Danaher et al., 2017) cell populations. Heatmaps were generated using the *pheatmap* (version 1.0.12) package. Kaplan-Meier plots for overall survival were generated at the maximum follow up threshold per each tumor type and the COX-IS was used to segregate high risk from low risk patients. Patient from LUAD (n = 512), HNSC (n = 517), TNBC (n = 251), MSKCM (n = 357) and CESC (n = 305) were stratified in quartiles (75%, 50% and 25% stringency) according to the COX-IS. The most significant comparison is shown. All survival analysis was carried out using the R survival (version 3.1-8) package, and plotted using GraphPad Prism. For selected gene signatures (see Table S5) univariate survival analysis was carried out by stratifying patients into high and low groups with three different cut-offs: 25%, 50% and 75%. For each signature the optimal cut-off, defined by lowest logrank p value, was selected. We utilized the overall survival end-point data annotated by Thorsson et al., 2018 and expressed this as years. Multivariate analysis was carried out for COX-IS using clinical data downloaded from cBioPortal, we defined age, sex and stage variables for each patient where appropriate. Staging was converted to a continuous variable, grouping together all Stage I ( = 1), Stage II ( = 2), Stage III ( = 3) and Stage IV ( = 4). There were 488 LUAD patients with the required information (176 events). For HNSC, we also adjusted for HPV status (n = 275 with sufficient information, 137 events). For M-SKCM, 9 patients annotated as Stage I/II NOS were given a stage score of 1. Overall there were 314 patients (167 events). For CESC, we adjusted for age and stage (n = 301, events = 74). For KIRC, we adjusted for age, stage and sex (n = 515, events = 168). For OV, we adjusted for age and stage (n = 302, events = 183). The TNBC cohort from METABRIC, was adjusted for age, tumor stage, chemotherapy (CTx), radiotherapy (RTx) and grade (n = 177, events = 83).

Datasets of cancer patients receiving ICB were obtained following instructions within individual publications. For the renal cancer cohort (McDermott et al., 2018) we obtained special permissions and access to data through a written agreement between The CRUK Cancer Inflammation and Immunity Group and Roche/Genentech. All datasets that required counts to be processed were computed using edgeR (version 3.24.3). Genes were filtered out based on a threshold of 0.25 CPM in 10% samples. Log2 CPM+1 expression matrices were generated and used for downstream analysis. For the McDermott cohort, fastq files were downloaded from the European Genome Archive under accession EGAS00001002928. For the Kim and Gide cohorts, fastq files were obtained from the European Nucleotide Archive under accessions PRJEB25780 and PRJEB23709 respectively. Data for the bladder cancer cohort (Mariathasan et al., 2018) were obtained using the IMVigor210Biologies R package. A counts matrix for the Snyder et al., 2017 cohort was available to download from *zenodo.org* following the link in the online version of the paper. One Nanostring dataset was obtained from the Chen et al., 2016 publication. This dataset was downloaded from the supplementary file of the study, and included a main cohort plus an extra cohort of 8 pre-PD1 patients (Roh et al., 2017) that we also included in our analysis. FPKM values for the Hugo and Riaz datasets were obtained from the GEO database under accessions GSE78220 and GSE91061 respectively. The Van Allen cohort TPM matrix was downloaded from http://github.com/vanallenlab. In agreement with the original studies, patients from Mariathasan, McDermott, and Snyder cohorts, with progressive disease (PD) and stable disease (SD) were pooled as non-responders, and those with a partial or complete response were pooled as responders. For melanoma cohorts and the gastric cohort, stable disease patients were pooled instead with responders.

Logistic regression models were constructed using the *glm* function in R. Nested and individual models were assessed by the Chi-square test. All available patients were used where appropriate, except for TMB models in the Mariathasan cohort, where 76 patients did not have mutation data. PDL1 expression, labeled as enrolment IC (immune cell) level, was used as a categorical variable. On-treatment samples were excluded from all datasets where available. RNA sequencing datasets from pooled melanoma cohorts (Gide et al., 2019; Hugo et al., 2016; Riaz et al., 2017; Van Allen et al., 2015) and bladder cancer (Mariathasan et al., 2018; Snyder et al., 2017) were analyzed to determine the association of the COX-IS with survival.

## QUANTIFICATION AND STATISTICAL ANALYSIS

For all studies, sample size was defined on the basis of past experience on cancer models, to detect differences of 20% or greater between the groups (10% significance level and 80% power). Values were expressed as mean ± SEM or median of biological

CellPress
OPEN ACCESS

replicates, as specified. Unpaired Student's t test, Pearson's correlation, chi-square test, one- and two-way ANOVA were used as specified. Tukey, Dunnet or Sidak corrections were applied for multiple comparisons. A Mann–Whitney *U*-test was used in cases of non-Gaussian distribution. Survival curves and hazard ratio were calculated with a Log-rank (Mantel-Cox) test. A ROUT test (Q = 0.05 stringency) was applied to exclude outliers. A p value < 0.05 (*p < 0.05, **p < 0.01, ***p < 0.001) was considered significant. Statistics were calculated with GraphPad Prism version 8.2.0 (GraphPad Software). Flow cytometry standard (.fcs) files were analyzed using FlowJo (FlowJo LLC.) version 10.4.2 and Cytobank (CytoBank, Inc.) software. Partek Flow software (Single cell Toolkit, build version 8.0.19.0610) was used for single cell RNA-sequencing analysis. R software (R project) was used for analysis of cancer patient datasets.

Immunity, Volume 53

# Supplemental Information

# Antagonistic Inflammatory Phenotypes
# Dictate Tumor Fate and Response
# to Immune Checkpoint Blockade

**Eduardo Bonavita, Christian P. Bromley, Gustav Jonsson, Victoria S. Pelly, Sudhakar Sahoo, Katherine Walwyn-Brown, Sofia Mensurado, Agrin Moeini, Eimear Flanagan, Charlotte R. Bell, Shih-Chieh Chiang, C.P. Chikkanna-Gowda, Neil Rogers, Bruno Silva-Santos, Sebastien Jaillon, Alberto Mantovani, Caetano Reis e Sousa, Nadia Guerra, Daniel M. Davis, and Santiago Zelenay**

Figure S1

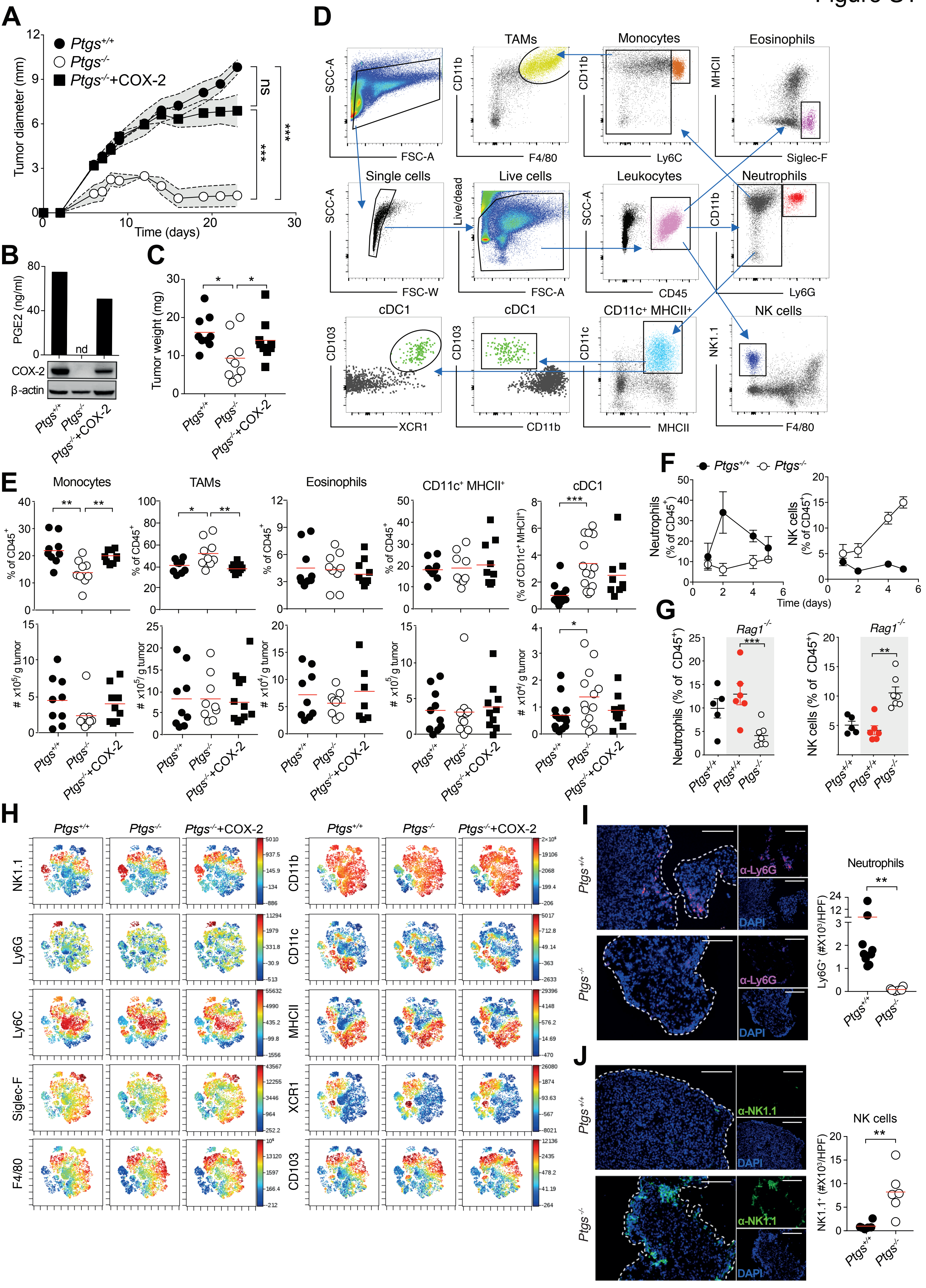

A
Ptgs+/+
Ptgs-/-
Ptgs-/-+COX-2
Tumor diameter (mm)
Time (days)
ns
***
B
PGE2 (ng/ml)
nd
COX-2
β-actin
C
Tumor weight (mg)
D
TAMs
Monocytes
Eosinophils
Single cells
Live cells
Leukocytes
Neutrophils
cDC1
CD11c+ MHCII+
NK cells
SCC-A
FSC-A
CD11b
F4/80
Ly6C
MHCII
Siglec-F
FSC-W
Live/dead
CD45
Ly6G
CD103
XCR1
CD11c
NK1.1
E
Monocytes
TAMs
Eosinophils
CD11c+ MHCII+
cDC1
% of CD45+
(% of CD11c+ MHCII+)
# x10^5/g tumor
# x10^4/g tumor
F
Neutrophils (% of CD45+)
NK cells (% of CD45+)
Time (days)
G
Rag1-/-
Neutrophils (% of CD45+)
NK cells (% of CD45+)
H
CD11b
CD11c
MHCII
XCR1
CD103
NK1.1
Ly6G
Ly6C
Siglec-F
F4/80
I
α-Ly6G
DAPI
Neutrophils
Ly6G+ (#X10^3/HPF)
J
α-NK1.1
NK cells
NK1.1+ (#X10^3/HPF)

**Figure S1. (Related to Figure 1) Genetic ablation of cancer cell-intrinsic COX alters the intratumoral accumulation of select innate immune cells.** (A) Growth profile of tumors following implantation of *Ptgs*$^{+/+}$, *Ptgs*$^{-/-}$ and *Ptgs*$^{-/-}$ + COX-2 melanoma cells into wild-type mice. (B) PGE2 production and COX-2 expression by cells used in A. (C) Weight of *Ptgs*$^{+/+}$, *Ptgs*$^{-/-}$ and *Ptgs*$^{-/-}$ + COX-2 tumors four days after melanoma cell implantation. (D) Representative gating strategy. (E) Immune cell infiltrate analysis of tumors formed by *Ptgs*$^{+/+}$, *Ptgs*$^{-/-}$ and *Ptgs*$^{-/-}$ + COX-2 melanoma cells four days post-implantation. The frequency and the number of intratumoral monocytes, TAMs, eosinophils, CD11c$^{+}$ MHCII$^{+}$ cells and cDC1s are shown. (F) Kinetics of neutrophil and NK cell accumulation in *Ptgs*$^{+/+}$ and *Ptgs*$^{-/-}$ tumors. (G) Frequency of infiltrating leukocytes one-month post-cancer cell implantation in wild-type (injected with *Ptgs*$^{+/+}$ cells) *or Rag1*$^{-/-}$ (injected with either *Ptgs*$^{+/+}$ or *Ptgs*$^{-/-}$ cells). Neutrophils and NK cells are gated on live CD45$^{+}$ cells. (H) t-SNE projections of individual markers used in Figure 1A. Each plot is obtained from six concatenated samples. (I-J) Immunofluorescence analysis of neutrophils (I) and NK cells (J) in tumors formed by *Ptgs*$^{+/+}$ and *Ptgs*$^{-/-}$ melanoma cells four days post implantation (scale bar 100 μm). Data are expressed as mean ± SEM, two-way ANOVA (A), one-way ANOVA (C, E, G), unpaired Student's t test (I-J).

Figure S2

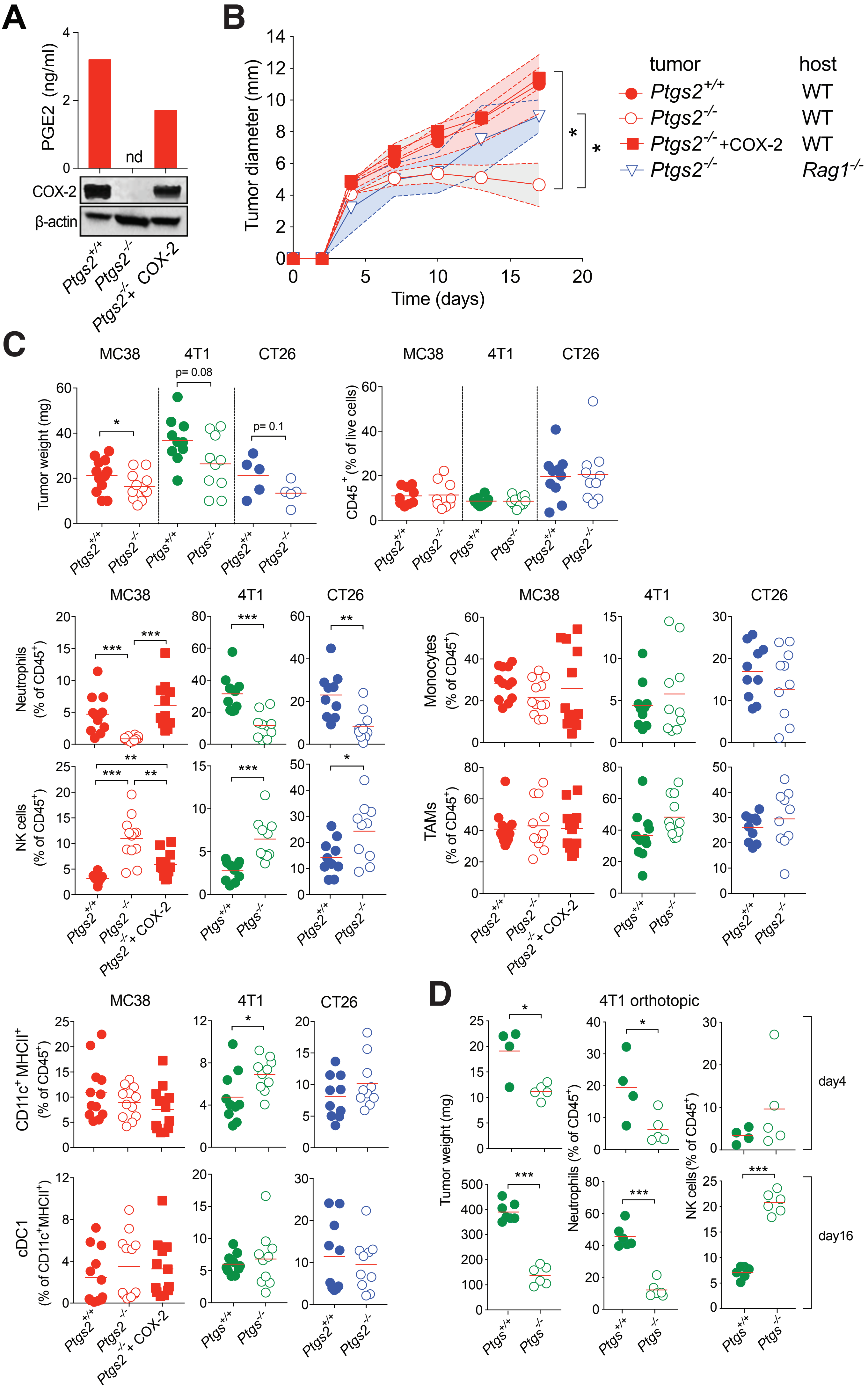
A
B
C
D
PGE2 (ng/ml)
nd
COX-2
β-actin
Ptgs2+/+
Ptgs2-/-
Ptgs2-/- + COX-2
Tumor diameter (mm)
Time (days)
tumor
host
Ptgs2+/+ WT
Ptgs2-/- WT
Ptgs2-/- +COX-2 WT
Ptgs2-/- Rag1-/-
MC38
4T1
CT26
Tumor weight (mg)
p= 0.08
p= 0.1
CD45+ (% of live cells)
Neutrophils (% of CD45+)
NK cells (% of CD45+)
Monocytes (% of CD45+)
TAMs (% of CD45+)
CD11c+ MHCII+ (% of CD45+)
cDC1 (% of CD11c+MHCII+)
4T1 orthotopic
Neutrophils (% of CD45+)
NK cells (% of CD45+)
day4
day16

**Figure S2. (Related to Figure 1) Neutrophil and NK cell accumulation within the TME is regulated by cancer cell-intrinsic COX activity in various cancer models.** (A) PGE2 production and COX-2 expression by *Ptgs2*$^{+/+}$, *Ptgs2*$^{-/-}$ and *Ptgs2*$^{-/-}$ + COX-2 MC38 colorectal cancer cells. (B) Growth profile of *Ptgs2*$^{+/+}$, *Ptgs2*$^{-/-}$ and *Ptgs2*$^{-/-}$ + COX-2 MC38 cancer cells in wild-type and *Rag1*$^{-/-}$ mice. (C) Weight of tumors formed by *Ptgs2*$^{+/+}$ *or Ptgs2*$^{-/-}$ MC38 colorectal, 4T1 (*Ptgs*$^{+/+}$ and *Ptgs* $^{-/-}$) breast and CT26 colorectal cells four days post-implantation. Immune cell infiltrate analysis of tumors formed by *Ptgs2*$^{+/+}$, *Ptgs2*$^{-/-}$ and *Ptgs2*$^{-/-}$ + COX-2 MC38 and CT26 colorectal and *Ptgs*$^{+/+}$ and *Ptgs*$^{-/-}$ 4T1 breast cancer cells four days post-implantation. The frequency of CD45$^{+}$ cells, neutrophils, NK cells, monocytes, TAMs, CD11c$^{+}$ MHCII$^{+}$ cells and cDC1s is shown. (D) Tumor weight, NK cell and neutrophil frequencies in *Ptgs*$^{+/+}$and *Ptgs*$^{-/-}$ orthotopic 4T1 tumors on days 4 and 16 post injection. Data are expressed as mean ± SEM, two-way ANOVA (B), one-way ANOVA or unpaired Student’s t test (C-D).

Figure S3

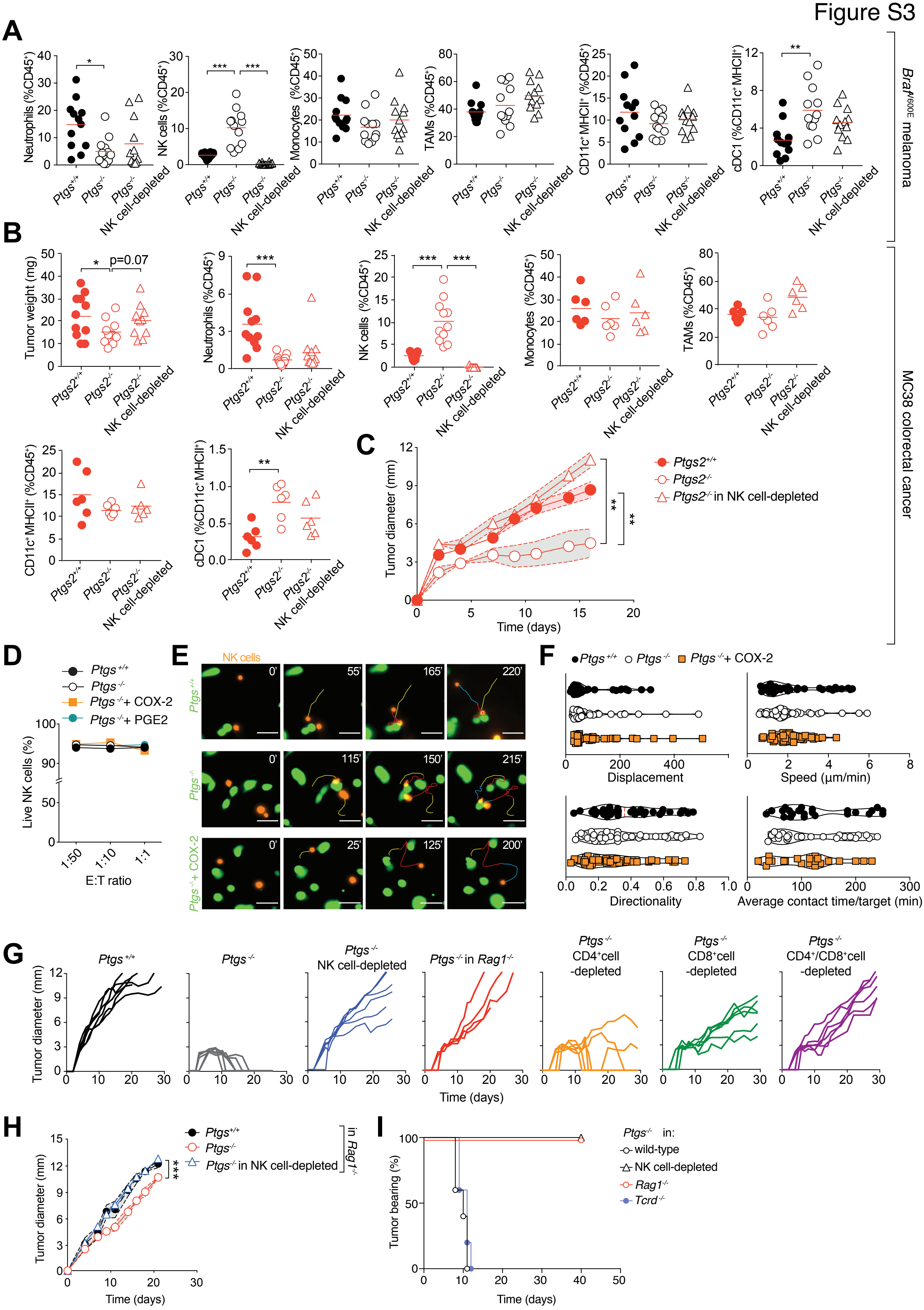
A
B
C
D
E
F
G
H
I
Braf V600E melanoma
MC38 colorectal cancer
Neutrophils (%CD45+)
NK cells (%CD45+)
Monocytes (%CD45+)
TAMs (%CD45+)
CD11c+ MHCII+ (%CD45+)
cDC1 (%CD11c+ MHCII+)
Tumor weight (mg)
p=0.07
Ptgs+/+
Ptgs-/-
Ptgs-/- NK cell-depleted
Ptgs2+/+
Ptgs2-/-
Ptgs2-/- NK cell-depleted
Ptgs2-/- in NK cell-depleted
Tumor diameter (mm)
Time (days)
Ptgs-/- + COX-2
Ptgs-/- + PGE2
Live NK cells (%)
E:T ratio
NK cells
Displacement
Speed (µm/min)
Directionality
Average contact time/target (min)
Ptgs-/- in Rag1-/-
Ptgs-/- CD4+cell -depleted
Ptgs-/- CD8+cell -depleted
Ptgs-/- CD4+/CD8+cell -depleted
Ptgs-/- in NK cell-depleted
in Rag1-/-
Tumor bearing (%)
Ptgs-/- in:
wild-type
NK cell-depleted
Rag1-/-
Tcrd-/-

**Figure S3. (Related to Figure 1 and Figure 2) NK cells are essential for both T cell-independent and T cell-dependent tumor control.** (A) Neutrophil, NK cell, monocyte, CD11c$^{+}$ MHCII$^{+}$ cell, TAM and cDC1 frequency in *Ptgs*$^{+/+}$ and *Ptgs*$^{-/-}$ tumors four days post-implantation in NK cell-competent or depleted mice. (B) Tumor weight and immune cell infiltrate in *Ptgs2*$^{+/+}$ and *Ptgs2*$^{-/-}$ MC38 tumors four days after cell implantation in NK cell-competent or depleted mice. (C) Growth profile of *Ptgs2*$^{+/+}$ and *Ptgs2*$^{-/-}$ MC38 colorectal cancer cells implanted into NK cell-competent or -depleted mice. Data are expressed as mean ± SEM one-way (A-B) or two-way ANOVA (C). (D) Percentage of viable NK cells after the killing assay performed in coculture with *Ptgs*$^{+/+}$, *Ptgs*$^{-/-}$ (+/- synthetic PGE2) and *Ptgs*$^{-/-}$ + COX-2 melanoma cells. (E) Migration tracking of NK cells in co-cultures with *Ptgs*$^{+/+}$, *Ptgs*$^{-/-}$ or *Ptgs2*$^{-/-}$ + COX-2 melanoma cells (scale bar 50 µm, time expressed in minutes). (F) Migration parameters of NK cells co-cultured with *Ptgs*$^{+/+}$, *Ptgs*$^{-/-}$ or *Ptgs2*$^{-/-}$ + COX-2 targets showing the average contact time between NK cells and target cells, displacement, speed and directionality. (G) Individual growth profiles of *Ptgs*$^{+/+}$ and *Ptgs*$^{-/-}$ melanoma cells implanted into *Rag1*$^{-/-}$ or wild-type mice untreated or depleted of NK, CD4$^{+}$ and/or CD8$^{+}$ cells shown in Figure 2A. (H) Growth profile of *Ptgs*$^{+/+}$ and *Ptgs*$^{-/-}$ tumors in *Rag1*$^{-/-}$ or *Ptgs*$^{-/-}$ tumors in *Rag1*$^{-/-}$ depleted of NK cells, two-way ANOVA. (I) Kaplan-Meier plots showing the fraction of tumor- bearing wild-type (n=5), *Rag1*$^{-/-}$ (n=5), NK cell-depleted (n=11) and *Tcrd*$^{-/-}$ (n=5) mice injected with *Ptgs*$^{-/-}$ melanoma cells.

Figure S4

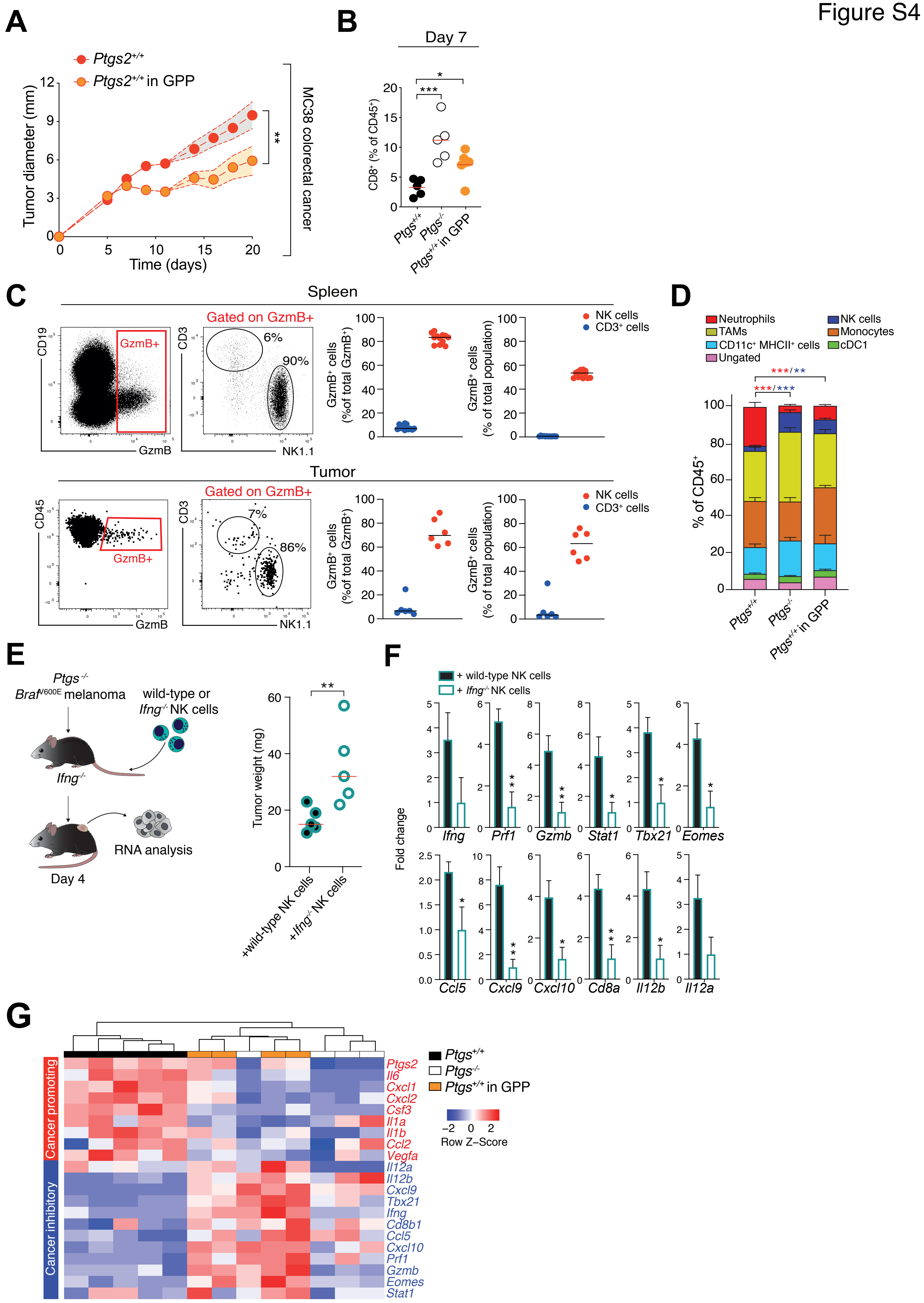
A
Ptgs2+/+
Ptgs2+/+ in GPP
Tumor diameter (mm)
Time (days)
MC38 colorectal cancer
**
B
Day 7
CD8+ (% of CD45+)
Ptgs+/+
Ptgs-/-
Ptgs+/+ in GPP
C
Spleen
Gated on GzmB+
GzmB+
CD19
GzmB
CD3
NK1.1
6%
90%
GzmB+ cells (%of total GzmB+)
GzmB+ cells (% of total population)
NK cells
CD3+ cells
Tumor
CD45
7%
86%
D
Neutrophils
TAMs
CD11c+ MHCII+ cells
Ungated
NK cells
Monocytes
cDC1
% of CD45+
Ptgs+/+
Ptgs-/-
Ptgs+/+ in GPP
E
Ptgs-/-
BrafV600E melanoma
wild-type or
Ifng-/- NK cells
Ifng-/-
Day 4
RNA analysis
Tumor weight (mg)
+wild-type NK cells
+Ifng-/- NK cells
F
+ wild-type NK cells
+ Ifng-/- NK cells
Fold change
Ifng
Prf1
Gzmb
Stat1
Tbx21
Eomes
Ccl5
Cxcl9
Cxcl10
Cd8a
Il12b
Il12a
G
Cancer promoting
Cancer inhibitory
Ptgs2
Il6
Cxcl1
Cxcl2
Csf3
Il1a
Il1b
Ccl2
Vegfa
Il12a
Il12b
Cxcl9
Tbx21
Ifng
Cd8b1
Ccl5
Cxcl10
Prf1
Gzmb
Eomes
Stat1
Ptgs+/+
Ptgs-/-
Ptgs+/+ in GPP
Row Z-Score

**Figure S4. (Related to Figure 2 and Figure 3) Dual ablation of EP2 and EP4 on NK cells unleashed tumor control and the inflammatory TME switch in COX-competent tumors.** (A) Growth profile of *Ptgs2*$^{+/+}$ MC38 colorectal cancer cells in wild-type and GPP mice. (B) Frequency of CD8$^{+}$ T cells gated on live, CD45$^{+}$, CD3ε$^{+}$ cells in *Ptgs*$^{+/+}$ and *Ptgs*$^{-/-}$ tumors in wild-type or *Ptgs*$^{+/+}$ tumors in GPP mice analyzed seven days post-implantation. (C) Representative plots and frequency of NK1.1$^{+}$ and CD3ε$^{+}$ cells within total GzmB$^{+}$ cells and frequency of GzmB$^{+}$ NK1.1$^{+}$ and GzmB$^{+}$ CD3ε$^{+}$ cells in spleen or within *Ptgs*$^{+/+}$ tumors in naïve or in wild-type mice four days post-implantation, respectively. (D) Percentage of innate immune cell populations in *Ptgs*$^{+/+}$ and *Ptgs*$^{-/-}$ tumors in wild-type or *Ptgs*$^{+/+}$ tumors in GPP mice. (E) Weight of *Ptgs*$^{-/-}$ tumors four days post-implantation in *Ifng*$^{-/-}$ mice adoptively transferred with wild-type or *Ifng*$^{-/-}$ NK cells. (F) Expression levels of CI genes in *Ptgs*$^{-/-}$ tumors from *Ifng*$^{-/-}$ mice infused with either wild-type (n=5) or *Ifng*$^{-/-}$ (n=5) NK cells. Data are normalized to *Gapdh* and expressed as mean ± SEM of the fold change of the average expression in tumors from mice transferred with *Ifng*$^{-/-}$ NK cells. (G) Gene expression of factors associated with CP (red) or CI (blue) inflammation normalized to *Gapdh* in tumors from *Ptgs*$^{+/+}$ and *Ptgs*$^{-/-}$ wild-type or *Ptgs*$^{+/+}$ in GPP mice. Two-way ANOVA (A), one-way ANOVA (B, D), unpaired Student’s t test (E, F).

# Figure S5

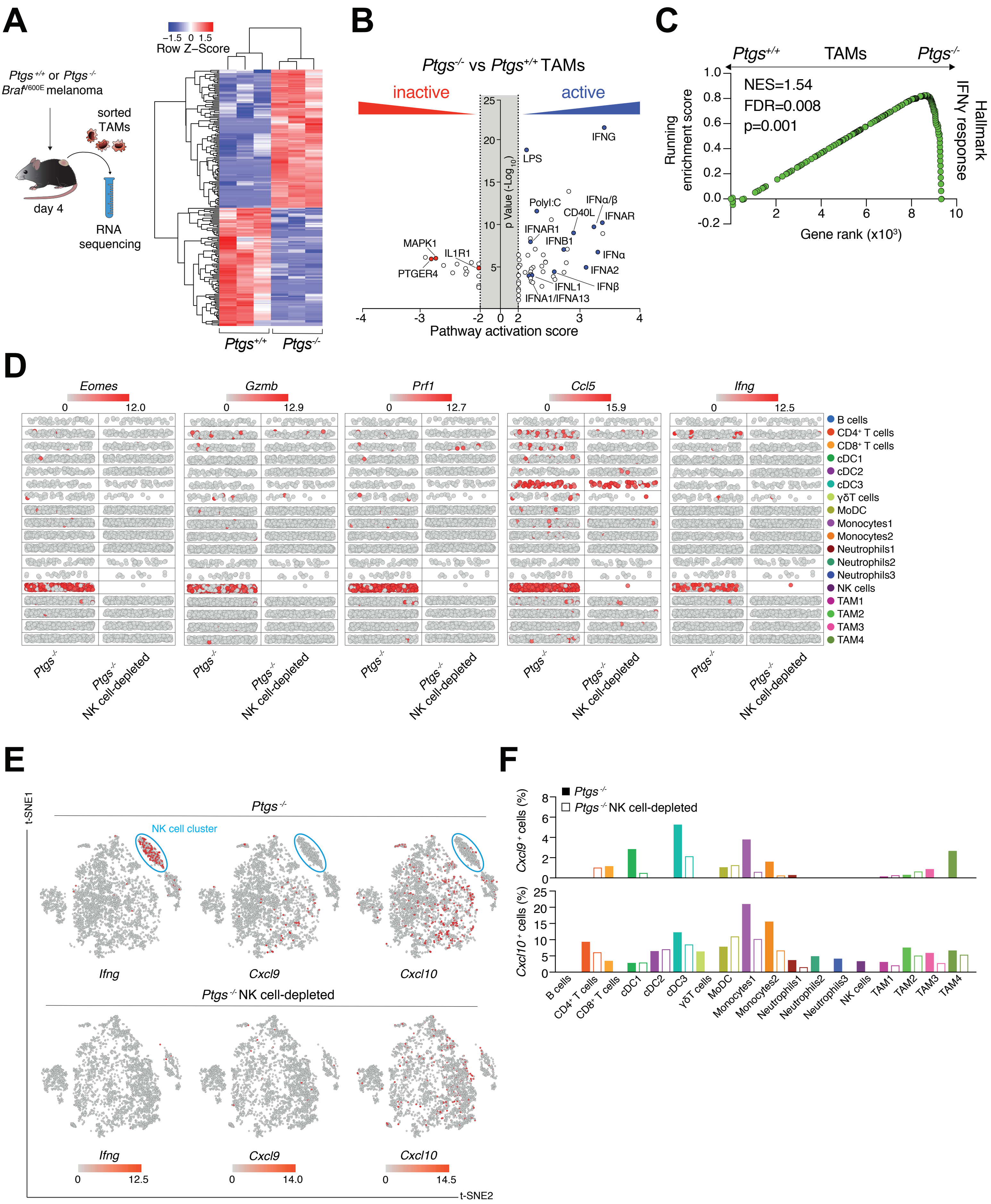

A
Ptgs+/+ or Ptgs-/-
BrafV600E melanoma
sorted TAMs
day 4
RNA sequencing
-1.5 0 1.5
Row Z-Score
Ptgs+/+
Ptgs-/-
B
Ptgs-/- vs Ptgs+/+ TAMs
inactive
active
p Value (-Log10)
Pathway activation score
IFNG
LPS
PolyI:C
IFNα/β
CD40L
IFNAR
IFNAR1
IFNB1
IFNα
IFNA2
IFNβ
IFNL1
IFNA1/IFNA13
MAPK1
IL1R1
PTGER4
C
Ptgs+/+
TAMs
Ptgs-/-
NES=1.54
FDR=0.008
p=0.001
Running enrichment score
Gene rank (x10³)
Hallmark IFNγ response
D
Eomes
Gzmb
Prf1
Ccl5
Ifng
Ptgs-/-
Ptgs-/- NK cell-depleted
B cells
CD4+ T cells
CD8+ T cells
cDC1
cDC2
cDC3
γδT cells
MoDC
Monocytes1
Monocytes2
Neutrophils1
Neutrophils2
Neutrophils3
NK cells
TAM1
TAM2
TAM3
TAM4
E
t-SNE1
t-SNE2
Ptgs-/-
NK cell cluster
Ifng
Cxcl9
Cxcl10
Ptgs-/- NK cell-depleted
F
Ptgs-/-
Ptgs-/- NK cell-depleted
Cxcl9+ cells (%)
Cxcl10+ cells (%)

**Figure S5. (Related to Figure 3 and Figure 4) Bulk and single-cell RNA sequencing of tumors reveal widespread NK cell-dependent upregulation of an IFNγ-response.** (A) Analysis by RNAseq of TAMs sorted from tumors formed by *Ptgs*$^{+/+}$ and *Ptgs*$^{-/-}$ melanoma cells four days post-implantation, each replicate is a pool of ten tumors. The heatmap shows z-scores for transcript levels of the differentially expressed genes (FDR≤0.05; $Log_2FC \geq 1.5$). (B) Upstream regulators predicted by Ingenuity Pathway Analysis of differentially expressed genes between TAMs isolated from *Ptgs*$^{+/+}$ versus *Ptgs*$^{-/-}$ tumors. (C) GSEA of hallmark IFNγ response gene set in TAMs from *Ptgs*$^{-/-}$ compared to *Ptgs*$^{+/+}$ tumors. FDR calculated using GSEA. (D) Dot-plot showing *Eomes*, *Gzmb*, *Prf1*, *Ccl5* and *Ifng* expression (normalized CPM) in the individual cell clusters from single cell-RNA sequencing of untreated or NK cell-depleted *Ptgs*$^{-/-}$ tumors. (E) t-SNE projections showing comparison of *Ifng*, *Cxcl9* and *Cxcl10* gene expression in the tumor immune infiltrate from *Ptgs*$^{-/-}$ and *Ptgs*$^{-/-}$ NK cell-depleted mice (F). *Cxcl9* and *Cxcl10* gene expression analysis in all cell clusters shown in Figure 4A. Data are expressed as fraction of cells expressing the indicated gene per individual cell cluster.

Figure S6

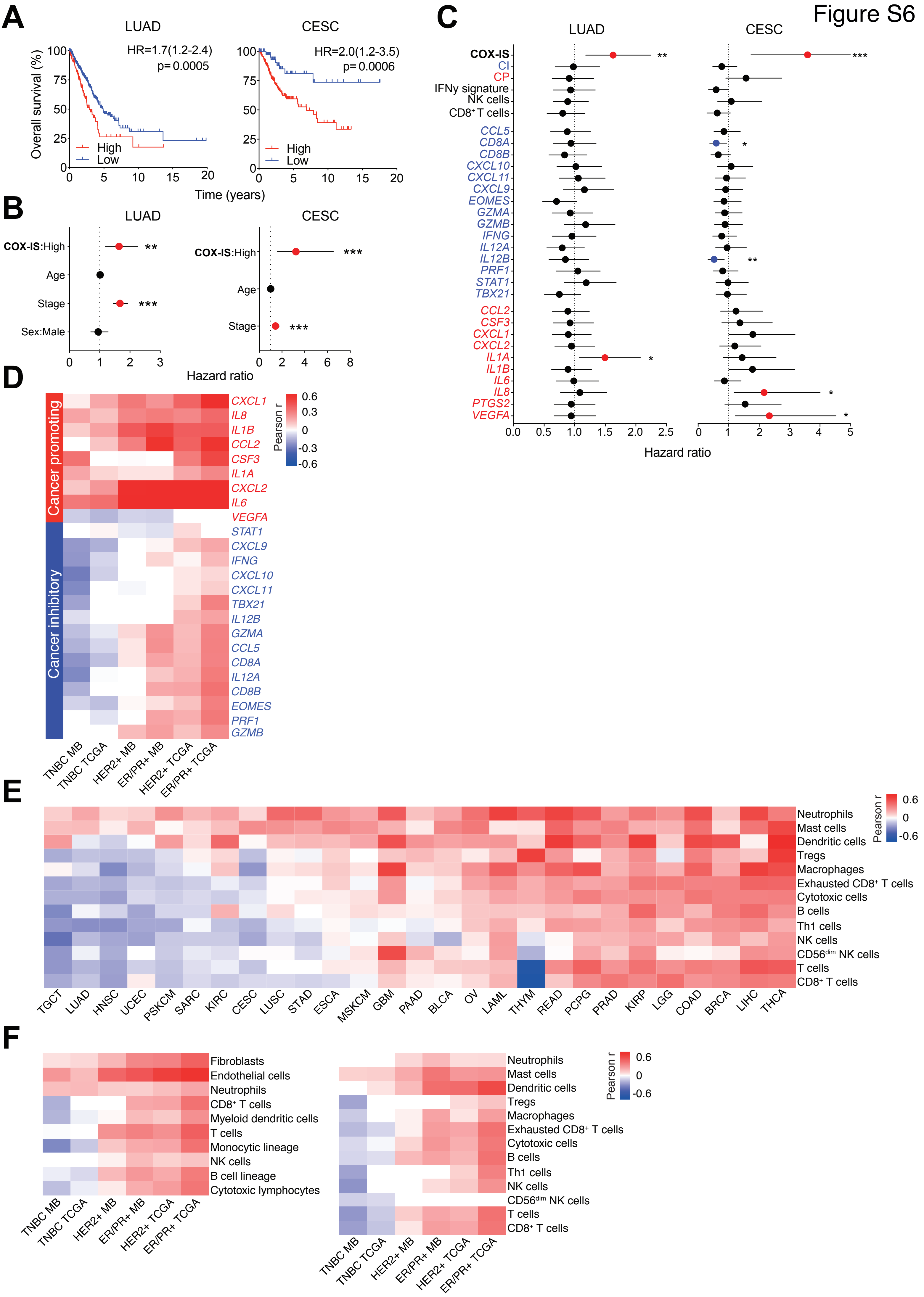
A
LUAD
HR=1.7(1.2-2.4)
p= 0.0005
CESC
HR=2.0(1.2-3.5)
p= 0.0006
Overall survival (%)
High
Low
Time (years)
B
LUAD
CESC
COX-IS:High
Age
Stage
Sex:Male
Hazard ratio
C
LUAD
CESC
COX-IS
CI
CP
IFNy signature
NK cells
CD8+ T cells
CCL5
CD8A
CD8B
CXCL10
CXCL11
CXCL9
EOMES
GZMA
GZMB
IFNG
IL12A
IL12B
PRF1
STAT1
TBX21
CCL2
CSF3
CXCL1
CXCL2
IL1A
IL1B
IL6
IL8
PTGS2
VEGFA
Hazard ratio
D
Cancer promoting
Cancer inhibitory
CXCL1
IL8
IL1B
CCL2
CSF3
IL1A
CXCL2
IL6
VEGFA
STAT1
CXCL9
IFNG
CXCL10
CXCL11
TBX21
IL12B
GZMA
CCL5
CD8A
IL12A
CD8B
EOMES
PRF1
GZMB
Pearson r
TNBC MB
TNBC TCGA
HER2+ MB
ER/PR+ MB
HER2+ TCGA
ER/PR+ TCGA
E
Neutrophils
Mast cells
Dendritic cells
Tregs
Macrophages
Exhausted CD8+ T cells
Cytotoxic cells
B cells
Th1 cells
NK cells
CD56dim NK cells
T cells
CD8+ T cells
TGCT
LUAD
HNSC
UCEC
PSKCM
SARC
KIRC
CESC
LUSC
STAD
ESCA
MSKCM
GBM
PAAD
BLCA
OV
LAML
THYM
READ
PCPG
PRAD
KIRP
LGG
COAD
BRCA
LIHC
THCA
Pearson r
F
Fibroblasts
Endothelial cells
Neutrophils
CD8+ T cells
Myeloid dendritic cells
T cells
Monocytic lineage
NK cells
B cell lineage
Cytotoxic lymphocytes
Neutrophils
Mast cells
Dendritic cells
Tregs
Macrophages
Exhausted CD8+ T cells
Cytotoxic cells
B cells
Th1 cells
NK cells
CD56dim NK cells
T cells
CD8+ T cells
TNBC MB
TNBC TCGA
HER2+ MB
ER/PR+ MB
HER2+ TCGA
ER/PR+ TCGA
Pearson r

**Figure S6. (Related to Figure 5 and Figure 6) COX-2 expression delineates cancer-promoting from cancer-inhibitory inflammation in human breast cancer subtypes.** Survival analysis of LUAD (TCGA, n=512) and CESC (TCGA, n=305) patients stratified according to the COX-IS. (A) Kaplan-Meier survival plots parsed as high versus low COX-IS expressors at a 75% stringency. (B) Forest plots showing a multivariate Cox regression analysis for the indicated risk factors. (C) Hazard ratio associated with the indicated gene signatures or the individual gene elements of the COX-IS. Hazard ratio (95% C.I.), Log-rank (Mantel-Cox) test (A-C). (D) Heatmap showing the Pearson correlation coefficient of *PTGS2* expression with the mouse-derived COX-IS genes across the different breast cancer datasets from TCGA: TNBC (n=115), HER2+ (n=195), ER/PR+ (n=666); and METABRIC: TNBC (n=251), HER2+ (n=205), ER/PR+ (n=968). (E, F) Pearson correlation coefficients of *PTGS2* expression with the indicated cell populations as defined by Danaher et al. (E and F, right panel) or by the MCP counter algorithm (F, left panel), for all datasets shown in Figure 5A and S6D.

Figure S7

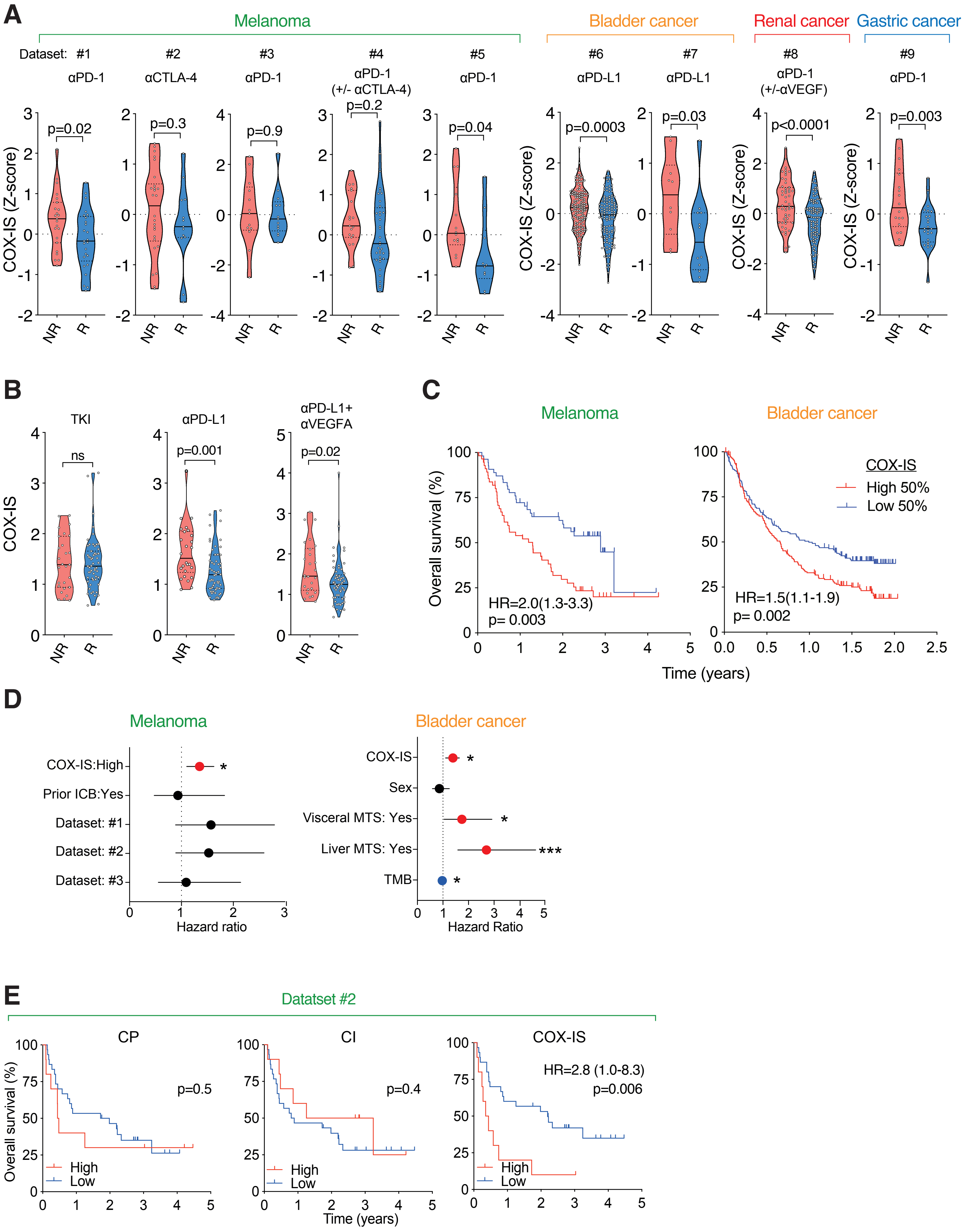
A
Melanoma
Bladder cancer
Renal cancer
Gastric cancer
Dataset: #1
#2
#3
#4
#5
#6
#7
#8
#9
αPD-1
αCTLA-4
αPD-1
αPD-1 (+/- αCTLA-4)
αPD-1
αPD-L1
αPD-L1
αPD-1 (+/-αVEGF)
αPD-1
p=0.02
p=0.3
p=0.9
p=0.2
p=0.04
p=0.0003
p=0.03
p<0.0001
p=0.003
COX-IS (Z-score)
NR
R
B
TKI
αPD-L1
αPD-L1+ αVEGFA
ns
p=0.001
p=0.02
COX-IS
C
Melanoma
Bladder cancer
Overall survival (%)
HR=2.0(1.3-3.3)
p= 0.003
HR=1.5(1.1-1.9)
p= 0.002
COX-IS
High 50%
Low 50%
Time (years)
D
Melanoma
Bladder cancer
COX-IS:High
Prior ICB:Yes
Dataset: #1
Dataset: #2
Dataset: #3
Hazard ratio
COX-IS
Sex
Visceral MTS: Yes
Liver MTS: Yes
TMB
Hazard Ratio
E
Datatset #2
CP
CI
COX-IS
p=0.5
p=0.4
HR=2.8 (1.0-8.3)
p=0.006
High
Low
Overall survival (%)
Time (years)

**Figure S7. (Related to Figure 7) The COX-IS associates with outcome from ICB.** (A) Analysis of COX-IS (computed from z-score normalized gene expression) at baseline in R and NR groups of patients reported in Figure 7A. (B) Analysis of COX-IS at baseline in R and NR patients from dataset #8 divided accordingly to the treatment received: TKI: sunitinib; αPD-L1: atezolizumab; αVEGF: bevacizumab. (C) Kaplan-Meier survival plots for melanoma (pooled datasets #1, #2, #3 and #4) and bladder cancer (dataset #6) parsed as high versus low on a median cut-off for baseline COX-IS. (D) Multivariate Cox regression analysis for the indicated risk factors in melanoma (pooled datasets #1, #2, #3 and #4) and bladder cancer (dataset #6) patients. (E) Survival analysis of patients from dataset #2 stratified based on the CP, CI signatures or the COX-IS.
